# Nickelaelectro-Catalyzed Glycosyl-Donor Activation Enabling α‑*C*‑Alkenyl Glycoside Assembly

**DOI:** 10.1021/acs.orglett.5c03327

**Published:** 2025-10-09

**Authors:** Fabian Hinrichs, Rajeshwaran Purushothaman, Lutz Ackermann

**Affiliations:** † Wöhler Research Institute for Sustainable Chemistry (WISCh), 9375 Georg-August-Universität Göttingen, Tammannstraße 2, 37077 Göttingen, Germany; ‡ German Center for Cardiovascular Research (DZHK), Potsdamer Straße 58, 10785 Berlin, Germany

## Abstract

*C*-alkenyl glycosides represent an important class of carbohydrates that includes numerous biologically active compounds. We developed a stereoselective and sustainable strategy using anomeric glycosyl halides as radical precursors for reductive alkenylation. This enables efficient anomeric functionalization with excellent α-selectivity through ligand control. Electrochemical halogen-atom transfer (*e*-XAT) activates the glycosyl donors, avoiding stoichiometric metal reductants and expensive photocatalysts. This method shows broad substrate scope, including diverse oligosaccharides, delivering high yields and stereoselectivity.

## Graphical abstract

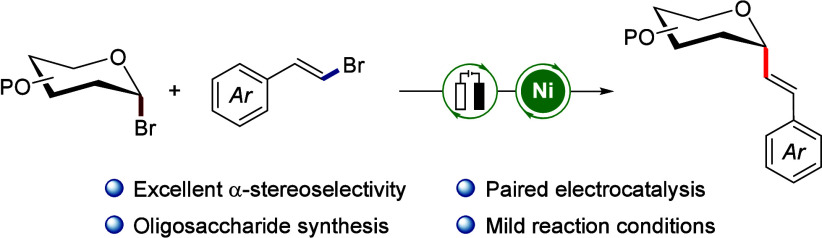

*C*-glycosides featuring a direct carbon–carbon bond at the anomeric position are prevalent scaffolds in prominent natural products, which present notable medicinal value.^1^ Among them, *C*-alkenyl glycosides form a distinct subclass distinguished by a carbon–carbon double bond at the anomeric position, which replaces the traditional C–O glycosidic linkage. This structural modification not only confers enhanced metabolic stability but also provides a synthetically flexible handle for further molecular diversification. These features make *C*-alkenyl glycosides promising target molecules for pharmaceutical and biological applications.^2^ Moreover, anomeric alkenyl linkages are found in various natural products and are commonly utilized as *O*-glycoside mimics for drug development (Scheme 1A).^3^

**Scheme 1:**
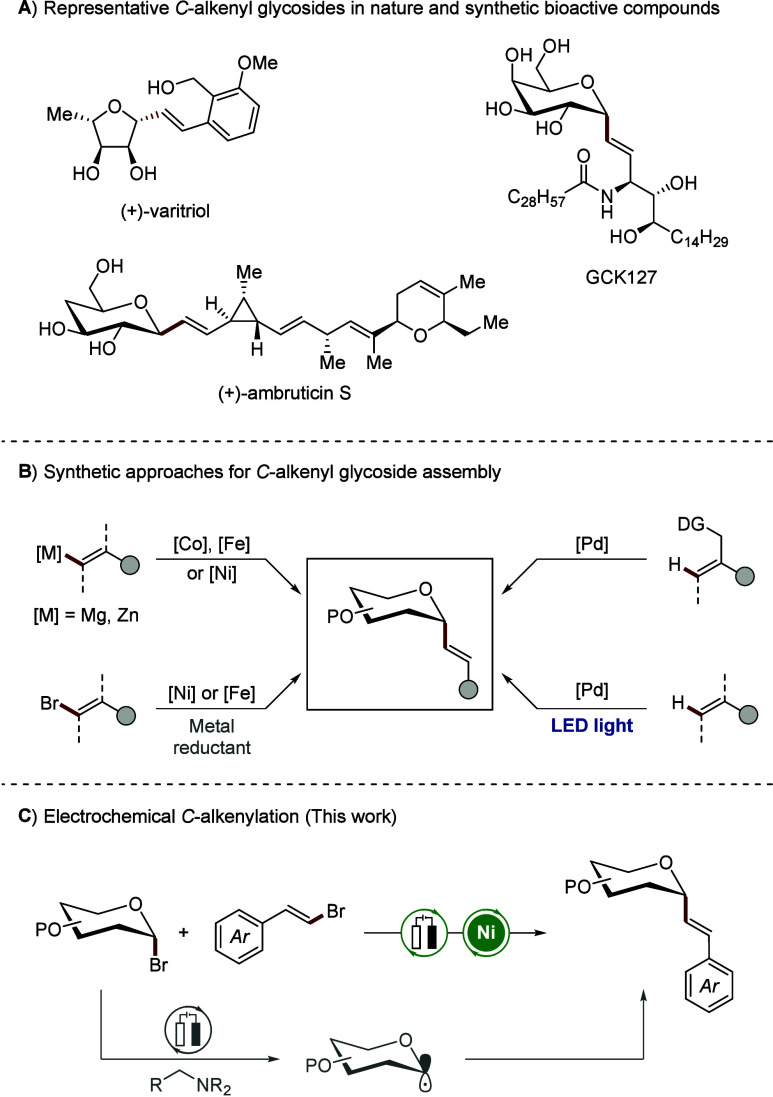
*C*-Alkenyl Glycosides and Their Assembly

Despite their relevance, synthetic access to *C*-alkenyl glycosides remains a significant challenge. While numerous approaches have been established for the efficient preparation of *C*-aryl glycosides,^4^ translating these methods to the alkenyl counterparts has proven less straightforward. These commonly involve transition metal-catalyzed cross-coupling reactions in the presence of organozinc or Grignard reagents (Scheme 1B).^4b,4c,5^ Inspired by such strategies, Gong and co-workers introduced a nickel-catalyzed cross-electrophile coupling, achieving stereoselective arylation and alkenylation of glycosyl halides in the presence of stoichiometric zinc as reductant.^6^ Additionally, Liang^7^ and Olofsson^8^ developed a photocatalytic synthesis of *C*-alkenyl glycosides. Moreover, a palladium catalyzed C–H activation approach was employed to construct *C*-alkenyl glycosides from directing group-bearing olefins, offering an alternative to classical cross-coupling methods.^9^ Recently, Koh’s group reported the use of iron catalysis in the reductive cross-coupling of glycosyl chlorides and alkenyl halides.^10^

Although the synthesis of *C*-alkenyl glycosides has witnessed considerable advances in the field, several bottlenecks persist. These include the reliance on stoichiometric amounts of metal reductants, the need for prefunctionalized glycosyl donors or organometallic coupling partners, and difficulties in achieving high stereoselectivity. In this context, glycosyl halides, which are commercially available and commonly used as glycosyl donors, traditionally required such harsh and unsustainable reaction conditions for the effective construction of anomeric C–C bonds.^11^ Electrochemical strategies have recently emerged as powerful tools in organic synthesis, offering sustainable alternatives to traditional redox processes and enabling diverse bond constructions under mild conditions.^12^ Amid these advancements, our group developed a novel electrochemically driven methodology that activates glycosyl bromides via electrochemical halogen atom transfer (*e*-XAT), initiated by an α-amino alkyl radical generated from *N*,*N*-diisopropylethylamine (DIPEA).^13^ This approach enabled the efficient generation of glycosyl radicals, promoting the synthesis of *C*-alkyl glycosides from unactivated alkenes with high stereoselectivity. The robustness of this *e*-XAT strategy was demonstrated in the assembly of *C*-aryl as well as *C*-acyl glycosides through nickel electrocatalysis, maintaining excellent anomeric selectivity. By leveraging cathodic reduction to regenerate the active nickel catalyst, this approach avoids the use of stoichiometric metal-based reductants. In line with current efforts to develop more sustainable glycosylation strategies, such as photoredox catalysis,^7,8,14^ our electrochemical platform offers a streamlined, efficient, and environmentally sound route to *C*-alkenyl glycosides (Scheme 1C).

Inspired by our previous findings, we initiated the optimization study toward an electrochemical anomeric coupling of acetate-protected glycosyl bromide **1a** and alkenyl bromide **2a** for the synthesis of the desired *C*-alkenyl glycoside **3a**, employing Ni­(acac)_2_ as the catalyst with the sterically hindered methyl substituted phenanthroline ligand **L1** (Table 1). We utilized a platinum (Pt) anode and graphite felt (GF) cathode in an undivided electrochemical cell, under constant current electrolysis at 4 mA. DIPEA served as both XAT mediator and reductant, while MeCN was used as the solvent. Under these conditions, the desired α-anomeric alkenyl glycoside was obtained in 39% yield with an α:β ratio of 6:1 (entry 1, Table 1). Alongside the desired product **3a**, a side product **4** was also detected, which likely arises via electrochemical reduction followed by β-OAc elimination. The use of different phenanthroline ligands reduced the diastereomeric selectivity, although the unsubstituted phenanthroline **L3** showed an increase in yield of **3a** (entries 2–3, Table 1). Furthermore, we evaluated more sterically hindered substituted bipyridine ligands **L4** and **L5**, which improved α-selectivity albeit with low yields (entries 4–5, Table 1). Other ligands, such as the *t*Bu-Terpy **L6** and dppbz **L7**, failed to deliver satisfactory selectivity or product yield (entries 6–7, Table 1). In contrast, the use of monodentate pyridine ligands (**L8**–**L11**) in an optimized solvent system of MeCN/DMF (9:1) (for optimization details, see Tables S2–S4) led to significantly improved yields and excellent anomeric selectivity for *C*-alkenyl glycoside **3a** (entries 8–9, Table 1).

**Table 1:**
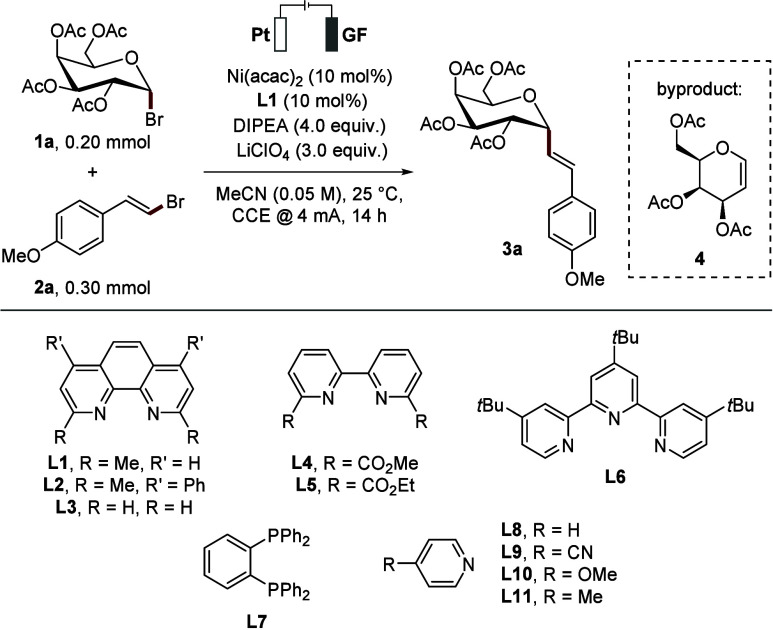
Optimization of the Reaction Conditions

	Deviation from above	Yield **3a** [%]^a^	α:β
1	none	39^b^	6:1
2	**L2**	23	5:1
3	**L3**	52	2:1
4	**L4**	22	10:1
5	**L5**	16	19:1
6	**L6**	42	1:1
7	**L7**	23	9:1
8	**L8**/**L9**/**L10**	68,^c^ ^,^ ^d^/68^c^ ^,^ ^d^/58^c^ ^,^ ^d^	>20:1
9	**L11**	71^b^ ^,^ ^c^ ^,^ ^d^	>20:1
10	*n*Bu_4_NBF_4_ instead of LiClO_4_	35^d^	>20:1
11	*n*Bu_4_NI instead of LiClO_4_	25^d^	>20:1
12	GF(+)/GF(−)	52^d^	>20:1
13	Glycosyl iodide instead of **1a**	8^d^	7:1
14	w/o LiClO_4_	25^d^	>20:1
15	w/o Ni(acac)_2_/DIPEA/electricity	N.D./N.D./N.D.	

a Yields and α:β-ratios determined by ^1^H NMR using 3,4,5-trichloropyridine as internal standard.

b Isolated yield.

c 2.0 equiv of LiClO_4_ was used.

d MeCN/DMF (9:1) as solvent system and 20 mol% ligand. N.D.: not detected.

While unsubstituted pyridine **L8** gave a yield of 68%, substituents on the pyridine proved beneficial, with 4-methylpyridine **L11** affording **3a** in 71% yield and excellent selectivity of α:β > 20:1 (entry 9, Table 1). Notably, several additional ligands were considered during the reaction optimization, which unfortunately led to unfruitful outcomes (Table S2). Control experiments using other electrolytes, such as *n*Bu_4_NBF_4_ and *n*Bu_4_NI (entries 9–10, Table 1), resulted in significantly reduced reactivity compared to LiClO_4_. In efforts to further enhance the reactivity, various electrode materials were evaluated (Table S4). However, the Pt­(+)/GF(−) combination consistently gave superior results. The reaction was also compatible with a GF­(+)/GF(−) electrode setup, albeit with only a moderate yield of the α-anomeric alkenyl glycoside **3a** (entry 12, Table 1). Replacing the glycosyl donor **1a** with the corresponding glycosyl iodide led to a poor outcome (entry 13, Table 1). Control experiments confirmed that the catalyst, DIPEA, and electricity were all crucial for the desired transformations (entry 15, Table 1). Although the reaction proceeded without electrolyte, only poor reactivity was observed (entry 14, Table 1).

With the optimized reaction conditions in hand (entry 9, Table 1), we proceeded to demonstrate the robustness of the reaction (Scheme 2). In addition to the *para*-methoxy functionalized model substrate **2a**, a variety of alkenyl bromides were coupled with glycosyl bromide **1a**, yielding **3b**–**3g** in good yields with excellent α:β selectivity (>20:1) throughout. Biologically relevant heterocyclic alkenyl bromides, such as pyridine (**3h**) and pyrimidine (**3i**), were successfully obtained in 67% and 62% yields, respectively. Interestingly, unprotected indole was also well tolerated under the reaction conditions, affording product (**3j**) in 48% yield without compromising stereoselectivity. We further explored the scope of the electrochemical *e*-XAT using a variety of glycosyl bromides bearing different protecting groups. These substrates were generally well tolerated, affording the desired *C*-alkenyl glycosides in moderate to good yields with excellent stereoselectivity. Along with the acetyl-protected galactose, other monosaccharides such as acetyl-protected glucose (**3l**) and rhamnose (**3m**) afforded the products in 74% and 67% yield, respectively, with glucose showing a slightly reduced α:β selectivity of 5:1. Mannose bromide proved also compatible, delivering the product **3k** in lower yield but with excellent α-selectivity. This difference in α-selectivity arises from the radicals’ conformations: mannose and galactose preferentially adopt rather rigid geometries (chair and half-chair, respectively) that favor an axial coupling, while glucose predominantly features a flexible B_2,5_ boat conformation, hence translating into a reduced diastereoselectivity.^15^ Benzoyl-protected rhamnose (**3n**) and galactose (**3o**) gave yields of 43% and 74%, respectively. Moreover, pivaloyl-protected glucose bromide furnished the corresponding product **3p** in 74% yield, confirming the broad compatibility of the method with diverse protecting groups without compromising anomeric selectivity. Encouraged by these results, we next evaluated disaccharide substrates. Glycosyl bromide derived from maltose afforded the *C*-alkenyl glycoside (**3q**) in 59% with a selectivity of α:β = 10:1. Furthermore, acetylated disaccharides cellobiose (**3r**), lactose (**3s**), melibiose (**3t**) and isomaltose (**3u**) were also compatible substrates, providing the corresponding α-*C*-alkenyl glycosides in good yields (60–71%) and moderate selectivity up to α:β = 6:1. Notably, the trisaccharide maltotriose (**3v**) proved to be a competent substrate, affording the desired product in 55% yield with excellent α-selectivity (α:β > 20:1). Furthermore, late-stage drug-derived glycosyl bromides from probenecid (**3w**) and diclofenac (**3x**) were well tolerated under standard reaction conditions, providing the corresponding products in 63% and 55% yields, respectively, with excellent stereoselectivity.

**Scheme 2:**
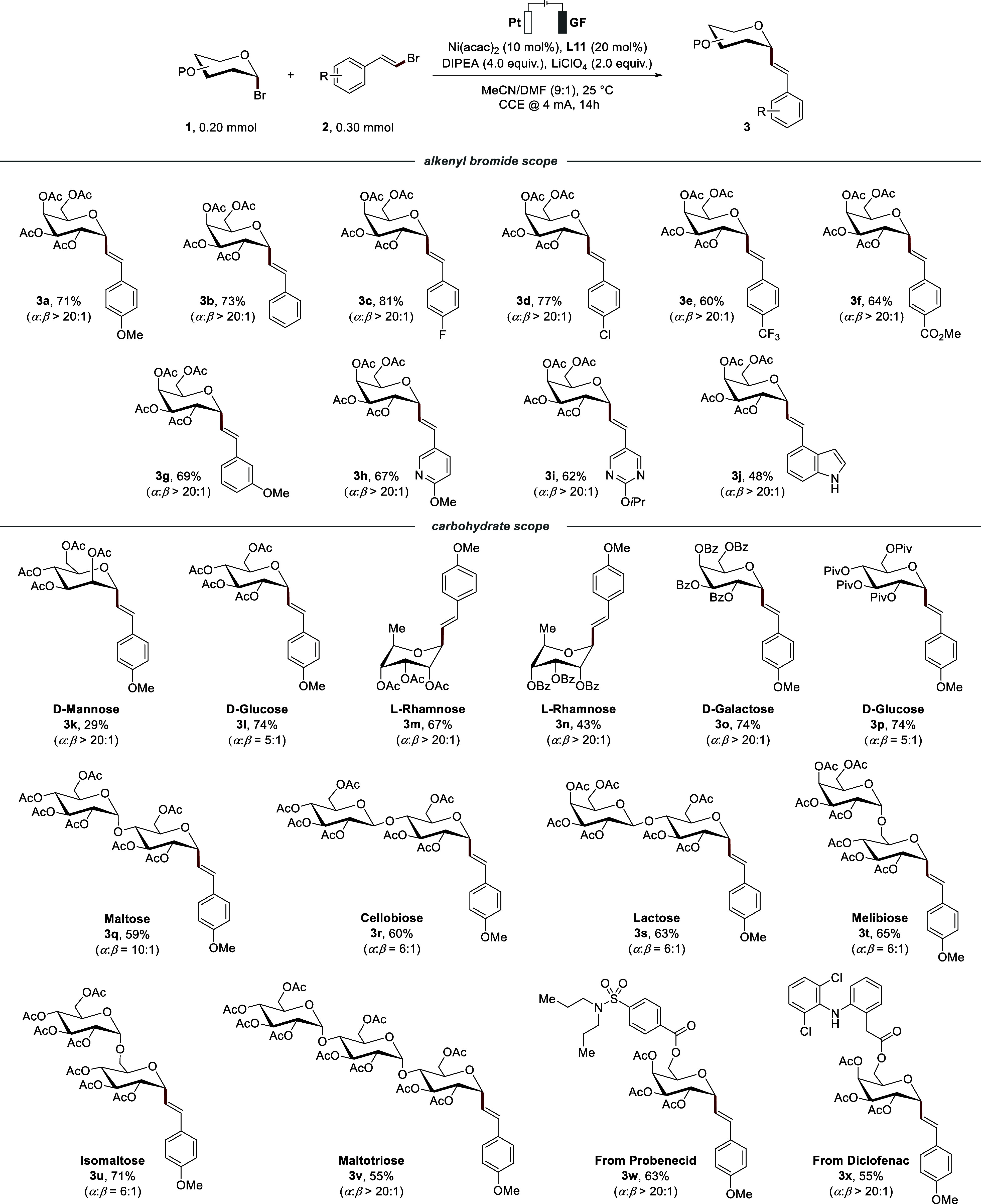
Scope of Alkenyl Bromides and Glycosyl Bromides

Based on previous mechanistic investigations on nickelaelectro-catalyzed reductive couplings involving glycosyl bromides and aryl iodides, the reaction is proposed to proceed via a radical pathway (Scheme 3).^6a,13a,13b^ The glycosyl radical is generated through an electrochemical halogen-atom transfer (*e*-XAT), initiated by anodic oxidation of the Hünig base (DIPEA) to produce an α-amino alkyl radical. This transient species then promotes single-electron transfer to the glycosyl bromide **1**, yielding the corresponding glycosyl radical. The ensuing reductive alkenylation proceeds through a nickel (0/II/III/I) catalytic cycle. Oxidative addition of the alkenyl bromide **2** to the nickel(0) species furnishes a nickel­(II) complex, which subsequently intercepts the glycosyl radical to generate a high-valent nickel­(III) intermediate. Reductive elimination from this species delivers the target *C*-alkenyl glycoside. The resulting nickel­(I) species is electrochemically reduced at the cathode, thereby regenerating the nickel(0) catalyst and closing the catalytic cycle.

**Scheme 3:**
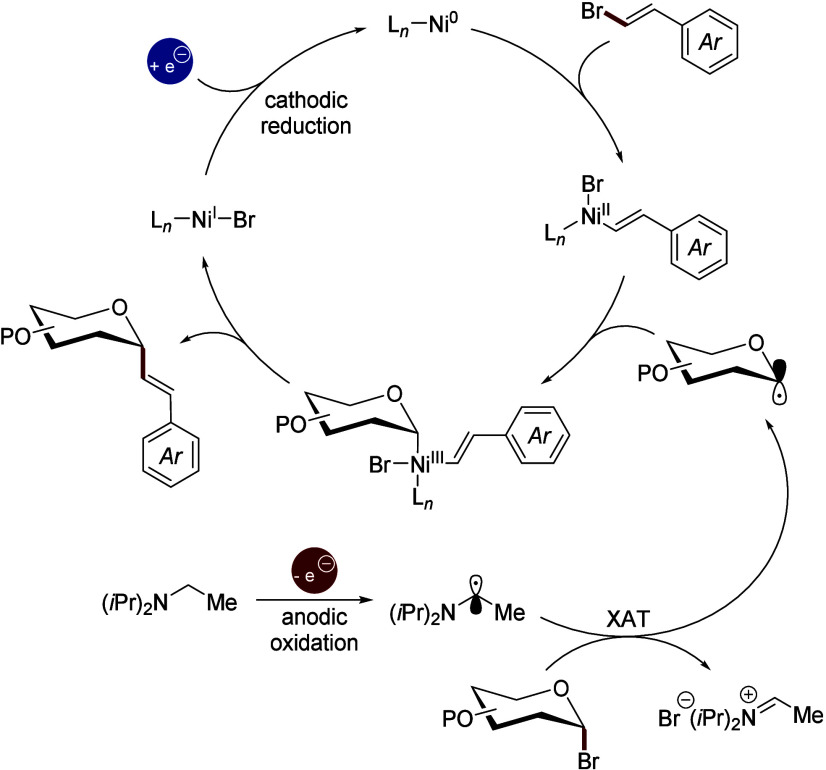
Proposed *e-*XAT-Based Catalytic Cycle

We disclosed, herein, a nickel-paired electrocatalytic strategy for the efficient and sustainable synthesis of α-*C*-alkenyl glycosides. This strategy leverages an electrochemical halogen-atom transfer (*e*-XAT) approach, enabling the activation of glycosyl bromides under mild conditions without the need for stoichiometric metal reductants and expensive photocatalysts. While XAT approaches have previously been applied to aryl halides, our findings demonstrate that alkenyl halides are also viable coupling partners for anomeric functionalization. Our strategy exhibits ample substrate compatibility, accommodating a variety of glycosyl donors, including various oligosaccharides with diverse protecting groups and delivering the desired *C*-alkenyl glycosides in good yields with excellent α-selectivity.

## Supplementary Material



## Data Availability

The data underlying this study are available in the published article and its Supporting Information.

## Notes

Details of optimization, experimental procedures, characterization of all the products, and NMR spectra (PDF)
