# Supplementary material for: Nickelaelectro-Catalyzed Glycosyl-Donor Activation Enabling α‑C‑Alkenyl Glycoside Assembly
Source: Org Lett. 2025 Oct 9;27(42):11714–8. doi: 10.1021/acs.orglett.5c03327 (PMC12560081; doi:10.1021/acs.orglett.5c03327)
Supplement: Supplementary file 1 [file ol5c03327_si_001.pdf]

Supplementary materials for

Nickelaelectro-Catalyzed Glycosyl-Donor Activation enabling *α*-*C*-Alkenyl Glycoside Assembly

Fabian Hinrichs [a, +], Rajeshwaran Purushothaman [a, +], Lutz Ackermann [a, b, *]

[a] *Wöhler Research Institute for Sustainable Chemistry (WISCh), Georg-August-Universität Göttingen, Tammannstraße 2, 37077 Göttingen, Germany.*

[b] *German Center for Cardiovascular Research (DZHK), Potsdamer Straße 58, 10785 Berlin, Germany.*

* *Corresponding author: Lutz.Ackermann@chemie.uni-goettingen.de.*

[+] *These authors contributed equally to this work*

Table of Contents

I. Experimental Section 1

General Information 1

Optimization of Reaction Conditions 2

Scope of Glycosyl Bromides 6

Preparation of Alkenyl Bromides 6

Nickelaelectro-Catalyzed Synthesis of *α*-*C*-Alkenyl Glycosides 10

II. Spectral Data for Compounds 30

III. References 59

I. Experimental Section

General Information

All solvents used for the reactions were HPLC grade. All commercially available chemicals were purchased from commercial suppliers and were used as received. Catalytic reactions were performed under an N_2 atmosphere using pre-dried glassware and standard Schlenk techniques. ^{1}H-, ^{13}C- and ^{19}F-NMR spectra were measured at ambient temperature using 5 mm diameter NMR tubes. ^{13}C-NMR spectra were proton decoupled. Coupling constants (*J*) are reported in Hertz. For ^{1}H-NMR yields, 3,4,5-trichloropyridine was used as an internal standard. Structural assignments were made with additional information from gCOSY-, gHSQC- and gHMBC-experiments. Electrocatalysis was conducted using a Metrohm MULTI AUTOLAB M204 potentiostat in constant current mode. Platinum electrodes (99.95% Pt, 25 × 10 × 0.125 mm, electrical conductivity: 9.43 MS/m; ESG Edelmetall-Handel GmbH & Co. KG, Rheinstetten, Germany) and graphite felt (GF) electrodes (type GFA6; SGL Carbon GmbH, Meitingen, Germany) were connected using stainless steel adapters. Yields refer to isolated compounds, estimated to be >95% pure as determined by ^{1}H-NMR. Column chromatography was performed using silica gel 60 (40–63 µm) from Merck. NMR spectra were recorded on a Bruker Avance III 300, Bruker Avance Neo 300, Bruker Avance III HD 400, Bruker Avance III HD 500, or Bruker Avance Neo 600 in the solvent indicated; chemical shifts (δ) are given in ppm relative to the residual solvent peak. All IR spectra were recorded on a Bruker FT-IR Alpha device. High resolution mass spectrometry (HRMS) was measured with APEX IV 7T FTICR.

Optimization of Reaction Conditions

Table S1. General Optimization

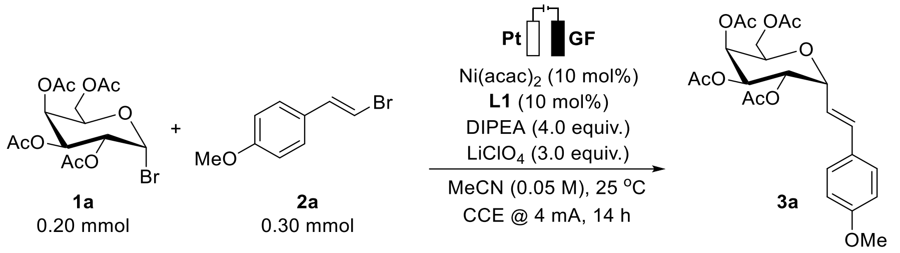

Entry	Deviation from above	Yield **3a** [%][a]	α:β
1	none	39[b]	6:1
2	**L2**	23	5:1
3	**L3**	52	2:1
4	**L4**	22	10:1
5	**L5**	16	19:1
6	**L6**	42	1:1
7	**L7**	23	9:1
8	**L8** / **L9** / **L10**	68[c,d] / 68[c,d] / 58[c,d]	>20:1
9	**L11**	71[b,c,d]	>20:1
10	nBu$_4$NBF$_4$ instead of LiClO$_4$	35[d]	>20:1
11	nBu$_4$NI instead of LiClO$_4$	25[d]	>20:1
12	GF(+)/GF(-)	52[d]	>20:1
13	Glycosyl iodide instead of **1a**	8[d]	7:1
14	w/o LiClO$_4$	25[d]	>20:1
15	w/o Ni(acac)$_2$ / DIPEA / electricity	N.D. / N.D. / N.D.	-

[a] Yields and α:β-ratios were determined by ^{1}H-NMR using 3,4,5-trichloropyridine as internal standard. [b] Isolated yield. [c] 2.0 equiv. of LiClO$_4$ were used. [d] MeCN/DMF (9:1) as solvent system and 20 mol% ligand. N.D.: not detected.

Table S2. Optimization with different Ligands

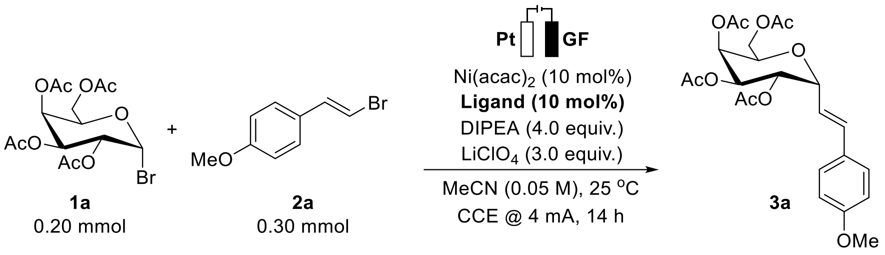

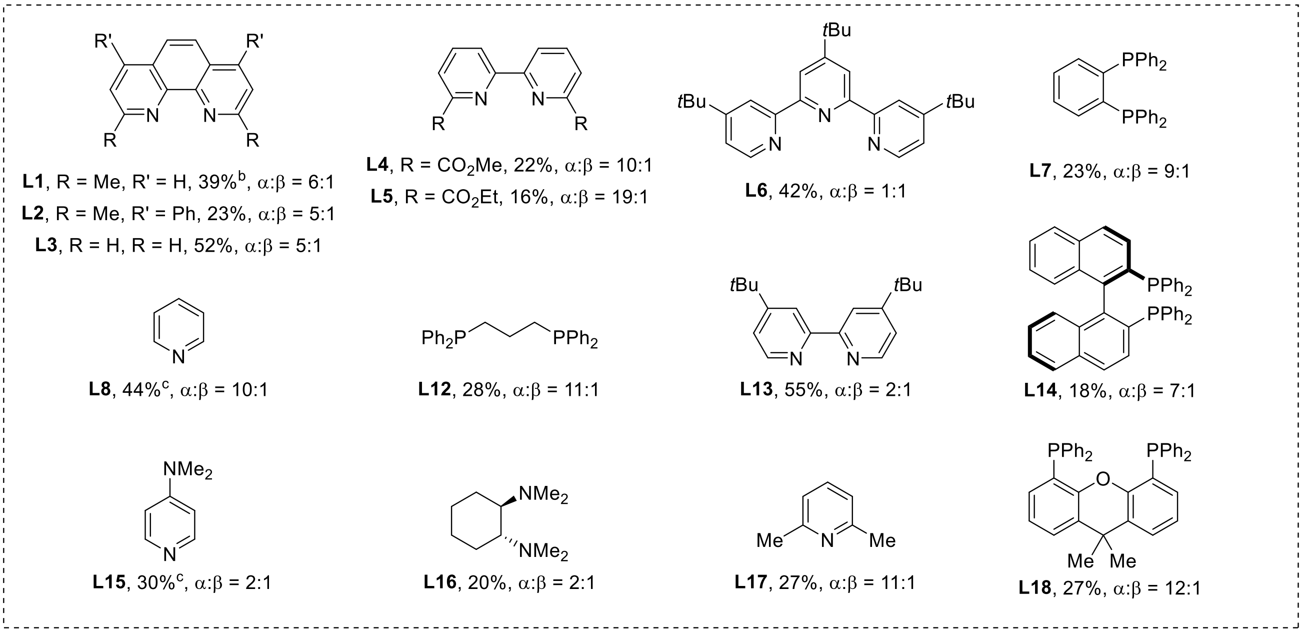

[a] Yields and *α*:*β*-ratios were determined by ^{1}H-NMR using 3,4,5-trichloropyridine as internal standard. [b] Isolated yield. [c] 20 mol% of ligand was used. **L1–L18** were purchased from commercial suppliers and were used as received.

Table S3. Optimization of amounts of Substrates and Bases

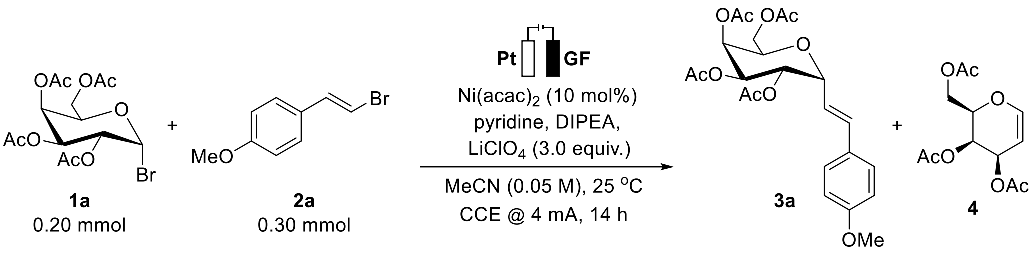

Entry	1a [equiv.]	2a [equiv.]	pyridine [mol%]	DIPEA [equiv.]	Yield 3a [%][a]	Yield 4 [%][a]	*α*:*β*
1	1.0	1.5	20	4.0	44	32	10:1
2	1.0	1.0	20	4.0	37	43	13:1
3	1.5	1.0	20	4.0	43	70	14:1
4	1.0	1.5	50	4.0	42	22	8:1
5	1.0	1.5	100	4.0	43	21	5:1
6	1.0	1.5	20	3.0	38	37	13:1
7	1.0	1.5	20	2.0	29	24	>20:1

[a] Yields and *α*:*β*-ratios were determined by ^{1}H-NMR using 3,4,5-trichloropyridine as internal standard. 1.0 equiv. = 0.20 mmol.

Table S4. Optimization of other Parameters

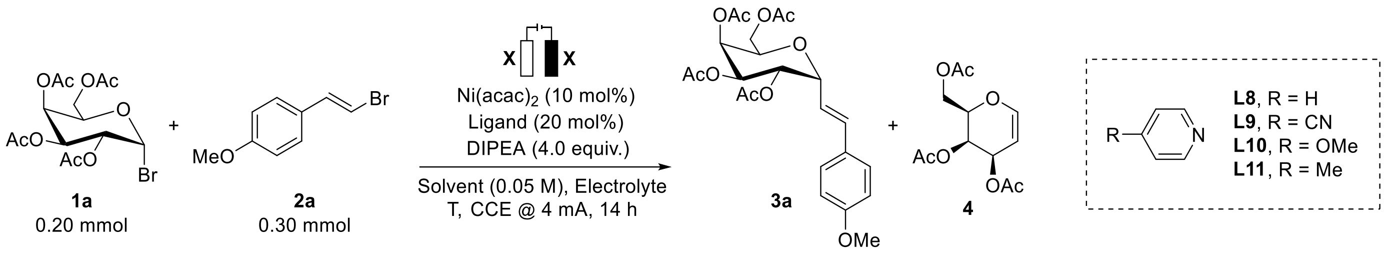

Entry	Temp. [°C]	Solvent	Electrodes (+/-)	Electrolyte	Ligand	Yield 3a [%][a]	Yield 4 [%][a]	*α*:*β*
1	0	MeCN	Pt/GF	$LiClO_4$ (3.0 equiv.)	**L8**	27	28	13:1
2	25	MeCN	Pt/GF	$LiClO_4$ (3.0 equiv.)	**L8**	44	32	10:1
3	40	MeCN	Pt/GF	$LiClO_4$ (3.0 equiv.)	**L8**	18	19	9:1
4	25	DMF	Pt/GF	$LiClO_4$ (3.0 equiv.)	**L8**	22	22	>20:1
5	25	MeCN/DMF (1/1)	Pt/GF	$LiClO_4$ (3.0 equiv.)	**L8**	39	22	>20:1
6	25	MeCN/DMF (3/1)	Pt/GF	$LiClO_4$ (3.0 equiv.)	**L8**	54	17	>20:1
7	25	MeCN/DMF (5/1)	Pt/GF	$LiClO_4$ (3.0 equiv.)	**L8**	60	16	>20:1
8	25	MeCN/DMF (9/1)	Pt/GF	$LiClO_4$ (3.0 equiv.)	**L8**	63	14	>20:1
9	25	MeCN/DMF (19/1)	Pt/GF	$LiClO_4$ (3.0 equiv.)	**L8**	55	21	>20:1
10	25	MeCN/DMF (9/1)	Pt/glassy C	$LiClO_4$ (3.0 equiv.)	**L8**	26	28	15:1
11	25	MeCN/DMF (9/1)	Pt/Ni foam	$LiClO_4$ (3.0 equiv.)	**L8**	4	traces	-
12	25	MeCN/DMF (9/1)	GF/GF	$LiClO_4$ (3.0 equiv.)	**L8**	52	12	>20:1
13	25	MeCN/DMF (9/1)	GF/GF	$LiClO_4$ (3.0 equiv.)	**L8**	52[c]	17	>20:1
14	25	MeCN/DMF (9/1)	GF/GF	$LiClO_4$ (3.0 equiv.)	**L8**	24[d]	12	14:1
15	25	MeCN/DMF (9/1)	Pt/GF	*n*Bu_4NBF_4 (3.0 equiv.)	**L8**	35	32	>20:1
16	25	MeCN/DMF (9/1)	Pt/GF	*n*Bu_4NI (3.0 equiv.)	**L8**	25	14	>20:1
17	25	MeCN/DMF (9/1)	Pt/GF	$LiClO_4$ (2.0 equiv.)	**L8**	68	14	>20:1
18	25	MeCN/DMF (9/1)	Pt/GF	$LiClO_4$ (1.0 equiv.)	**L8**	66	13	>20:1
19	25	MeCN/DMF (9/1)	Pt/GF	$LiClO_4$ (2.0 equiv.)	**L9**	68	10	>20:1
20	25	MeCN/DMF (9/1)	Pt/GF	$LiClO_4$ (2.0 equiv.)	**L10**	58	18	>20:1
21	25	MeCN/DMF (9/1)	Pt/GF	$LiClO_4$ (2.0 equiv.)	**L11**	71[b]	14[b]	>20:1

[a] Yields and *α*:*β*-ratios were determined by ^{1}H-NMR using 3,4,5-trichloropyridine as internal standard. [b] Isolated yield. [c] 8 mA. [d] Alternating current (0.1 Hz).

Scope of Glycosyl Bromides

Compounds **1a–1o** were synthesized according to reported procedure.[1]

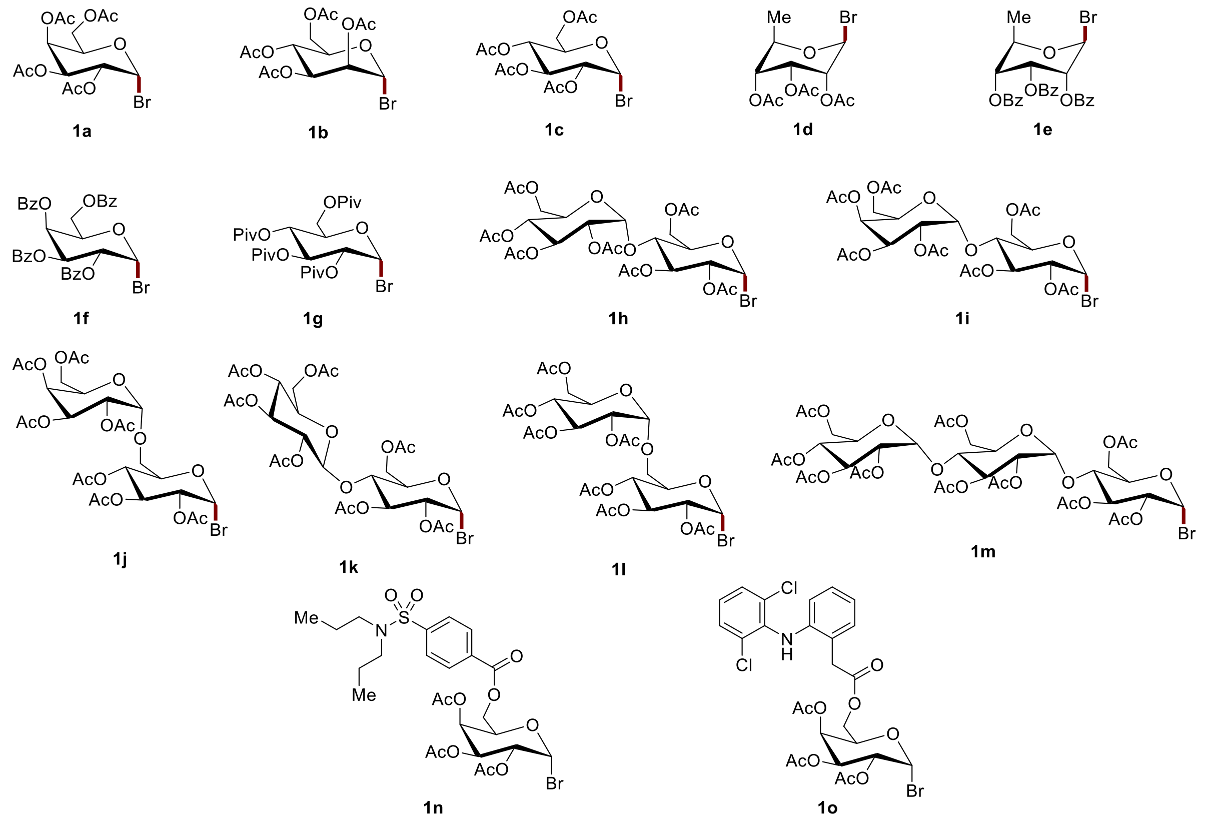

Scope Limitations

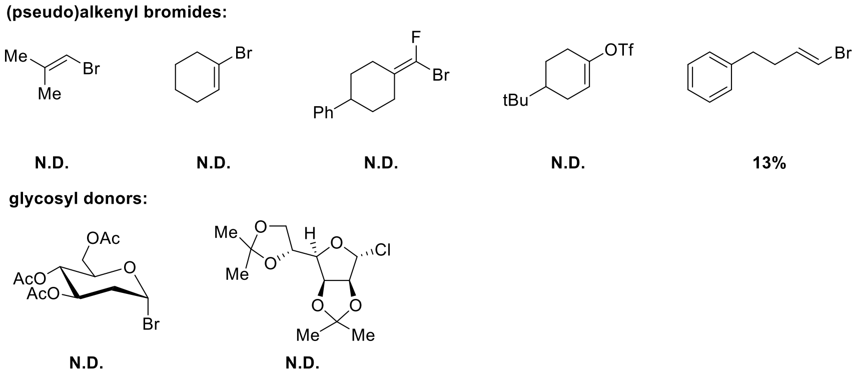

Preparation of Alkenyl Bromides

(*E*)-(2-bromoalkenyl)benzene (2b)[2]

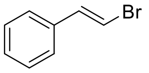

To a solution of cinnamic acid (1.0 equiv., 2.96 g, 20.0 mmol) and $Mn(OAc)_2 \cdot 4H_2O$ (0.2 equiv., 980 mg, 4.00 mmol) in a solvent mixture of H_2O/MeCN (1:1, 40 mL) was added *N*-Bromosuccinimide (1.05 equiv., 3.74 g, 21.0 mmol). After stirring the reaction mixture for 4h at room temperature, MeCN was evaporated under reduced pressure and the mixture was extracted with

hexane (3 x 30 mL). The organic layers were then washed with brine (50 mL) and dried over $MgSO_4$. After evaporation of the solvent the residue was further purified by flash column chromatography (SiO_2, hexane) to yield the product **2b** as a colorless oil (1.89 g, 10.3 mmol, 52%). Analytical data were in accordance with those reported in the literature.[3]

(*E*)-1-(2-Bromoalkenyll)-4-methoxybenzene (2a)[4]

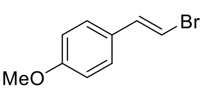

Triethylamine (5 mol%) was added to a solution of (*E*)-3-(4-methoxyphenyl) acrylic acid (3.56 g, 20.0 mmol) in CH_2Cl_2 (60 mL). After the solution was stirred for 5 min at room temperature, *N*-Bromosuccinimide (4.27 g, 24.0 mmol) was added portion wise and the solution was left stirring for another 30 min. The solvent was removed under reduced pressure and the crude residue was purified by flash column chromatography (SiO_2, hexane with 1% EtOAc) to yield the alkenyl bromide **2a** as a colorless solid (3.80 g, 89%). Analytical data were in accordance with those reported in the literature.[3]

General Procedure 1: Synthesis of (*E*)-alkenyl bromide from benzaldehydes.

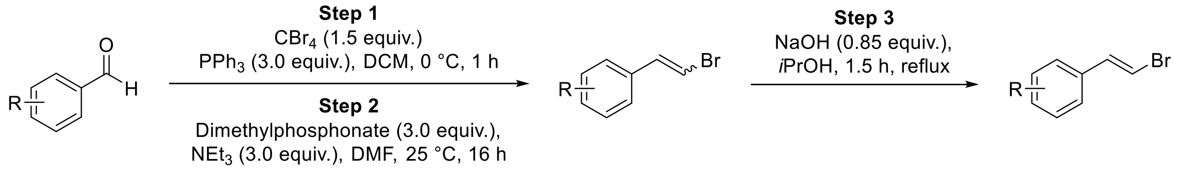

***Step 1*: Ramirez protocol for the Wittig-type dibromoolefination.**[5]

To a solution of aldehyde (1.0 equiv.) and CBr_4 (1.5 equiv.) in CH_2Cl_2 (80 mL) was added dropwise a solution of PPh_3 (3.0 equiv.) in CH_2Cl_2 (70 mL) at 0 °C over 30 min. The reaction mixture was then stirred for an additional 1h at 0 °C. Upon completion, the crude mixture was concentrated under reduced pressure, and hexane/EtOAc (5:1, 100 mL) was added. The resulting suspension was filtered through a short silica plug. The filtrate was collected, and the solvent was removed under reduced pressure to afford the crude dibromide, which was used directly in the next step without further purification.

***Step 2*: Hayes protocol for the Hirao reaction.**[6]

To a solution of crude dibromide (1.0 equiv.) and NEt_3 (3.0 equiv.) in DMF (20 mL) was added dimethylphosphonate (3.0 equiv.). The resulting mixture was stirred overnight at room temperature. Distilled H_2O (50 mL) was then added, and the mixture was extracted with CH_2Cl_2 (3 × 50 mL). The combined organic layers were washed successively with 1 M HCl (50 mL) and brine (50 mL), dried over $MgSO_4$, filtered, and concentrated under reduced pressure to afford a mixture of *E*/*Z*-isomers of corresponding alkenyl bromide.

***Step 3*: Dolbys protocol for the selective destruction of the (*Z*)-isomer.**[7]

NaOH (0.85 equiv.) was added to a solution of the crude isomeric mixture (1.0 equiv.) in *i*PrOH (20 mL) and the resulting mixture was refluxed for 1.5 hours. After cooling to ambient temperature, CH_2Cl_2 (100 mL) was added, and the organic layer was washed successively with distilled H_2O (75 mL) and 1 M HCl (75 mL). The organic layer was dried over $MgSO_4$, filtered, and concentrated under reduced pressure. The crude residue was purified by flash column chromatography (SiO_2) to afford the pure (*E*)-alkenyl bromide derivatives.

(*E*)-1-(2-Bromoalkenyl)-4-fluorobenzene (2c)

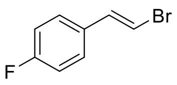

Substrate **2c** was prepared according to *General Procedure 1* using 4-fluorobenzaldehyd (2.48 g, 20.0 mmol). The crude residue was purified by flash column chromatography (SiO_2, hexane) to yield (*E*)-alkenyl bromide as a colorless solid (2.38 g, 11.9 mmol, 59% over 3 steps). Analytical data were in accordance with those reported in the literature.[8]

(*E*)-1-(2-Bromoalkenyl)-4-chlorobenzene (2d)

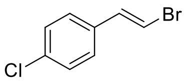

Substrate **2d** was prepared according to *General Procedure 1* using 4-chlorobenzaldehyd (2.81 g, 20.0 mmol). The crude residue was purified by flash column chromatography (SiO_2, hexane) to yield (*E*)-alkenyl bromide as a colorless solid (2.71 g, 12.5 mmol, 62% over 3 steps). Analytical data were in accordance with those reported in the literature.[8]

(*E*)-1-(2-Bromoalkenyl)-4-(trifluoromethyl)benzene (2e)

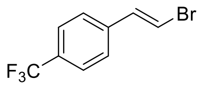

Substrate **2e** was prepared according to *General Procedure 1* using 4-(trifluoromethyl)benzaldehyd (3.48 g, 20.0 mmol). The crude residue was purified by flash column chromatography (SiO_2, hexane) to yield (*E*)-alkenyl bromide as a colorless oil (3.26 g, 13.0 mmol, 65% over 3 steps). Analytical data were in accordance with those reported in the literature.[8]

Methyl (*E*)-4-(2-bromoalkenyl)benzoate (2f)

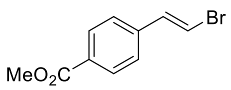

Substrate **2f** was prepared according to *General Procedure 1* using methyl 4-formylbenzoate (3.28 g, 20.0 mmol). The crude residue was purified by flash column chromatography (SiO_2, hexane + 1% EtOAc) to yield (*E*)-alkenyl bromide as a colorless solid (3.49 g, 14.5 mmol, 72% over 3 steps). Analytical data were in accordance with those reported in the literature.[8]

(*E*)-1-(2-Bromoalkenyl)-3-methoxybenzene (2g)

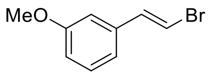

Substrate **2g** was prepared according to *General Procedure 1* using 3-methoxybenzaldehyd (2.72 g, 20.0 mmol). The crude residue was purified by flash column chromatography (SiO_2, hexane + 1% EtOAc) to yield (*E*)-alkenyl bromide as a yellow oil (2.94 g, 13.8 mmol, 69% over 3 steps). Analytical data were in accordance with those reported in the literature.[9]

(*E*)-5-(2-bromoalkenyl)-2-methoxypyridine (2h)

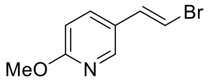

Substrate **2h** was prepared according to *General Procedure 1* using 6-methoxynicotinaldehyde (2.74 g, 20.0 mmol). The crude residue was purified by flash column chromatography (SiO_2, hexane + 1% EtOAc) to yield (*E*)-alkenyl bromide as a yellow oil (2.28 g, 10.7 mmol, 54% over 3 steps).

(*E*)-5-(2-bromoalkenyl)-2-isopropoxypyrimidine (2i)

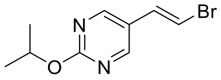

Substrate **2i** was prepared according to *General Procedure 1* using 2-methoxypyrimidine-5-carbaldehyde (2.76 g, 20.0 mmol). The crude residue was purified by flash column chromatography (SiO_2, hexane + 1% EtOAc) to yield (*E*)-alkenyl bromide as a colorless solid (1.46 g, 6.0 mmol, 30% over 3 steps).

(*E*)-4-(2-bromoalkenyl)-1*H*-indole (2j)

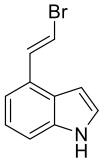

Substrate **2i** was prepared according to *General Procedure 1* using tert-butyl 4-formyl-1H-indole-1-carboxylate (4.29 g, 17.5 mmol). The crude residue was purified by flash column chromatography (SiO_2, hexane/EtOAc = 2/1) to yield (*E*)-alkenyl bromide as a yellow oil (1.95 g, 8.8 mmol, 44% over 3 steps). Analytical data were in accordance with those reported in the literature.[10]

Nickelaelectro-Catalyzed Synthesis of *α*-*C*-Alkenyl Glycosides

General Procedure 2:

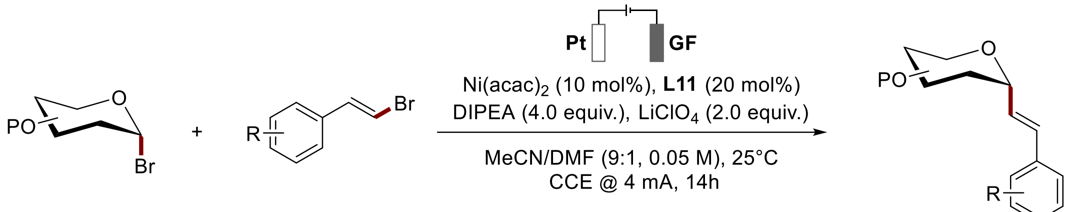

1, 0.20 mmol **2**, 0.30 mmol **3**

A flame-dried Schlenk tube (undivided electrochemical cell) equipped with a stirring bar, platinum electrode (25 × 10 × 0.125 mm) and graphite felt electrode (25 × 10 × 1.5 mm) was charged with glycosyl bromide (0.20 mmol, 1.0 equiv.), alkenyl bromide (0.30 mmol, 1.5 equiv., if solid), $LiClO_4$ (43 mg, 0.40 mmol, 2.0 equiv.) and $Ni(acac)_2$ (5.1 mg, 0.020 mmol, 10 mol%). The tube was then evacuated and backfilled with nitrogen gas three times. DIPEA (103 mg, 0.80 mmol, 4.0 equiv.), 0.1 M solution of 4-picoline in DMF (0.4 mL, 20 mol%) and MeCN (3.6 mL) were added under positive nitrogen pressure. The electrodes were positioned so that each was immersed 10 mm into the solution, and the mixture was stirred under constant-current electrolysis at 4 mA for 14 h. Upon completion, the mixture was exposed to air, and the electrodes were rinsed thoroughly with CH_2Cl_2. The mixture was filtered through SiO_2 plug and the solvent was removed under reduced pressure. The residue, which subsequently was purified by column chromatography to afford the corresponding products and *α*:*β*-ratio was determined by ^{1}H NMR.

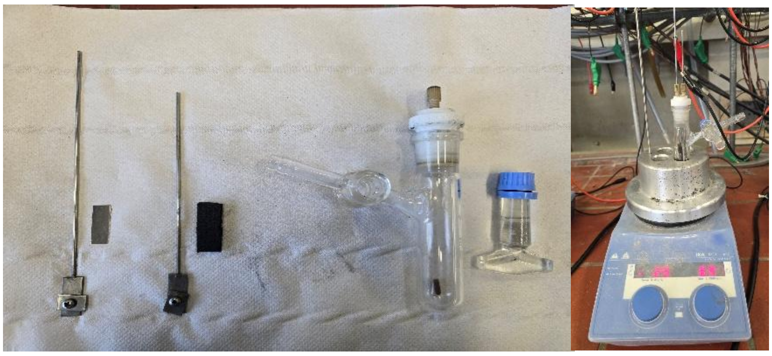

Figure S1. Photograph of the electrolysis setup.

Synthesis of 3a in 1.0 mmol scale reaction:

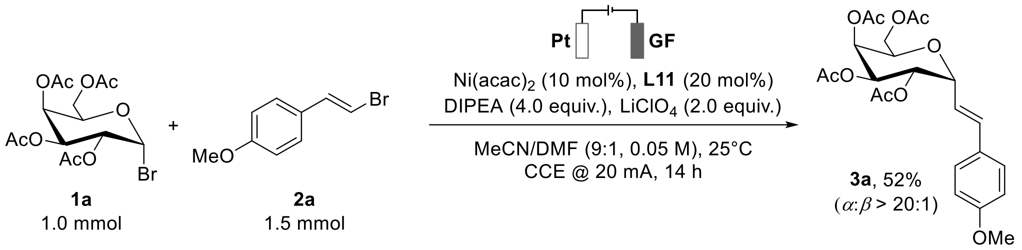

A flame-dried 250 ml Schlenk tube (undivided electrochemical cell) equipped with a stirring bar, platinum electrode (5 × 2.5 × 0.1 cm) and graphite felt electrode (5 × 2.5 × 0.4 cm) was charged with glycosyl bromide **1a** (411 mg, 1.0 mmol, 1.0 equiv.), alkenyl bromide **2a** (320 mg, 1.5 mmol, 1.5 equiv.), $LiClO_4$ (213 mg, 2.0 mmol, 2.0 equiv.) and $Ni(acac)_2$ (25.7 mg, 0.10 mmol, 10 mol%). The tube was then evacuated and backfilled with nitrogen gas three times, DIPEA (517 mg, 4.0 mmol, 4.0 equiv.), 0.1 M solution of 4-picoline in DMF (2 mL, 20 mol%) and MeCN (18 mL) were added under positive nitrogen pressure. The electrodes were positioned so that each was immersed into the solution, and the mixture was stirred under constant-current electrolysis at 20 mA for 14 h. Upon completion, the mixture was exposed to air, and the electrodes were rinsed thoroughly with CH_2Cl_2. The reaction mixture was concentrated under reduced pressure and the residue was chromatographed through silica gel eluting with ethyl acetate/hexane (4:1) to give the desired product **3a** as a colorless syrup (242 mg, 0.52 mmol, 52% yield).

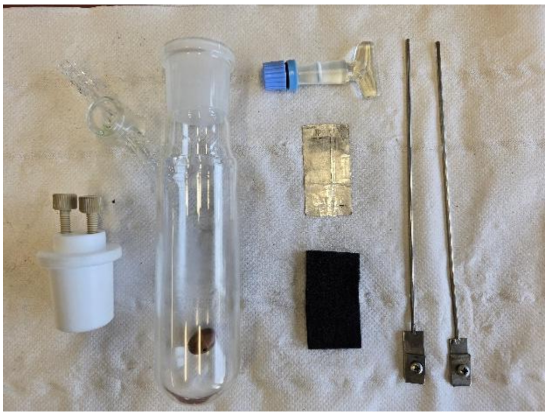

Figure S2. Photograph of the bulk electrolysis setup.

(2*R*,3*S*,4*R*,5*S*,6*R*)-2-(acetoxymethyl)-6-((*E*)-4-methoxystyryl)tetrahydro-2*H*-pyran-3,4,5-triyl triacetate (3a)

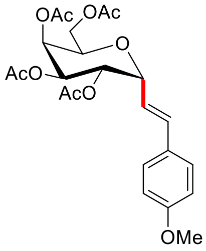

Product **3a** was obtained from galactosyl bromide **1a** (82.2 mg, 0.20 mmol) and alkenyl bromide **2a** (63.9 mg, 0.30 mmol) according to *General Procedure 2*. Column chromatography (SiO_2, hexane/EtOAc 4:1) afforded **3a** (66.0 mg, 71%, α:β > 20:1) as a colorless syrup. α-conformation was confirmed by ^{1}H-NMR coupling constants and 2D NMR analysis. Spectra were in accordance with those reported in the literature.[11]

^{1}H-NMR (400 MHz, $CDCl_3$): δ = 7.36 – 7.31 (m, 2H), 6.90 – 6.85 (m, 2H), 6.70 (dd, *J* = 16.0, 1.7 Hz, 1H), 6.18 (dd, *J* = 16.0, 5.9 Hz, 1H), 5.43 (dd, *J* = 3.4, 1.6 Hz, 1H), 5.37 (dd, *J* = 10.5, 5.9 Hz, 1H), 5.22 (dd, *J* = 10.6, 3.3 Hz, 1H), 4.94 (ddd, *J* = 5.9, 5.9, 1.7 Hz, 1H), 4.23 (td, *J* = 6.4, 1.7 Hz, 1H), 4.18 – 4.07 (m, 2H), 3.81 (s, 3H), 2.15 (s, 3H), 2.04 (s, 6H), 2.01 (s, 3H) ppm.

^{13}C{^{1}H}-NMR (101 MHz, $CDCl_3$): δ = 170.5 (C), 170.3 (C), 170.2 (C), 170.0 (C), 159.9 (C), 135.8 (CH), 128.8 (C), 128.0 (2 x CH), 118.2 (CH), 114.2 (2 x CH), 73.5 (CH), 68.5 (CH), 68.3 (CH), 68.2 (CH), 68.2 (CH), 62.0 (CH_2), 55.4 (CH_3), 20.9 (CH_3), 20.8 (CH_3), 20.8 (CH_3), 20.7 (CH_3) ppm.

IR (ATR): $\tilde{\nu}$ = 2965, 1737, 1607, 1511, 1369, 1251, 1175, 1029, 907, 728, 648 cm^{-1}.

MS (ESI-TOF): *m/z* (relative intensity) 487 (100) [M+Na]$^+$, 951 (25) [2M+Na]$^+$.

HRMS (ESI-TOF): *m/z* [M+Na]$^+$ Calcd. for $C_{23}H_{28}O_{10}Na^+$: 487.1575; Found: 487.1579.

(2*R*,3*S*,4*R*,5*S*,6*R*)-2-(acetoxymethyl)-6-((*E*)-styryl)tetrahydro-2*H*-pyran-3,4,5-triyl triacetate (3b)

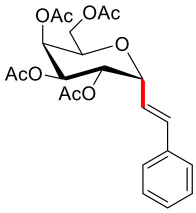

Product **3b** was obtained from galactosyl bromide **1a** (82.2 mg, 0.20 mmol) and alkenyl bromide **2b** (54.9 mg, 0.30 mmol) according to *General Procedure 2*. Column chromatography (SiO_2, hexane/EtOAc 6:1) afforded **3b** (63.3 mg, 73%, α:β > 20:1) as a colorless oil.

^{1}H-NMR (400 MHz, $CDCl_3$): δ = 7.43 – 7.39 (m, 2H), 7.38 – 7.33 (m, 2H), 7.32 – 7.27 (m, 1H), 6.77 (dd, *J* = 16.1, 1.7 Hz, 1H), 6.32 (dd, *J* = 16.1, 5.7 Hz, 1H), 5.44 (dd, *J* = 3.4, 1.7 Hz, 1H), 5.40 (dd, *J* = 10.5, 6.0 Hz, 1H), 5.22 (dd, *J* = 10.5, 3.3 Hz, 1H), 4.97 (ddd, *J* = 5.8, 5.8, 1.8 Hz, 1H), 4.25 (td, *J* = 6.4, 1.7 Hz, 1H), 4.20 – 4.08 (m, 2H), 2.16 (s, 3H), 2.06 (s, 3H), 2.05 (s, 3H), 2.02 (s, 3H) ppm.

^{13}C{^{1}H}-NMR (101 MHz, $CDCl_3$): δ = 170.6 (C), 170.3 (C), 170.2 (C), 170.0 (C), 136.2 (CH), 136.0 (C), 128.8 (2 x CH), 128.5 (CH), 126.7 (2 x CH), 120.7 (CH), 73.3 (CH), 68.5 (CH), 68.5 (CH), 68.2 (2 x CH), 62.0 (CH_2), 20.9 (CH_3), 20.8 (CH_3), 20.8 (CH_3), 20.8 (CH_3) ppm.

IR (ATR): $\tilde{\nu}$ = 2962, 1739, 1368, 1212, 1115, 1044, 972, 909, 735, 696, 590, 462 cm^{-1}.

MS (ESI-TOF): *m/z* (relative intensity) 457 (100) [M+Na]$^+$, 891 (60) [2M+Na]$^+$.

HRMS (ESI-TOF): *m/z* [M+Na]$^+$ Calcd. for $C_{22}H_{26}O_9Na^+$: 457.1469; Found: 457.1474.

(2*R*,3*S*,4*R*,5*S*,6*R*)-2-(acetoxymethyl)-6-((*E*)-4-fluorostyryl)tetrahydro-2*H*-pyran-3,4,5-triyl triacetate (3c)

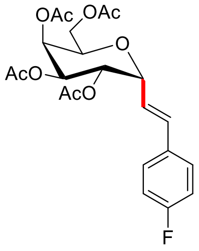

Product **3c** was obtained from galactosyl bromide **1a** (82.2 mg, 0.20 mmol) and alkenyl bromide **2c** (60.3 mg, 0.30 mmol) according to *General Procedure 2*. Column chromatography (SiO_2, hexane/EtOAc 6:1) afforded **3c** (73.7 mg, 81%, α:β > 20:1) as a colorless syrup.

^{1}H-NMR (400 MHz, $CDCl_3$): δ = 7.41 – 7.35 (m, 2H), 7.08 – 7.01 (m, 2H), 6.73 (dd, *J* = 16.1, 1.7 Hz, 1H), 6.23 (dd, *J* = 16.1, 5.6 Hz, 1H), 5.44 (dd, *J* = 3.4, 1.7 Hz, 1H), 5.39 (dd, *J* = 10.5, 5.9 Hz, 1H), 5.21 (dd, *J* = 10.5, 3.3 Hz, 1H), 4.95 (dd, *J* = 5.9, 5.8, 1.8 Hz, 1H), 4.23 (td, *J* = 6.3, 1.7 Hz, 1H), 4.21 – 4.14 (m, 1H), 4.11 (dd, *J* = 11.1, 6.1 Hz, 1H), 2.16 (s, 3H), 2.06 (s, 3H), 2.05 (s, 3H), 2.03 (s, 3H) ppm.

^{13}C{^{1}H}-NMR (101 MHz, $CDCl_3$): δ = 170.6 (C), 170.3 (C), 170.2 (C), 170.0 (C), 162.9 (d, *J* = 248.2 Hz, C), 134.9 (CH), 132.2 (d, *J* = 3.4 Hz, C), 128.4 (d, *J* = 8.1 Hz, 2 x CH), 120.5 (d, *J* = 2.3 Hz, CH), 115.8 (d, *J* = 21.7 Hz, 2 x CH), 73.2 (CH), 68.5 (2 x CH), 68.2 (CH), 68.1 (CH), 62.0 (CH_2), 20.9 (CH_3), 20.8 (CH_3), 20.8 (CH_3), 20.8 (CH_3) ppm.

^{19}F-NMR (377 MHz, $CDCl_3$): -113.02 – -113.09 (m, 1F) ppm.

IR (ATR): $\tilde{\nu}$ = 2973, 1742, 1601, 1509, 1369, 1218, 1160, 1049, 825, 602, 464 cm^{-1}.

MS (ESI-TOF): *m/z* (relative intensity) 475 (100) [M+Na]$^+$, 927 (40) [2M+Na]$^+$.

HRMS (ESI-TOF): *m/z* [M+Na]$^+$ Calcd. for $C_{22}H_{25}FO_9Na^+$: 475.1375; Found: 475.1377.

(2*R*,3*S*,4*R*,5*S*,6*R*)-2-(acetoxymethyl)-6-((*E*)-4-chlorostyryl)tetrahydro-2*H*-pyran-3,4,5-triyl triacetate (3d)

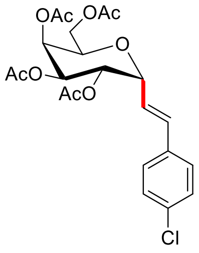

Product **3d** was obtained from galactosyl bromide **1a** (82.2 mg, 0.20 mmol) and alkenyl bromide **2d** (65.2 mg, 0.30 mmol) according to *General Procedure 2*. Column chromatography (SiO_2, hexane/EtOAc 6:1) afforded **3d** (72.1 mg, 77%, α:β > 20:1) as a colorless syrup.

^{1}**H-NMR** (400 MHz, $CDCl_3$): δ = 7.35 – 7.27 (m, 4H), 6.72 (dd, J = 16.1, 1.8 Hz, 1H), 6.28 (dd, J = 16.1, 5.6 Hz, 1H), 5.44 (dd, J = 3.3, 1.7 Hz, 1H), 5.39 (dd, J = 10.4, 5.9 Hz, 1H), 5.19 (dd, J = 10.4, 3.3 Hz, 1H), 4.95 (ddd, J = 5.9, 5.7, 1.8 Hz, 1H), 4.22 (td, J = 6.2, 1.7 Hz, 1H), 4.20 – 4.14 (m, 1H), 4.10 (dd, J = 11.0, 5.9 Hz, 1H), 2.16 (s, 3H), 2.05 (s, 3H), 2.05 (s, 3H), 2.02 (s, 3H) ppm.

^{13}**C{^{1}H}-NMR** (101 MHz, $CDCl_3$): δ = 170.5 (C), 170.3 (C), 170.2 (C), 170.0 (C), 134.8 (CH), 134.5 (C), 134.2 (C), 129.0 (2 x CH), 127.9 (2 x CH), 121.5 (CH), 73.1 (CH), 68.6 (CH), 68.5 (CH), 68.1 (CH), 68.1 (CH), 61.9 (CH_2), 20.9 (CH_3), 20.8 (CH_3), 20.8 (CH_3), 20.8 (CH_3) ppm.

IR (ATR): $\tilde{\nu}$ = 2961, 1740, 1491, 1368, 1212, 1083, 1044, 811, 735, 701, 591 cm^{-1}.

MS (ESI-TOF): *m/z* (relative intensity) 491 (100) $[M+Na]^+$, 959 (40) $[2M+Na]^+$.

HRMS (ESI-TOF): *m/z* $[M+Na]^+$ Calcd. for $C_{22}H_{25}ClO_9Na^+$: 491.1079; Found: 491.1084.

(2*R*,3*S*,4*R*,5*S*,6*R*)-2-(acetoxymethyl)-6-((*E*)-4-(trifluoromethyl)styryl)tetrahydro-2*H*-pyran-3,4,5-triyl triacetate (3e)

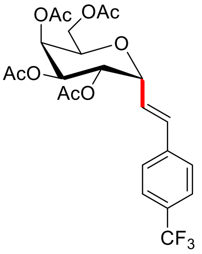

Product **3e** was obtained from galactosyl bromide **1a** (82.2 mg, 0.20 mmol) and alkenyl bromide **2e** (75.3 mg, 0.30 mmol) according to *General Procedure 2*. Column chromatography (SiO_2, hexane/EtOAc 6:1) afforded **3e** (60.9 mg, 60%, α:β > 20:1) as a yellow oil.

^{1}**H-NMR** (400 MHz, $CDCl_3$): δ = 7.59 (d, J = 8.2 Hz, 2H), 7.49 (d, J = 8.2 Hz, 2H), 6.80 (dd, J = 16.1, 1.8 Hz, 1H), 6.40 (dd, J = 16.2, 5.3 Hz, 1H), 5.44 (dd, J = 3.4, 1.6 Hz, 1H), 5.40 (dd, J = 10.3, 5.9 Hz, 1H), 5.19 (dd, J = 10.3, 3.3 Hz, 1H), 4.98 (dd, J = 5.9, 5.6, 1.8 Hz, 1H), 4.25 – 4.18 (m, 2H), 4.10 (dd, J = 10.3, 4.9 Hz, 1H), 2.16 (s, 3H), 2.06 (s, 3H), 2.05 (s, 3H), 2.02 (s, 3H) ppm.

^{13}**C{^{1}H}-NMR** (101 MHz, $CDCl_3$): δ = 170.5 (C), 170.2 (C), 170.1 (C), 169.9 (C), 139.4 (C), 134.4 (CH), 130.2 (q, J = 32.7 Hz, C), 126.9 (2 x CH), 125.8 (q, J = 3.9 Hz, 2 x CH), 124.1 (q, J = 272.1 Hz, C), 123.8 (CH), 72.9 (CH), 68.7 (CH), 68.5 (CH), 68.1 (CH), 68.0 (CH), 61.8 (CH_2), 20.9 (CH_3), 20.8 (CH_3), 20.8 (CH_3), 20.7 (CH_3) ppm.

^{19}**F-NMR** (377 MHz, $CDCl_3$): -62.63 (s, 3F) ppm.

IR (ATR): $\tilde{\nu}$ = 2964, 1741, 1370, 1323, 1213, 1163, 1118, 1065, 735, 599, 471 cm^{-1}.

MS (ESI-TOF): *m/z* (relative intensity) 525 (100) $[M+Na]^+$, 1027 (95) $[2M+Na]^+$.

HRMS (ESI-TOF): *m/z* $[M+Na]^+$ Calcd. for $C_{23}H_{25}F_3O_9Na^+$: 525.1343; Found: 525.1346.

(2*R*,3*S*,4*R*,5*S*,6*R*)-2-(acetoxymethyl)-6-((*E*)-4-(methoxycarbonyl)styryl)tetrahydro-2*H*-pyran-3,4,5-triyl triacetate (3f)

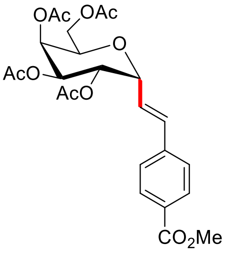

Product **3f** was obtained from galactosyl bromide **1a** (82.2 mg, 0.20 mmol) and alkenyl bromide **2f** (72.3 mg, 0.30 mmol) according to *General Procedure 2*. Column chromatography (SiO_2, hexane/EtOAc 4:1) afforded **3f** (63.4 mg, 64%, α:β > 20:1) as a colorless syrup.

^{1}H-NMR (400 MHz, $CDCl_3$): δ = 8.02 (d, J = 8.3 Hz, 2H), 7.46 (d, J = 8.3 Hz, 2H), 6.80 (dd, J = 16.2, 1.8 Hz, 1H), 6.41 (dd, J = 16.2, 5.3 Hz, 1H), 5.44 (dd, J = 3.4, 1.7 Hz, 1H), 5.41 (dd, J = 10.4, 5.9 Hz, 1H), 5.19 (dd, J = 10.4, 3.3 Hz, 1H), 4.98 (ddd, J = 5.9, 5.6, 1.9 Hz, 1H), 4.26 – 4.17 (m, 2H), 4.11 (dd, J = 11.0, 5.8 Hz, 1H), 3.92 (s, 3H), 2.16 (s, 3H), 2.07 (s, 3H), 2.05 (s, 3H), 2.03 (s, 3H) ppm.

^{13}C{^{1}H}-NMR (101 MHz, $CDCl_3$): δ = 170.5 (C), 170.2 (C), 170.1 (C), 169.9 (C), 166.7 (C), 140.3 (C), 134.8 (CH), 130.1 (2 x CH), 129.8 (C), 126.6 (2 x CH), 123.7 (CH), 72.9 (CH), 68.6 (CH), 68.5 (CH), 68.0 (CH), 68.0 (CH), 61.9 (CH_2), 52.2 (CH_3), 20.9 (CH_3), 20.8 (CH_3), 20.7 (CH_3), 20.7 (CH_3) ppm.

IR (ATR): $\tilde{\nu}$ = 2953, 2848, 1742, 1718, 1607, 1435, 1369, 1212, 1045, 734, 701, 602, 469 cm^{-1}.

MS (ESI-TOF): *m/z* (relative intensity) 515 (100) [M+Na]$^+$, 1007 (75) [2M+Na]$^+$.

HRMS (ESI-TOF): *m/z* [M+Na]$^+$ Calcd. for $C_{24}H_{28}O_{11}Na^+$: 515.1524; Found: 515.1529.

(2*R*,3*S*,4*R*,5*S*,6*R*)-2-(acetoxymethyl)-6-((*E*)-3-methoxystyryl)tetrahydro-2*H*-pyran-3,4,5-triyl triacetate (3g)

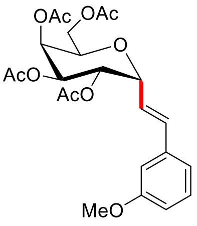

Product **3g** was obtained from galactosyl bromide **1a** (82.2 mg, 0.20 mmol) and alkenyl bromide **2g** (63.9 mg, 0.30 mmol) according to *General Procedure 2*. Column chromatography (SiO_2, hexane/EtOAc 4:1) afforded **3g** (64.1 mg, 69%, α:β > 20:1) as a light yellow syrup.

^{1}H-NMR (400 MHz, $CDCl_3$): δ = 7.27 (dd, J = 8.5, 7.4 Hz, 1H), 7.00 (d, J = 7.6 Hz, 1H), 6.93 (dd, J = 2.5, 1.6 Hz, 1H), 6.84 (dd, J = 8.2, 2.6 Hz, 1H), 6.73 (dd, J = 16.1, 1.7 Hz, 1H), 6.30 (dd, J = 16.1, 5.7 Hz, 1H), 5.44 (dd, J = 3.3, 1.7 Hz, 1H), 5.39 (dd, J = 10.5, 6.0 Hz, 1H), 5.21 (dd, J = 10.5, 3.3 Hz, 1H), 4.96 (ddd, J = 5.9, 5.9, 1.8 Hz, 1H), 4.24 (td, J = 6.4, 1.8 Hz, 1H), 4.13 (m, 2H), 3.83 (s, 3H), 2.16 (s, 3H), 2.05 (s, 3H), 2.05 (s, 3H), 2.02 (s, 3H) ppm.

^{13}C{^{1}H}-NMR (101 MHz, $CDCl_3$): δ = 170.5 (C), 170.3 (C), 170.2 (C), 170.0 (C), 160.0 (C), 137.4 (C), 136.1 (CH), 129.8 (CH), 121.1 (CH), 119.4 (CH), 113.9 (CH), 112.2 (CH), 73.2 (CH), 68.5 (CH), 68.5 (CH), 68.2 (2 x CH), 62.0 (CH_2), 55.4 (CH_3), 20.9 (CH_3), 20.8 (CH_3), 20.8 (CH_3), 20.8 (CH_3) ppm.

IR (ATR): $\tilde{\nu}$ = 2950, 2838, 1744, 1580, 1434, 1370, 1221, 1081, 1046, 910, 731 cm^{-1}.

MS (ESI-TOF): *m/z* (relative intensity) 487 (100) $[M+Na]^+$, 951 (25) $[2M+Na]^+$.

HRMS (ESI-TOF): *m/z* $[M+Na]^+$ Calcd. for $C_{23}H_{28}O_{10}Na^+$: 487.1575; Found: 487.1574.

(2*R*,3*S*,4*R*,5*S*,6*R*)-2-(acetoxymethyl)-6-((*E*)-2-(6-methoxypyridin-3-yl)vinyl)tetrahydro-2*H*-pyran-3,4,5-triyl triacetate (3h)

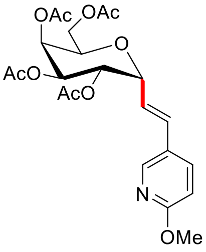

Product **3h** was obtained from galactosyl bromide **1a** (82.2 mg, 0.20 mmol) and alkenyl bromide **2h** (63.9 mg, 0.30 mmol) according to *General Procedure 2*. Column chromatography (SiO_2, hexane/EtOAc 4:1) afforded **3h** (62.4 mg, 67%, $\alpha:\beta$ > 20:1) as a yellow oil.

^{1}H-NMR (400 MHz, $CDCl_3$): δ = 8.09 (d, *J* = 2.5 Hz, 1H), 7.69 (dd, *J* = 8.7, 2.5 Hz, 1H), 6.73 (d, *J* = 8.7 Hz, 1H), 6.68 (dd, *J* = 16.1, 1.7 Hz, 1H), 6.18 (dd, *J* = 16.1, 5.6 Hz, 1H), 5.42 (dd, *J* = 3.3, 1.7 Hz, 1H), 5.37 (dd, *J* = 10.4, 5.9 Hz, 1H), 5.18 (dd, *J* = 10.4, 3.3 Hz, 1H), 4.94 (td, *J* = 5.7, 1.8 Hz, 1H), 4.25 – 4.05 (m, 3H), 3.93 (s, 3H), 2.14 (s, 3H), 2.04 (s, 6H), 2.01 (s, 3H) ppm.

^{13}C{^{1}H}-NMR (101 MHz, $CDCl_3$): δ = 170.4 (C), 170.1 (C), 170.0 (C), 169.8 (C), 164.1 (C), 146.1 (C), 135.4 (CH), 132.1 (CH), 125.1 (C), 119.8 (CH), 111.1 (CH), 73.0 (CH), 68.4 (CH), 68.3 (CH), 68.0 (CH), 68.0 (CH), 61.9 (CH_2), 53.5 (CH_3), 20.8 (CH_3), 20.7 (CH_3), 20.6 (CH_3), 20.6 (CH_3) ppm.

IR (ATR): $\tilde{\nu}$ = 3015, 2983, 2951, 2850, 1741, 1601, 1494, 1369, 1215, 1047, 909, 728 cm^{-1}.

MS (ESI-TOF): *m/z* (relative intensity) 488 (100) $[M+Na]^+$, 953 (20) $[2M+Na]^+$.

HRMS (ESI-TOF): *m/z* $[M+Na]^+$ Calcd. for $C_{22}H_{27}NO_{10}Na^+$: 488.1527; Found: 488.1527.

(2*R*,3*S*,4*R*,5*S*,6*R*)-2-(acetoxymethyl)-6-((*E*)-2-(2-isopropoxypyrimidin-5-yl)vinyl)tetrahydro-2*H*-pyran-3,4,5-triyl triacetate (3i)

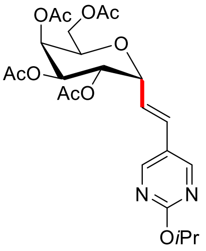

Product **3i** was obtained from galactosyl bromide **1a** (82.2 mg, 0.20 mmol) and alkenyl bromide **2i** (72.6 mg, 0.30 mmol) according to *General Procedure 2*. Column chromatography (SiO_2, hexane/EtOAc 4:1) afforded **3i** (61.1 mg, 62%, $\alpha:\beta$ > 20:1) as a yellow oil.

^{1}H-NMR (300 MHz, $CDCl_3$): δ = 8.52 (s, 2H), 6.63 (dd, *J* = 16.3, 1.7 Hz, 1H), 6.27 (dd, *J* = 16.3, 5.3 Hz, 1H), 5.45 – 5.41 (m, 1H), 5.38 (dd, *J* = 10.2, 5.8 Hz, 1H), 5.32 – 5.22 (m, 1H), 5.16 (dd, *J* = 10.2, 3.3 Hz, 1H), 4.94 (td, *J* = 5.6, 1.8 Hz, 1H), 4.25 – 4.15 (m, 2H), 4.14 – 4.04 (m, 1H), 2.15 (s, 3H), 2.06 (s, 3H), 2.04 (s, 3H), 2.02 (s, 3H), 1.40 (s, 3H), 1.38 (s, 3H) ppm.

^{13}C{^{1}H}-NMR (75 MHz, $CDCl_3$): δ = 170.47 (C), 170.13 (C), 170.03 (C), 169.82 (C), 164.5 (C), 157.0 (2 x CH), 128.6 (CH), 123.1 (C), 121.9 (CH), 72.7 (CH), 70.7 (CH), 68.7 (CH), 68.3 (CH), 68.0 (CH), 67.8 (CH), 61.7 (CH_2), 21.8 (2 x CH_3), 20.8 (CH_3), 20.7 (CH_3), 20.7 (CH_3), 20.6 (CH_3) ppm.

IR (ATR): $\tilde{\nu}$ = 2979, 2960, 2925, 2855, 1743, 1594, 1438, 1215, 1048, 730 cm^{-1}.

MS (ESI-TOF): *m/z* (relative intensity) 517 (100) $[M+Na]^+$, 1011 (10) $[2M+Na]^+$.

HRMS (ESI-TOF): *m/z* $[M+Na]^+$ Calcd. for $C_{23}H_{30}N_2O_{10}Na^+$: 517.1793; Found: 517.1796.

(2*R*,3*S*,4*R*,5*S*,6*R*)-2-((*E*)-2-(1*H*-indol-4-yl)vinyl)-6-(acetoxymethyl)tetrahydro-2*H*-pyran-3,4,5-triyl triacetate (3j)

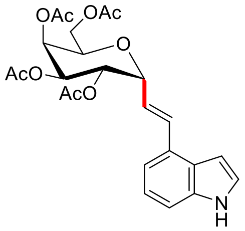

Product **3j** was obtained from galactosyl bromide **1a** (82.2 mg, 0.20 mmol) and alkenyl bromide **2j** (66.6 mg, 0.30 mmol) according to *General Procedure 2*. Column chromatography (SiO_2, hexane/EtOAc 2:1) afforded **3j** (45.0 mg, 48%, α:β > 20:1) as a yellow oil.

^{1}H-NMR (300 MHz, $CDCl_3$): δ = 8.42 (s, 1H), 7.39 – 7.33 (m, 1H), 7.30 – 7.15 (m, 4H), 6.72 (m, 1H), 6.49 (dd, *J* = 16.1, 5.8 Hz, 1H), 5.51 – 5.39 (m, 2H), 5.28 (dd, *J* = 10.4, 3.5 Hz, 1H), 5.07 (td, *J* = 6.0, 1.8 Hz, 1H), 4.33 (td, *J* = 6.3, 1.6 Hz, 1H), 4.24 – 4.06 (m, 2H), 2.17 (s, 3H), 2.06 (s, 3H), 2.05 (s, 3H), 2.03 (s, 3H) ppm.

^{13}C{^{1}H}-NMR (75 MHz, $CDCl_3$): δ = 170.5 (C), 170.3 (C), 170.2 (C), 170.0 (C), 136.2 (C), 134.7 (CH), 128.3 (C), 126.2 (C), 124.8 (CH), 122.0 (CH), 121.0 (CH), 117.8 (CH), 111.2 (CH), 100.9 (CH), 73.6 (CH), 68.5 (CH), 68.3 (2 x CH), 68.2 (CH), 62.2 (CH_2), 20.9 (CH_3), 20.7 (CH_3), 20.7 (CH_3), 20.7 (CH_3) ppm.

IR (ATR): $\tilde{\nu}$ = 3407, 2979, 2958, 2924, 2857, 1738, 1369, 1214, 1045, 909, 729 cm^{-1}.

MS (ESI-TOF): *m/z* (relative intensity) 496 (100) $[M+Na]^+$.

HRMS (ESI-TOF): *m/z* $[M+Na]^+$ Calcd. for $C_{24}H_{27}N_2O_9Na^+$: 496.1578; Found: 496.1578.

(2*R*,3*R*,4*R*,5*R*,6*R*)-2-(acetoxymethyl)-6-((*E*)-4-methoxystyryl)tetrahydro-2*H*-pyran-3,4,5-triyl triacetate (3k)

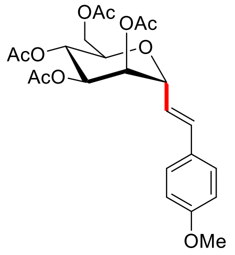

Product **3k** was obtained from mannosyl bromide **1b** (82.2 mg, 0.20 mmol) and alkenyl bromide **2a** (63.9 mg, 0.30 mmol) according to *General Procedure 2*. Column chromatography (SiO_2, hexane/EtOAc 4:1) afforded **3k** (27.2 mg, 29%, α:β > 20:1) as a yellow oil.

^{1}H-NMR (400 MHz, $CDCl_3$): δ = 7.39 – 7.33 (m, 2H), 6.91 – 6.86 (m, 2H), 6.73 (dd, *J* = 16.4, 1.9, 1H), 6.06 (dd, *J*=16.4, 4.8, 1H), 5.52 (t, *J* = 2.8, 1H), 5.32 (dd, *J* = 9.2, 9.2, 1H), 5.25 (dd, *J* = 9.5, 3.2, 1H), 4.70 (dt, *J* = 4.7, 2.3, 1H), 4.33 (dd, *J* = 12.2, 5.6, 1H), 4.14 (dd, *J* = 12.2, 2.7, 1H), 4.01 (ddd, *J* = 8.6, 5.6, 2.6, 1H), 3.82 (s, 3H), 2.18 (s, 3H), 2.12 (s, 3H), 2.04 (s, 3H), 2.03 (s, 3H) ppm.

^{13}C{^{1}H}-NMR (101 MHz, $CDCl_3$): δ = 170.8 (C), 170.5 (C), 170.3 (C), 169.8 (C), 160.0 (C), 134.7 (CH), 128.5 (C), 128.1 (2 x CH), 120.2 (CH), 114.2 (2 x CH), 75.8 (CH), 71.0 (CH), 70.6 (CH), 69.6 (CH), 66.8 (CH), 62.8 (CH_2), 55.4 (CH_3), 21.1 (CH_3), 20.9 (CH_3), 20.8 (2 x CH_3) ppm.

IR (ATR): $\tilde{\nu}$ = 2954, 2922, 2852, 1738, 1606, 1512, 1367, 1212, 1031, 972, 821, 735, 600 cm^{-1}.

MS (ESI-TOF): *m/z* (relative intensity) 487 (100) $[M+Na]^+$, 951 (40) $[2M+Na]^+$.

HRMS (ESI-TOF): *m/z* $[M+Na]^+$ Calcd. for $C_{23}H_{28}O_{10}Na^+$: 487.1575; Found: 487.1578.

(2*R*,3*R*,4*R*,5*S*,6*R*)-2-(acetoxymethyl)-6-((*E*)-4-methoxystyryl)tetrahydro-2*H*-pyran-3,4,5-triyl triacetate (3l)

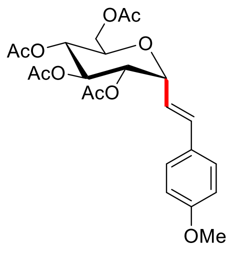

Product **3l** was obtained from glycosyl bromide **1c** (82.2 mg, 0.20 mmol) and alkenyl bromide **2a** (63.9 mg, 0.30 mmol) according to *General Procedure 2*. Column chromatography (SiO_2, hexane/EtOAc 4:1) afforded **3l** (68.7 mg, 74%, α:β = 5:1) as a colorless syrup.

^{1}H-NMR (400 MHz, $CDCl_3$): δ = 7.41 – 7.34 (m, 2H), 6.93 – 6.87 (m, 2H), 6.71 (dd, *J* = 16.0, 1.5, 1H), 6.24 (dd, *J* = 16.0, 6.5, 1H), 5.41 (dd, *J* = 10.1, 9.2, 1H), 5.13 (dd, *J* = 10.1, 6.2, 1H), 5.08 (dd, *J* = 10.0, 9.1, 1H), 4.89 (m, 1H), 4.24 (dd, *J* = 12.2, 4.7, 1H), 4.10 (dd, *J* = 12.2, 2.4, 1H), 4.03 (ddd, *J* = 10.1, 4.7, 2.4, 1H), 3.83 (s, 3H), 2.10 (s, 3H), 2.04 (s, 3H), 2.03 (s, 3H), 2.02 (s, 3H) ppm.

^{13}C{^{1}H}-NMR (101 MHz, $CDCl_3$): δ = 170.8 (C), 170.4 (C), 169.9 (C), 169.7 (C), 160.1 (C), 136.9 (CH), 128.7 (C), 128.2 (2 x CH), 118.2 (CH), 114.2 (2 x CH), 73.5 (CH), 71.0 (CH), 70.7 (CH), 69.3 (CH), 69.2 (CH), 62.5 (CH_2), 55.5 (CH_3), 20.9 (CH_3), 20.9 (CH_3), 20.8 (CH_3), 20.7 (CH_3) ppm.

IR (ATR): $\tilde{\nu}$ = 2956, 2924, 2852, 1741, 1606, 1512, 1366, 1213, 1029, 734, 600 cm^{-1}.

MS (ESI-TOF): *m/z* (relative intensity) 487 (100) [M+Na]$^+$, 951 (70) [2M+Na]$^+$.

HRMS (ESI-TOF): *m/z* [M+Na]$^+$ Calcd. for $C_{23}H_{28}O_{10}Na^+$: 487.1575; Found: 487.1574.

(2*S*,3*S*,4*R*,5*R*,6*R*)-2-(*E*)-4-methoxystyryl)-6-methyltetrahydro-2*H*-pyran-3,4,5-triyl triacetate (3m)

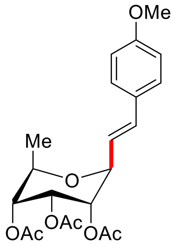

Product **3m** was obtained from rhamnosyl bromide **1d** (70.6 mg, 0.20 mmol) and alkenyl bromide **2a** (63.9 mg, 0.30 mmol) according to *General Procedure 2*. Column chromatography (SiO_2, hexane/EtOAc 4:1) afforded **3m** (54.6 mg, 67%, α:β > 20:1) as a colorless syrup.

^{1}H-NMR (400 MHz, $CDCl_3$): δ = 7.38 – 7.32 (m, 2H), 6.90 – 6.85 (m, 2H), 6.71 (dd, *J* = 16.4, 1.9 Hz, 1H), 6.10 (dd, *J* = 16.4, 4.9 Hz, 1H), 5.53 (dd, *J* = 3.2, 2.4 Hz, 1H), 5.22 (dd, *J* = 9.7, 3.3 Hz, 1H), 5.13 (dd, *J* = 9.7, 9.0 Hz, 1H), 4.61 (dt, *J* = 4.6, 2.1 Hz, 1H), 3.92 – 3.85 (m, 1H), 3.82 (s, 3H), 2.17 (s, 3H), 2.04 (s, 3H), 2.02 (s, 3H), 1.26 (d, *J* = 6.2 Hz, 3H) ppm.

^{13}C{^{1}H}-NMR (101 MHz, $CDCl_3$): δ = 170.4 (C), 170.3 (C), 169.9 (C), 159.7 (C), 134.0 (CH), 128.5 (C), 127.9 (2 x CH), 120.9 (CH), 114.0 (2 x CH), 75.6 (CH), 71.4 (CH), 70.8 (CH), 69.6 (CH), 68.7 (CH), 55.3 (CH_3), 21.0 (CH_3), 20.8 (CH_3), 20.7 (CH_3), 17.7 (CH_3) ppm.

IR (ATR): $\tilde{\nu}$ = 2954, 1790, 1773, 1602, 1545, 1309, 1255, 1188, 965, 835, 714 cm^{-1}.

MS (ESI-TOF): *m/z* (relative intensity) 429 (100) [M+Na]$^+$, 835 (25) [2M+Na]$^+$.

HRMS (ESI-TOF): *m/z* [M+Na]$^+$ Calcd. for $C_{21}H_{26}O_8Na^+$: 429.1519; Found: 429.1520.

(2*S*,3*S*,4*R*,5*R*,6*R*)-2-(*E*)-4-methoxystyryl)-6-methyltetrahydro-2*H*-pyran-3,4,5-triyl tribenzoate (3n)

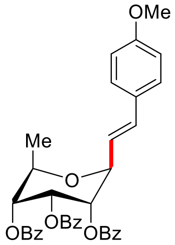

Product **3n** was obtained from rhamnosyl bromide **1e** (107.9 mg, 0.20 mmol) and alkenyl bromide **2a** (63.9 mg, 0.30 mmol) according to *General Procedure 2*. Column chromatography (SiO_2, hexane/EtOAc 4:1) afforded **3n** (51.0 mg, 43%, α:β > 20:1) as a colorless syrup.

^{1}H-NMR (400 MHz, $CDCl_3$): δ = 8.14 – 8.11 (m, 2H), 7.99 – 7.96 (m, 2H), 7.87 – 7.84 (m, 2H), 7.63 – 7.59 (m, 1H), 7.52 – 7.45 (m, 6H), 7.38 (dd, *J* = 8.4, 7.2 Hz, 2H), 7.31 – 7.27 (m, 2H), 6.95 – 6.91 (m, 2H), 6.88 (dd, *J* = 16.4, 1.9 Hz, 1H), 6.31 (ddd, *J* = 16.4, 4.8, 1.1 Hz, 1H), 5.95 (s, 1H), 5.76 – 5.72 (m, 2H), 4.92 (dt, *J* = 4.6, 2.1 Hz, 1H), 4.24 – 4.16 (m, 1H), 3.85 (s, 3H), 1.41 (d, *J* = 6.2 Hz, 3H).

^{13}**C{^{1}H}-NMR** (101 MHz, CDCl$_3$): δ = 165.8 (C), 165.8 (C), 165.7 (C),159.8 (C), 134.1 (CH), 133.3 (CH), 133.2 (CH), 133.1 (CH), 129.9 (2 x CH), 129.7 (2 x CH), 129.6 (2 x CH), 129.6 (CH), 129.3 (C), 129.1 (CH), 128.6 (C), 128.5 (2 x CH), 128.4 (2 x CH) 128.3 (2 x CH), 128.0 (2 x CH), 121.0 (CH), 114.1 (2 x CH), 75.9 (CH), 72.2 (CH), 72.1 (CH), 70.5 (CH), 68.9 (CH), 55.3 (CH$_3$), 18.0 (CH$_3$) ppm.

IR (ATR): $\tilde{\nu}$ = 2845, 1756, 1723, 1657, 1509, 1373, 1112, 1180, 912, 812, 745 cm^{-1}.

MS (ESI-TOF): *m/z* (relative intensity) 615 (100) [M+Na]$^+$, 1207 (75) [2M+Na]$^+$.

HRMS (ESI-TOF): *m/z* [M+Na]$^+$ Calcd. for $C_{36}H_{32}O_8Na^+$: 615.1989; Found: 615.1989.

(2*R*,3*S*,4*R*,5*S*,6*R*)-2-(benzoyloxy)methyl)-6-(*E*)-4-methoxystyryl)tetrahydro-2*H*-pyran-3,4,5-triyl tribenzoate (3o)

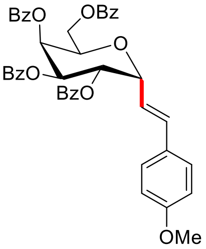

Product **3o** was obtained from galactosyl bromide **1f** (131.9 mg, 0.20 mmol) and alkenyl bromide **2a** (63.9 mg, 0.30 mmol) according to *General Procedure 2*. Column chromatography (SiO$_2$, hexane/EtOAc 4:1) afforded **3o** (105.4 mg, 74%, α:β > 20:1) as a colorless syrup.

^{1}**H-NMR** (400 MHz, CDCl$_3$): δ 8.10 – 8.06 (m, 2H), 7.98 – 7.89 (m, 7H), 7.52 – 7.48 (m, 2H), 7.48 – 7.41 (m, 4H), 7.38 – 7.31 (m, 8H), 6.89 – 6.86 (m, 2H), 6.44 (dd, J = 16.1, 5.6 Hz, 1H), 6.07 (dd, J = 10.0, 9.2 Hz, 1H), 5.74 – 5.67 (m, 1H), 5.60 (dd, J = 10.0, 6.0 Hz, 1H), 5.22 (td, J = 5.8, 1.8 Hz, 1H), 4.64 – 4.59 (m, 1H), 4.55 – 4.48 (m, 2H), 3.83 (s, 3H).

^{13}**C{^{1}H}-NMR** (101 MHz, CDCl$_3$): δ 166.1 (C), 165.9 (C), 165.4 (C), 165.2 (C), 159.8 (C), 136.1 (CH), 133.4 (CH), 133.3 (CH), 133.2 (CH), 133.1 (CH), 129.8 (2 x CH), 129.8 (2 x CH), 129.7 (3 x CH), 129.7 (2 x CH), 129.0 (C), 128.9 (C), 128.8 (C), 128.7 (C), 128.4 (2 x CH), 128.3 (4 x CH), 128.3 (2 x CH), 128.0 (2 x CH), 118.3 (CH), 114.0 (2 x CH), 73.2 (CH), 71.6 (CH), 71.1 (CH), 69.9 (CH), 69.9 (CH), 63.4 (CH$_2$), 55.3 (CH$_3$).

IR (ATR): $\tilde{\nu}$ = 2978, 2821, 1766, 1753, 1689, 1678, 1356, 1132, 1107, 857, 844, 741 cm^{-1}.

MS (ESI-TOF): *m/z* (relative intensity) 735 (100) [M+Na]$^+$, 1447 (25) [2M+Na]$^+$.

HRMS (ESI-TOF): *m/z* [M+Na]$^+$ Calcd. for $C_{43}H_{36}O_{10}Na^+$: 735.2210; Found: 735.2212.

(2*R*,3*S*,4*R*,5*R*,6*R*)-2-((*E*)-4-methoxystyryl)-6-((pivaloyloxy)methyl)tetrahydro-2*H*-pyran-3,4,5-triyl tris(2,2-dimethylpropanoate) (3p)

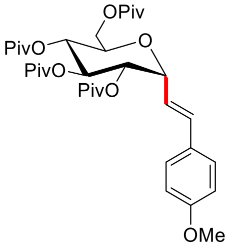

Product **3p** was obtained from glycosyl bromide **1g** (115.9 mg, 0.20 mmol) and alkenyl bromide **2a** (63.9 mg, 0.30 mmol) according to *General Procedure 2*. Column chromatography (SiO_2, hexane/EtOAc 4:1) afforded **3p** (93.7 mg, 74%, $\alpha:\beta$ = 5:1) as a colorless solid.

^{1}H-NMR (400 MHz, $CDCl_3$): δ = 7.37 – 7.31 (m, 2H), 6.91 – 6.86 (m, 2H), 6.69 (dd, *J* = 16.0, 1.4, 1H), 6.28 (dd, *J* = 15.9, 6.9, 1H), 5.51 (dd, *J* = 10.1, 9.4, 1H), 5.14 (dd, *J* = 10.1, 9.4, 1H), 5.09 (dd, *J* = 10.1, 6.4, 1H), 4.88 (td, *J* = 6.7, 1.4, 1H), 4.14 (dd, *J* = 12.2, 2.1, 1H), 4.07 (dd, *J* = 12.2, 5.2, 1H), 4.00 (ddd, *J* = 10.1, 5.1, 1.9, 1H), 3.82 (s, 3H), 1.21 (s, 9H), 1.16 (s, 9H), 1.14 (s, 9H), 1.10 (s, 9H) ppm.

^{13}C{^{1}H}-NMR (101 MHz, $CDCl_3$): δ = 178.2 (C), 177.4 (C), 177.3 (C), 176.7 (C), 160.0 (C), 137.0 (CH), 128.7 (C), 128.0 (2 x CH), 118.6 (CH), 114.2 (2 x CH), 73.5 (CH), 71.1 (CH), 70.5 (CH), 69.7 (CH), 68.5 (CH), 62.4 (CH_2), 55.4 (CH_3), 39.0 (C), 38.9 (2 x C), 38.8 (C), 27.3 (3 x CH_3), 27.2 (3 x CH_3), 27.2 (3 x CH_3), 27.2 (3 x CH_3) ppm.

IR (ATR): $\tilde{\nu}$ = 2971, 2935, 2873, 1733, 1607, 1512, 1479, 1279, 1253, 1133, 1033, 968, 762 cm^{-1}.

MS (ESI-TOF): *m/z* (relative intensity) 655 (100) $[M+Na]^+$, 1287 (40) $[2M+Na]^+$.

HRMS (ESI-TOF): *m/z* $[M+Na]^+$ Calcd. for $C_{35}H_{52}O_{10}Na^+$: 655.3453; Found: 655.3451.

(2*R*,3*R*,4*S*,5*R*,6*R*)-2-(acetoxymethyl)-6-(2*R*,3*R*,4*S*,5*S*,6*R*)-4,5-diacetoxy-2-(acetoxymethyl)-6-(*E*)-4-methoxystyryl)tetrahydro-2*H*-pyran-3yl)oxy)tetrahydro-2*H*-pyran-3,4,5-triyl triacetate (3q)

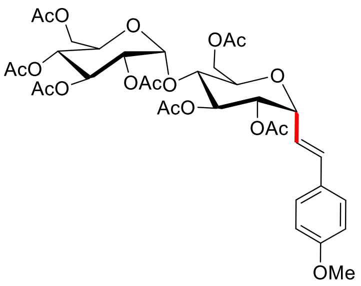

Product **3q** was obtained from maltosyl bromide **1h** (139.9 mg, 0.20 mmol) and alkenyl bromide **2a** (63.9 mg, 0.30 mmol) according to *General Procedure 2*. Column chromatography (SiO_2, hexane/EtOAc 2:1) afforded **3q** (104.0 mg, 59%, $\alpha:\beta$ = 10:1) as a colorless syrup.

^{1}H-NMR (400 MHz, $CDCl_3$): δ = 7.40 – 7.36 (m, 2H), 6.93 – 6.87 (m, 2H), 6.70 (dd, *J* = 16.1, 1.6 Hz, 1H), 6.19 (dd, *J* = 16.0, 6.2 Hz, 1H), 5.39 – 5.36 (m, 2H), 5.35 – 5.31 (m, 1H), 5.09 – 4.99 (m, 2H), 4.88 (dd, *J* = 10.6, 3.9 Hz, 1H), 4.77 (td, *J* = 5.9, 1.6 Hz, 1H), 4.41 (dd, *J* = 12.0, 2.9 Hz, 1H), 4.24 (ddd, *J* = 12.3, 4.5, 1.5 Hz, 2H), 4.09 – 4.01 (m, 3H), 3.90 (dd, *J* = 8.8, 7.5 Hz, 1H), 3.82 (s, 3H), 2.14 (s, 3H), 2.09 (s, 3H), 2.06 (s, 3H), 2.04 (s, 3H), 2.03 (s, 3H), 2.01 (s, 3H), 1.99 (s, 3H) ppm.

^{13}C{^{1}H}-NMR (101 MHz, $CDCl_3$): δ = 170.5 (C), 170.5 (C), 170.5 (C), 170.0 (C), 169.8 (C), 169.8 (C), 169.4 (C), 159.8 (C), 135.7 (CH), 128.7 (C), 128.0 (2 x CH), 118.7 (CH), 114.0 (2 x CH), 96.0 (CH), 73.8 (CH), 72.7 (CH), 72.4 (CH), 70.7 (CH), 70.1 (CH), 70.0 (CH), 69.4 (CH), 68.4 (CH), 68.0 (CH), 63.1 (CH_2), 61.5 (CH_2), 55.3 (CH_3), 20.9 (CH_3), 20.8 (CH_3), 20.7 (CH_3), 20.6 (CH_3), 20.6 (CH_3), 20.5 (CH_3), 20.5 (CH_3) ppm.

IR (ATR): $\tilde{\nu}$ = 2934, 1745, 1613, 1507, 1469, 1321, 1142, 1020, 923, 830, 712 cm^{-1}.

MS (ESI-TOF): *m/z* (relative intensity) 775 (100) $[M+Na]^+$.

HRMS (ESI-TOF): *m/z* $[M+Na]^+$ Calcd. for $C_{35}H_{44}O_{18}Na^+$: 775.2420; Found: 775.2416.

(2*R*,3*R*,4*S*,5*R*,6*S*)-2-(acetoxymethyl)-6-(2*R*,3*R*,4*S*,5*S*,6*R*)-4,5-diacetoxy-2-(acetoxymethyl)-6-(*E*)-4-methoxystyryl)tetrahydro-2*H*-pyran-3-yl)oxy)tetrahydro-2*H*-pyran-3,4,5-triyl triacetate (3r)

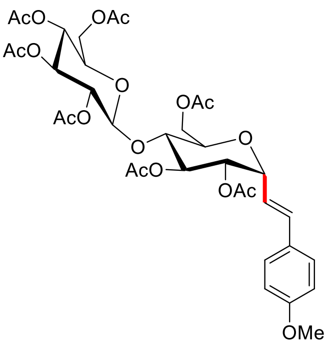

Product **3r** was obtained from cellobiosyl bromide **1k** (139.9 mg, 0.20 mmol) and alkenyl bromide **2a** (63.9 mg, 0.30 mmol) according to *General Procedure 2*. Column chromatography (SiO_2, hexane/EtOAc 2:1) afforded **3r** (90.2 mg, 60%, α:β = 6:1) as a colorless syrup.

^{1}H-NMR (400 MHz, $CDCl_3$): δ = 7.39 – 7.34 (m, 2H), 6.91 – 6.87 (m, 2H), 6.68 (dd, *J* = 16.0, 1.3 Hz, 1H), 6.23 (dd, *J* = 15.9, 6.6 Hz, 1H), 5.39 (dd, *J* = 9.8, 8.6 Hz, 1H), 5.15 – 5.04 (m, 3H), 4.93 (dd, *J* = 7.6, 1.8 Hz, 1H), 4.78 (td, *J* = 6.4, 1.4 Hz, 1H), 4.53 (d, *J* = 8.0 Hz, 1H), 4.45 (dd, *J* = 11.9, 2.2 Hz, 1H), 4.37 (dd, *J* = 12.5, 4.4 Hz, 1H), 4.10 (dd, *J* = 11.9, 4.8 Hz, 1H), 4.05 (dd, *J* = 12.4, 2.2 Hz, 1H), 3.90 (ddd, *J* = 10.0, 4.9, 2.2 Hz, 1H), 3.82 (s, 3H), 3.72 (dd, *J* = 9.7, 8.6 Hz, 1H), 3.68 – 3.64 (m, 1H), 2.13 (s, 3H), 2.08 (s, 3H), 2.05 (s, 3H), 2.01 (s, 3H), 2.01 (s, 6H), 1.97 (s, 3H) ppm.

^{13}C{^{1}H}-NMR (101 MHz, $CDCl_3$): δ = 170.4 (C), 170.3 (C), 170.2 (C), 170.0 (C), 169.7 (C), 169.2 (C), 169.0 (C), 159.9 (C), 136.5 (CH), 128.6 (C), 128.0 (2 x CH), 128.0 (CH), 118.4 (CH), 114.0 (2 x CH), 113.9 (CH), 100.9 (CH), 73.2 (CH), 73.0 (CH), 71.9 (CH), 71.6 (CH), 70.6 (CH), 70.2 (CH), 67.7 (CH), 62.2 (CH_2), 61.5 (CH_2), 55.3 (CH_3), 20.8 (CH_3), 20.7 (CH_3), 20.6 (CH_3), 20.5 (CH_3), 20.5 (3 x CH_3) ppm.

IR (ATR): $\tilde{\nu}$ = 2971, 1775, 1743, 1688, 1600, 1456, 1143, 1121, 950, 875, 821 cm^{-1}.

MS (ESI-TOF): *m/z* (relative intensity) 775 (100) $[M+Na]^+$, 1527 (25) $[2M+Na]^+$.

HRMS (ESI-TOF): *m/z* $[M+Na]^+$ Calcd. for $C_{35}H_{44}O_{18}Na^+$: 775.2420; Found: 775.2416.

(2*R*,3*S*,4*S*,5*R*,6*R*)-2-(acetoxymethyl)-6-(2*R*,3*R*,4*S*,5*S*,6*R*)-4,5-diacetoxy-2-(acetoxymethyl)-6-(*E*)-4-methoxystyryl)tetrahydro-2*H*-pyran-3-yl)oxy)tetrahydro-2*H*-pyran-3,4,5-triyl triacetate (3s)

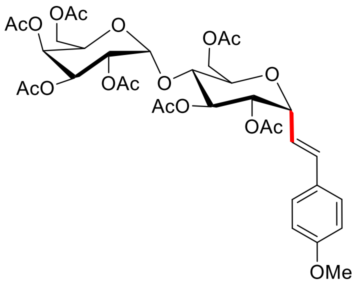

Product **3s** was obtained from lactosyl bromide **1i** (139.9 mg, 0.20 mmol) and alkenyl bromide **2a** (63.9 mg, 0.30 mmol) according to *General Procedure 2*. Column chromatography (SiO_2, hexane/EtOAc 2:1) afforded **3s** (94.7 mg, 63%, $\alpha:\beta$ = 6:1) as a colorless syrup.

^{1}H-NMR (400 MHz, $CDCl_3$): δ = 7.31 (d, J = 8.7 Hz, 2H), 6.90 – 6.79 (m, 2H), 6.65 (d, 1H), 6.16 (dd, J = 16.0, 6.6 Hz, 1H), 5.33 (dd, J = 9.9, 8.5 Hz, 1H), 5.17 – 4.92 (m, 4H), 4.88 – 4.80 (m, 1H), 4.46 (d, J = 7.9 Hz, 1H), 4.42 – 4.28 (m, 2H), 4.04 – 3.93 (m, 2H), 3.84 (ddd, J = 9.7, 4.8, 2.1 Hz, 1H), 3.76 (s, 3H), 3.68 – 3.57 (m, 2H), 2.06 (s, 3H), 2.02 (s, 3H), 1.99 (s, 3H), 1.95 (s, 3H), 1.94 (s, 6H), 1.91 (s, 3H).

^{13}C{^{1}H}-NMR (101 MHz, $CDCl_3$): δ = 170.4 (C), 170.3 (C), 170.1 (C), 170.0 (C), 170.0 (C), 169.6 (C), 169.0 (C), 159.9 (C), 136.4 (CH), 128.6 (C), 128.0 (2 x CH), 118.5 (CH), 114.0 (2 x CH), 113.9 (CH), 101.2 (CH), 73.2 (CH), 71.0 (CH), 70.7 (CH), 70.6 (CH), 70.5 (CH), 70.2 (CH), 69.0 (CH), 66.6 (CH), 62.4 (CH_2), 60.8 (CH_2), 55.3 (CH_3), 20.9 (CH_3), 20.8 (CH_3), 20.7 (CH_3), 20.7 (CH_3), 20.6 (CH_3), 20.6 (CH_3), 20.5 (CH_3) ppm.

IR (ATR): $\tilde{\nu}$ = 2957, 1770, 1712, 1617, 1563, 1312, 1198, 1134, 901, 862, 732 cm^{-1}.

MS (ESI-TOF): *m/z* (relative intensity) 775 (100) $[M+Na]^+$.

HRMS (ESI-TOF): *m/z* $[M+Na]^+$ Calcd. for $C_{35}H_{44}O_{18}Na^+$: 775.2420; Found: 775.2416.

(2*R*,3*S*,4*S*,5*R*,6*S*)-2-(acetoxymethyl)-6-(2*R*,3*R*,4*R*,5*S*,6*R*)-3,4,5-triacetoxy-6-(*E*)-4-methoxystyryl)tetrahydro-2*H*-pyran-2-yl)methoxy)tetrahydro-2*H*-pyran-3,4,5-triyl triacetate (3t)

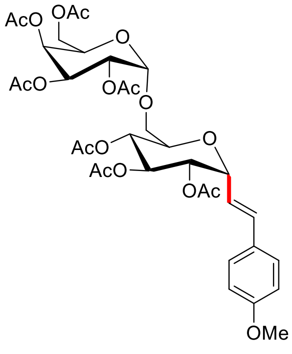

Product **3t** was obtained from melibiosyl bromide **1j** (139.9 mg, 0.20 mmol) and alkenyl bromide **2a** (63.9 mg, 0.30 mmol) according to *General Procedure 2*. Column chromatography (SiO_2, hexane/EtOAc 2:1) afforded **3t** (97.7 mg, 65%, $\alpha:\beta$ = 6:1) as a colorless syrup.

^{1}H-NMR (400 MHz, $CDCl_3$): δ 7.41 – 7.37 (m, 2H), 6.89 – 6.86 (m, 2H), 6.71 (dd, J = 16.0, 1.5 Hz, 1H), 6.21 (dd, J = 16.0, 6.3 Hz, 1H), 5.46 (dd, J = 3.4, 1.4 Hz, 1H), 5.41 – 5.35 (m, 2H), 5.18 (d, J = 3.7 Hz, 1H), 5.12 – 5.09 (m, 1H), 5.09 – 5.05 (m, 2H), 4.83 (td, J = 6.2, 1.6 Hz, 1H), 4.28 – 4.24 (m, 1H), 4.13 – 4.02 (m, 3H),

3.82 (s, 3H), 3.73 – 3.69 (m, 1H), 3.55 (dd, J = 11.4, 2.6 Hz, 1H), 2.13 (s, 3H), 2.06 (s, 3H), 2.04 (d, J = 1.2 Hz, 6H), 2.03 (s, 6H), 1.99 (s, 3H).

^{13}C{^{1}H}-NMR (101 MHz, $CDCl_3$): δ = 170.5 (C), 170.3 (C), 170.2 (C), 170.1 (C), 169.7 (C), 169.7 (C), 169.4 (C), 159.8 (C), 136.4 (CH), 128.6 (C), 128.0 (2x CH), 128.0 (CH), 118.1 (CH), 114.0 (2x CH), 96.1 (CH), 73.0 (CH), 70.8 (CH), 70.6 (CH), 70.0 (CH), 69.6 (CH), 68.0 (CH), 67.4 (CH), 66.6 (CH), 66.3 (CH), 61.6 (CH_2), 55.3 (CH_3), 20.7 (CH_3), 20.7 (CH_3), 20.7 (CH_3), 20.6 (CH_3), 20.6 (CH_3), 20.6 (CH_3), 20.6 (CH_3) ppm.

IR (ATR): $\tilde{\nu}$ = 2976, 1750, 1743, 1557, 1509, 1323, 1167, 1121, 860, 843, 767 cm^{-1}.

MS (ESI-TOF): *m/z* (relative intensity) 775 (100) [M+Na]$^+$.

HRMS (ESI-TOF): *m/z* [M+Na]$^+$ Calcd. for $C_{35}H_{44}O_{18}Na^+$: 775.2420; Found: 775.2418.

(2*R*,3*R*,4*S*,5*R*,6*S*)-2-(acetoxymethyl)-6-(2*R*,3*R*,4*R*,5*S*,6*R*)-3,4,5-triacetoxy-6-(*E*)-4-methoxystyryl)tetrahydro-2*H*-pyran-2-yl)methoxy)tetrahydro-2*H*-pyran-3,4,5-triyl triacetate (3u)

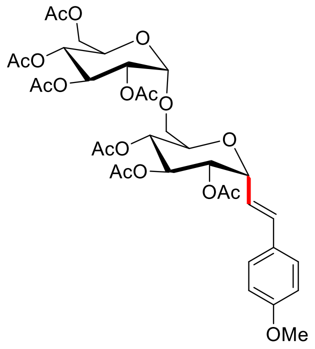

Product **3u** was obtained from isomaltosyl bromide **1l** (139.9 mg, 0.20 mmol) and alkenyl bromide **2a** (63.9 mg, 0.30 mmol) according to *General Procedure 2*. Column chromatography (SiO_2, hexane/EtOAc 2:1) afforded **3u** (106.4 mg, 71%, α:β = 6:1) as a colorless syrup.

^{1}H-NMR (400 MHz, $CDCl_3$): δ 7.39 – 7.34 (m, 2H), 6.91 – 6.87 (m, 2H), 6.68 (dd, J = 16.0, 1.5 Hz, 1H), 6.23 (dd, J = 16.0, 6.6 Hz, 1H), 5.39 (dd, J = 9.9, 8.5 Hz, 1H), 5.13 (d, J = 9.3 Hz, 1H), 5.08 – 5.03 (m, 2H), 4.95 – 4.91 (m, 1H), 4.79 (td, J = 6.4, 1.5 Hz, 1H), 4.53 (d, J = 7.9 Hz, 1H), 4.45 (dd, J = 11.9, 2.2 Hz, 1H), 4.38 (dd, J = 12.5, 4.4 Hz, 1H), 4.11 (dd, J = 11.9, 4.9 Hz, 1H), 4.05 (dd, J = 12.5, 2.3 Hz, 1H), 3.90 (ddd, J = 9.8, 4.9, 2.2 Hz, 1H), 3.83 (s, 3H), 3.75 – 3.70 (m, 1H), 3.69 – 3.65 (m, 1H), 2.13 (s, 3H), 2.09 (s, 3H), 2.05 (s, 3H), 2.02 (s, 3H), 2.01 (s, 6H), 1.98 (s, 3H).

^{13}C{^{1}H}-NMR (101 MHz, $CDCl_3$): δ = 170.6 (C), 170.2 (C), 170.2 (C), 169.9 (C), 169.7 (C), 169.6 (C), 169.5 (C), 159.8 (C), 136.3 (CH), 128.6 (C), 128.1 (2 x CH), 118.0 (CH), 114.0 (2 x CH), 95.7 (CH), 72.9 (CH), 70.8 (CH), 70.7 (CH), 70.7 (CH), 70.0 (CH), 70.0 (CH), 69.8 (CH), 68.4 (CH), 67.2 (CH), 67.1 (CH), 61.7 (CH_2), 55.3 (CH_3), 20.7 (CH_3), 20.7 (CH_3), 20.6 (2 x CH_3), 20.6 (CH_3), 20.5 (2 x CH_3) ppm.

IR (ATR): $\tilde{\nu}$ = 2969, 1726, 1720, 1643, 1563, 1321, 1187, 1143, 975, 836, 746 cm^{-1}.

MS (ESI-TOF): *m/z* (relative intensity) 775 (100) [M+Na]$^+$.

HRMS (ESI-TOF): *m/z* [M+Na]$^+$ Calcd. for $C_{35}H_{44}O_{18}Na^+$: 775.2420; Found: 775.2419.

(2*R*,3*R*,4*S*,5*R*,6*R*)-2-(acetoxymethyl)-6-(2*R*,3*R*,4*S*,5*R*,6*R*)-4,5-diacetoxy-2-(acetoxymethyl)-6-(2*R*,3*R*,4*S*,5*S*,6*R*)-4,5-diacetoxy-2-(acetoxymethyl)-6-(*E*)-4-methoxystyryl)tetrahydro-2*H*-pyran-3-yl)oxy)tetrahydro-2*H*-pyran-3-yl)oxy)tetrahydro-2*H*-pyran-3,4,5-triyl triacetate (3v)

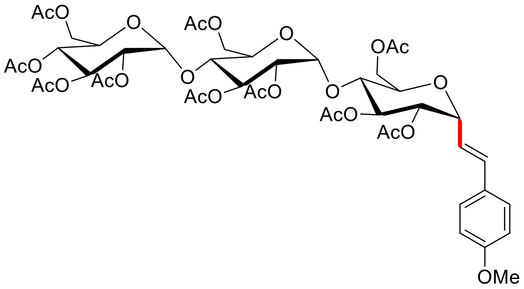

Product **3v** was obtained from maltotriosyl bromide **1m** (197.5 mg, 0.20 mmol) and alkenyl bromide **2a** (63.9 mg, 0.30 mmol) according to *General Procedure 2*. Column chromatography (SiO_2, hexane/EtOAc 1:1) afforded **3v** (115.0 mg, 55%, α:β > 20:1) as a colorless syrup.

^{1}H-NMR (400 MHz, $CDCl_3$): δ = 7.43 – 7.38 (m, 2H), 6.92 – 6.87 (m, 2H), 6.67 (dd, *J* = 16.0, 1.3 Hz, 1H), 6.21 (dd, *J* = 16.0, 6.2 Hz, 1H),, 5.41 – 5.37 (m, 2H), 5.35 – 5.30 (m, 2H), 5.26 (d, J = 4.0 Hz, 1H), 5.08 – 4.99 (m, 2H), 4.87 – 4.83 (m, 1H), 4.79 – 4.74 (m, 2H), 4.50 – 4.40 (m, 2H), 4.31 – 4.22 (m, 2H), 4.20 – 4.15 (m, 1H), 4.05 (ddd, J = 15.7, 5.5, 3.1 Hz, 3H), 3.96 – 3.91 (m, 2H), 3.86 (dd, J = 9.0, 7.2 Hz, 1H), 3.83 (s, 3H), 2.17 (s, 3H), 2.14 (s, 3H), 2.09 (s, 3H), 2.04 (s, 3H), 2.04 – 2.02 (m, 9H), 2.02 (s, 3H), 1.99 (s, 3H), 1.98 (s, 3H).

^{13}C{^{1}H}-NMR (101 MHz, $CDCl_3$): δ = 170.5 (2 x C), 170.5 (C), 170.5 (C), 170.3 (C), 170.0 (C), 169.8 (C), 169.7 (C), 169.6 (C), 169.4 (C), 159.8 (C), 135.6 (CH), 128.7 (C), 128.1 (2 x CH), 118.8 (CH), 114.0 (2 x CH), 96.1 (CH), 95.6 (CH), 74.7 (CH), 72.8 (CH), 72.5 (CH), 72.3 (CH), 71.8 (CH), 70.7 (CH), 70.4 (CH), 70.0 (CH), 70.0 (CH), 69.3 (CH), 68.8 (CH), 68.4 (CH), 67.8 (CH), 63.3 (CH_2), 62.3 (CH_2), 61.3 (CH_2), 55.3 (CH_3), 20.9 (CH_3), 20.9 (CH_3), 20.8 (CH_3), 20.7 (CH_3), 20.7 (CH_3), 20.6 (CH_3), 20.5 (CH_3), 20.5 (3 x CH_3) ppm.

IR (ATR): $\tilde{\nu}$ = 2965, 1796, 1743, 1721, 1623, 1546, 1213, 1143, 996, 823 721 cm^{-1}.

MS (ESI-TOF): *m/z* (relative intensity) 1063 (100) [M+Na]$^+$.

HRMS (ESI-TOF): *m/z* [M+Na]$^+$ Calcd. for $C_{47}H_{60}O_{26}Na^+$: 1063.3265; Found: 1063.3265.

(2*R*,3*S*,4*R*,5*S*,6*R*)-2-(((4-(*N*,*N*-dipropylsulfamoyl)benzoyl)oxy)methyl)-6-((*E*)-4-methoxystyryl)tetrahydro-2*H*-pyran-3,4,5-triyl triacetate (3w)

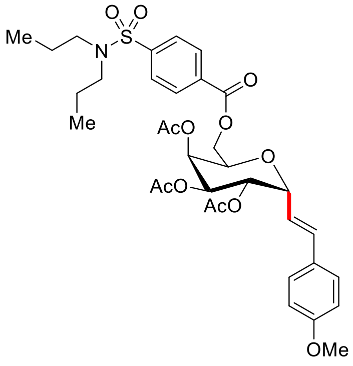

Product **3w** was obtained from **1n** (127 mg, 0.20 mmol) and alkenyl bromide **2a** (63.9 mg, 0.30 mmol) according to *General Procedure 2*. Column chromatography (SiO_2, hexane/EtOAc 4:1) afforded **3w** (86.9 mg, 63%, α:β > 20:1) as a colorless syrup.

^{1}H-NMR (400 MHz, $CDCl_3$): δ = 8.12 – 8.09 (m, 2H), 7.87 – 7.84 (m, 2H), 7.31 – 7.28 (m, 2H), 6.86 (d, J = 8.7 Hz, 2H), 6.71 (dd, J = 16.0, 1.7 Hz, 1H), 6.19 (dd, J = 16.1, 5.7 Hz, 1H), 5.53 (dd, J = 3.4, 1.6 Hz, 1H), 5.42 (dd, J = 10.5, 6.0 Hz, 1H), 5.26 (dd, J = 10.6, 3.3 Hz, 1H), 4.98 (td, J = 5.8, 1.8 Hz, 1H), 4.52 (dd, J = 11.0, 6.7 Hz, 1H), 4.38 (d, J = 1.7 Hz, 1H), 4.30 (dd, J = 10.9, 5.9 Hz, 1H), 3.82 (s, 3H), 3.10 – 3.06 (m, 4H), 2.19 (s, 3H), 2.06 (s, 3H), 2.03 (s, 3H), 1.57 – 1.51 (m, 4H), 0.86 (t, J = 7.4 Hz, 6H) ppm.

^{13}C{^{1}H}-NMR (101 MHz, $CDCl_3$): δ = 170.2 (C), 170.1 (C), 169.9 (C), 164.6 (C), 159.8 (C), 144.5 (C), 135.7 (CH), 132.7 (C), 130.3 (2 x CH), 128.5 (C), 127.8 (2 x CH), 127.0 (2 x CH), 118.0 (CH), 114.1 (2 x CH), 73.2 (CH), 68.4 (CH), 68.1 (CH), 68.1 (CH), 68.0 (CH), 62.8 (CH_2), 55.3 (CH_3), 49.9 (2 x CH_2), 21.9 (2 x CH_2), 20.8 (CH_3), 20.7 (2 x CH_3), 11.1 (2 x CH_3) ppm.

IR (ATR): $\tilde{\nu}$ = 2967, 1746, 1720, 1606, 1576, 1511, 1369, 1104, 910, 727 cm^{-1}.

MS (ESI-TOF): *m/z* (relative intensity) 712 (100) $[M+Na]^+$.

HRMS (ESI-TOF): *m/z* $[M+Na]^+$ Calcd. for $C_{34}H_{43}NO_{12}SNa^+$: 712.2398; Found: 712.2395.

(2*R*,3*S*,4*R*,5*S*,6*R*)-2-((2-(2-((2,6-dichlorophenyl)amino)phenyl)acetoxy)methyl)-6-((*E*)-4-methoxystyryl)tetrahydro-2*H*-pyran-3,4,5-triyl triacetate (3x)

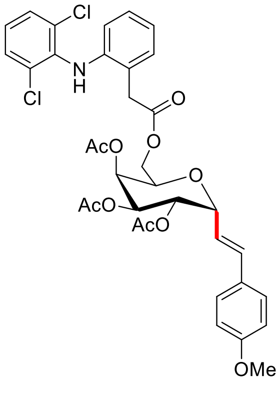

Product **3x** was obtained from **1o** (129 mg, 0.20 mmol) and alkenyl bromide **2a** (63.9 mg, 0.30 mmol) according to *General Procedure 2*. Column chromatography (SiO_2, hexane/EtOAc 4:1) afforded **3x** (77.1 mg, 55%, α:β > 20:1) as a colorless syrup.

^{1}H-NMR (400 MHz, $CDCl_3$): δ = 7.35 – 7.29 (m, 4H), 7.19 (dd, J = 7.6, 1.6 Hz, 1H), 7.10 (td, J = 7.7, 1.6 Hz, 1H), 7.00 – 6.94 (m, 1H), 6.91 (td, J = 7.5, 1.2 Hz, 1H), 6.88 – 6.83 (m, 2H), 6.78 (s, 1H), 6.69 (dd, J = 16.0, 1.6 Hz, 1H), 6.52 (d, J = 8.1 Hz, 1H), 6.15 (dd, J = 16.0, 5.9 Hz, 1H), 5.47 (dd, J = 3.2, 1.3 Hz, 1H), 5.37 (dd, J = 10.4, 5.8 Hz, 1H), 5.24 (dd, J = 10.4, 3.3 Hz, 1H), 4.94 (td, J = 5.8, 1.7 Hz, 1H), 4.32 – 4.24 (m, 2H), 4.21 – 4.15 (m, 1H), 3.82 (s, 5H), 2.15 (s, 3H), 2.05 (s, 3H), 2.03 (s, 3H) ppm.

$^{13}C\{^1H\}$-NMR (101 MHz, $CDCl_3$): δ = 172.0 (C), 170.3 (C), 170.2 (C), 170.0 (C), 159.9 (C), 142.83 (C), 137.93 (C), 135.8 (CH), 131.0 (CH), 129.6 (C), 128.9 (2 x CH), 128.8 (C), 128.2 (CH), 128.0 (2 x CH), 124.1 (CH), 124.1 (C), 122.2 (CH), 118.4 (CH), 118.2 (CH), 114.1 (2 x CH), 73.3 (CH), 68.5 (CH), 68.4 (CH), 68.3 (CH), 68.2 (CH), 62.8 (CH_2), 55.4 (CH), 38.3 (CH_2), 20.9 (CH_3), 20.8 (CH_3), 20.8 (CH_3) ppm.

IR (ATR): $\tilde{\nu}$ = 3341, 3011, 2957, 2930, 2837, 1742, 1606, 1510, 1452, 1369, 1215, 1052, 907, 727, 648 cm^{-1}.

MS (ESI-TOF): *m/z* (relative intensity) 722 (100) $[M+Na]^+$.

HRMS (ESI-TOF): *m/z* $[M+Na]^+$ Calcd. for $C_{35}H_{35}Cl_2NO_{10}Na^+$: 722.1530; Found: 722.1527.

Divided-cell electrolysis experiment

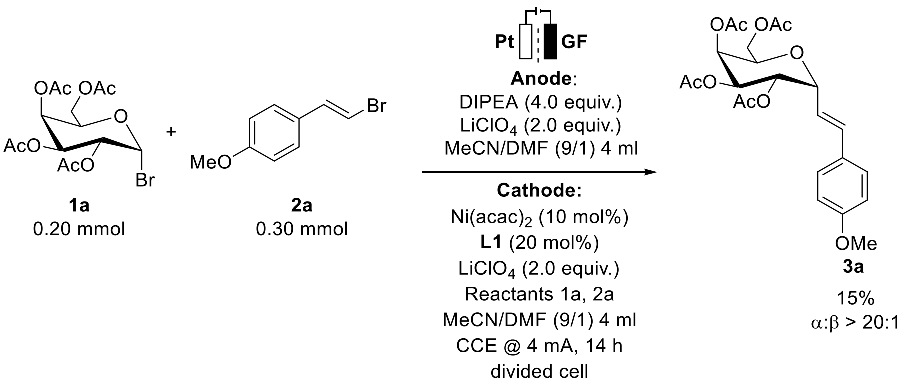

Under the divided-cell electrolysis conditions described above, the reaction afforded alkenyl glycoside **3a** in 15% isolated yield with preserved stereoselectivity. Additionally, 37% of glycal byproducts were detected. These results suggest that low-valent nickel species can reduce glycosyl bromide to generate glycosyl radicals, although this appears to be a minor pathway. The data underscore the importance of DIPEA in facilitating radical generation via the α-amino radical pathway through the electrochemical XAT (e-XAT) process. The enhanced reactivity observed in the presence of Hünig's base highlights its role as an effective XAT mediator in promoting the transformation with the proposed mechanism.

Cyclic voltammetry studies

CV measurements were conducted with a Metrohm Autolab PGSTAT204 potentiostat and Nova 2.1 software. A glassy carbon working electrode (disk, diameter: 3mm), a coiled platinum wire counter electrode, and a standard calomel electrode were employed. The voltammograms were recorded at room temperature in CH_3CN/DMF (9/1), at a substrate concentration of 25 mM and 0.1 mM $TBAPF_6$ as

supporting electrolytes. All solutions were degassed with N_2 before the measurement. The scan rate is 100 mV/s. The plotting is according to the IUPAC convention. The scan was initiated at -1.8V and swept in positive direction to +1.8V.

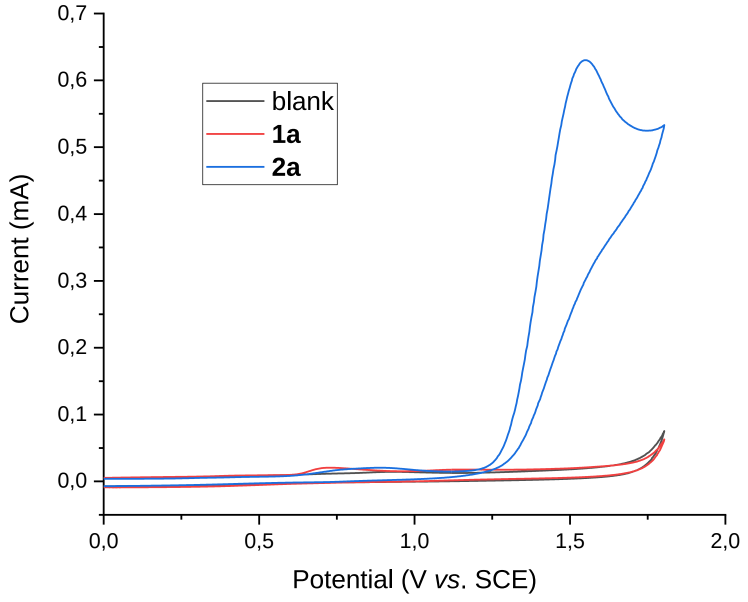

Figure S1. Cyclic voltammograms of glycosyl donor and alkenyl bromide.

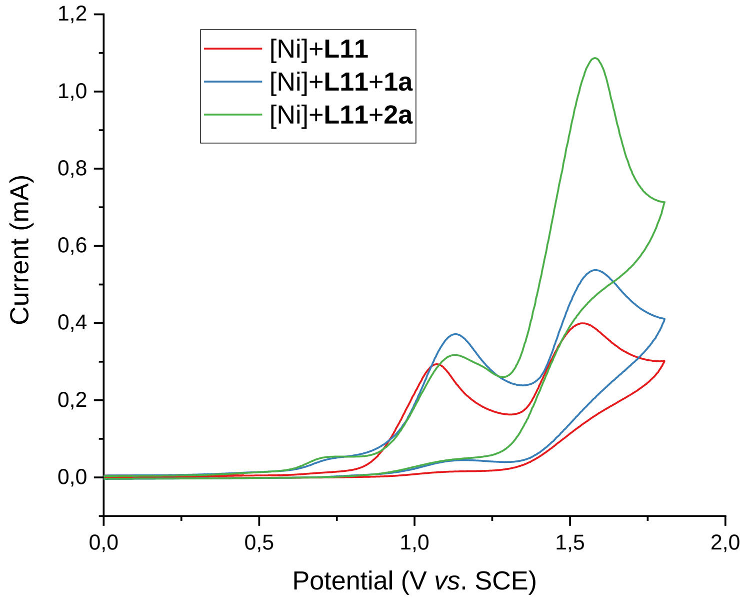

Figure S2. Cyclic voltammograms of Ni-catalyst with reactants.[Ni] = Ni(acac)$_2$

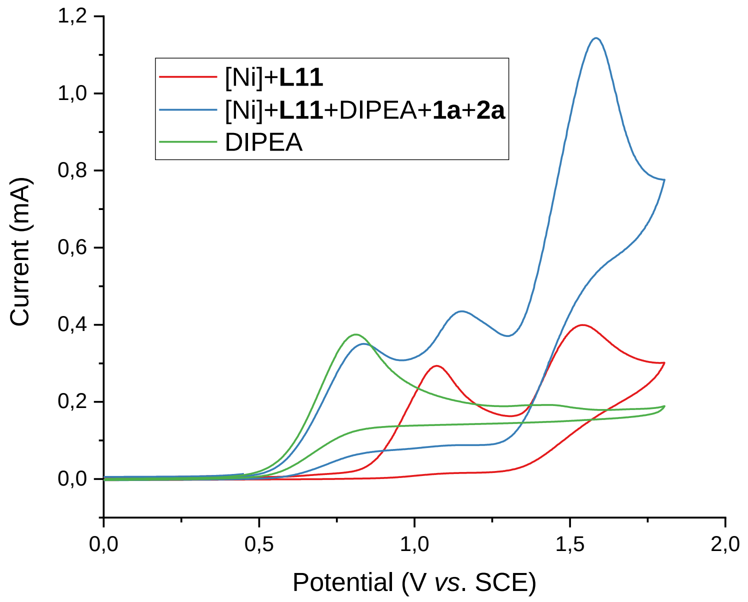

Figure S3. Cyclic voltammograms of reactants. [Ni] = Ni(acac)$_2$

II. Spectral Data for Compounds

Compound **3a** ^{1}H-NMR (400 MHz, $CDCl_3$)

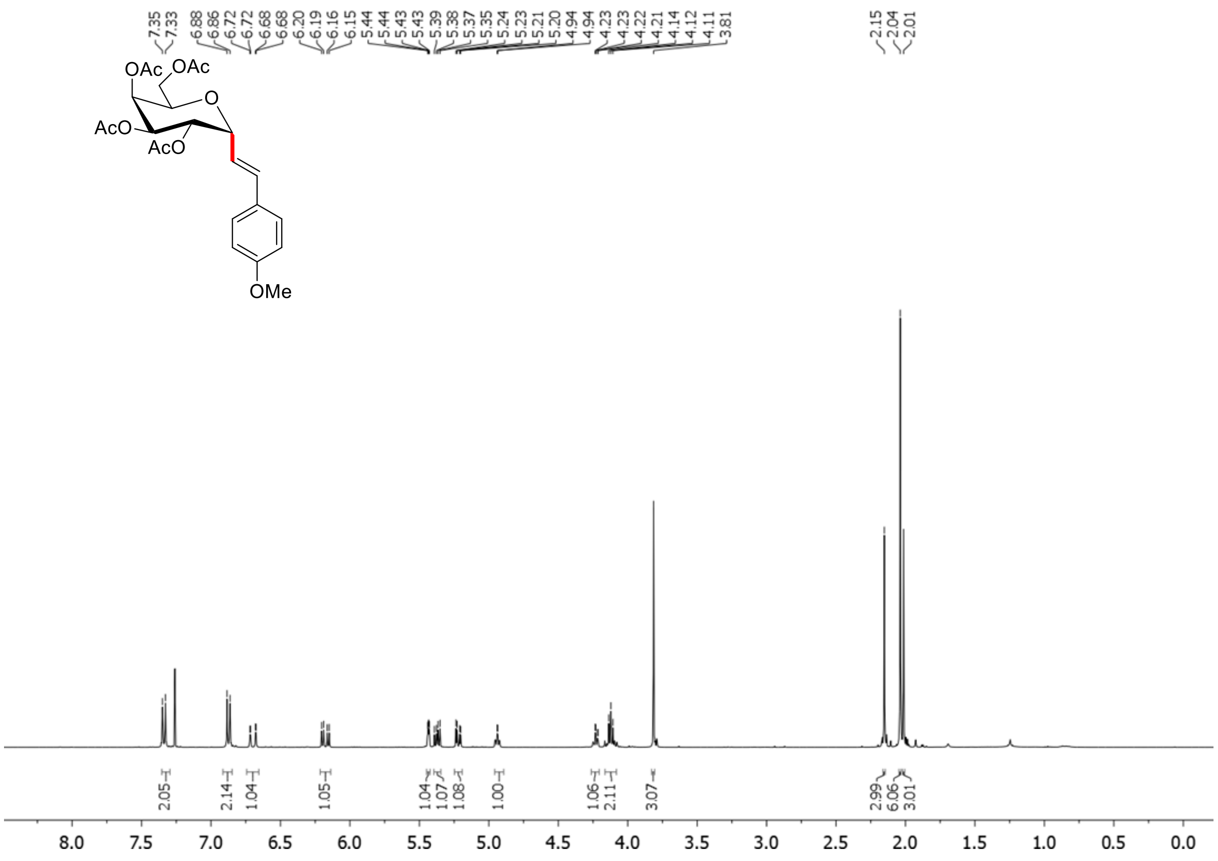

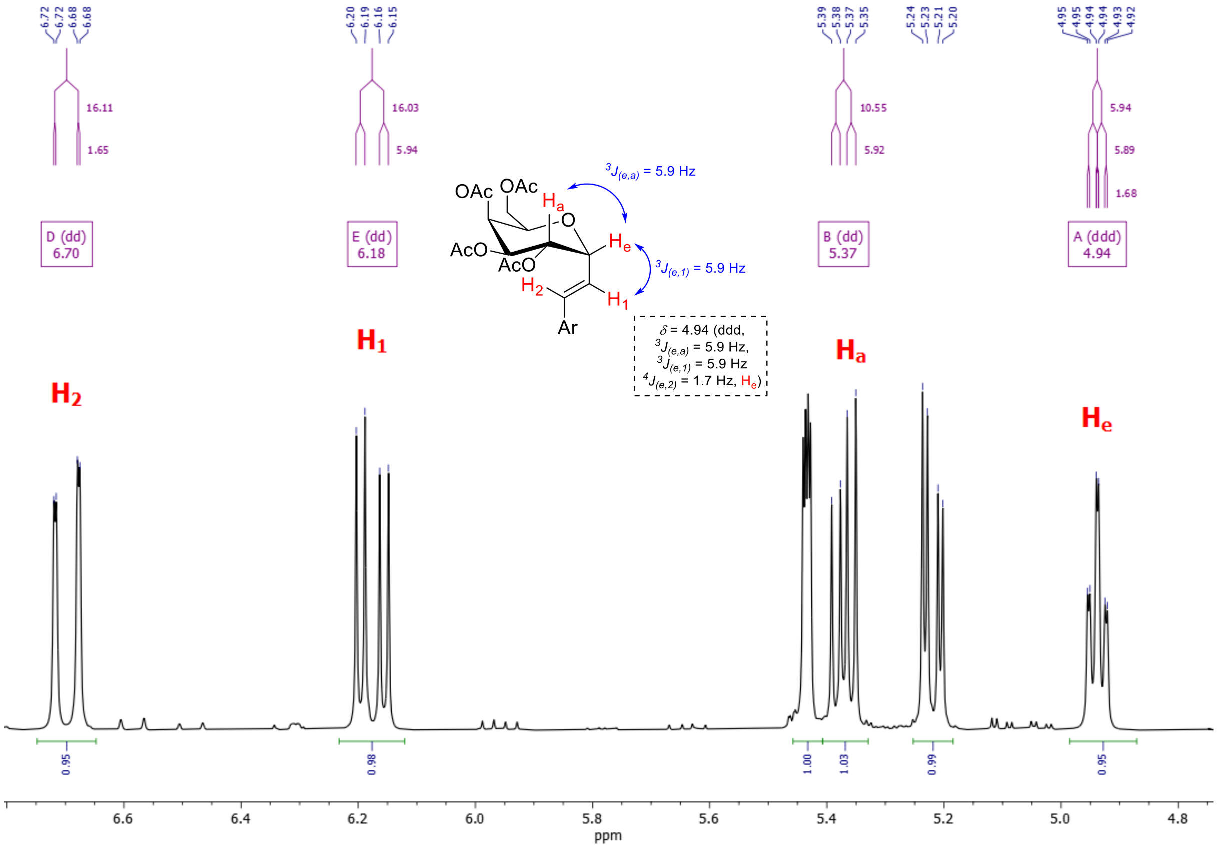

Compound **3a** $^{13}C\{^{1}H\}$-NMR (101 MHz, $CDCl_3$)

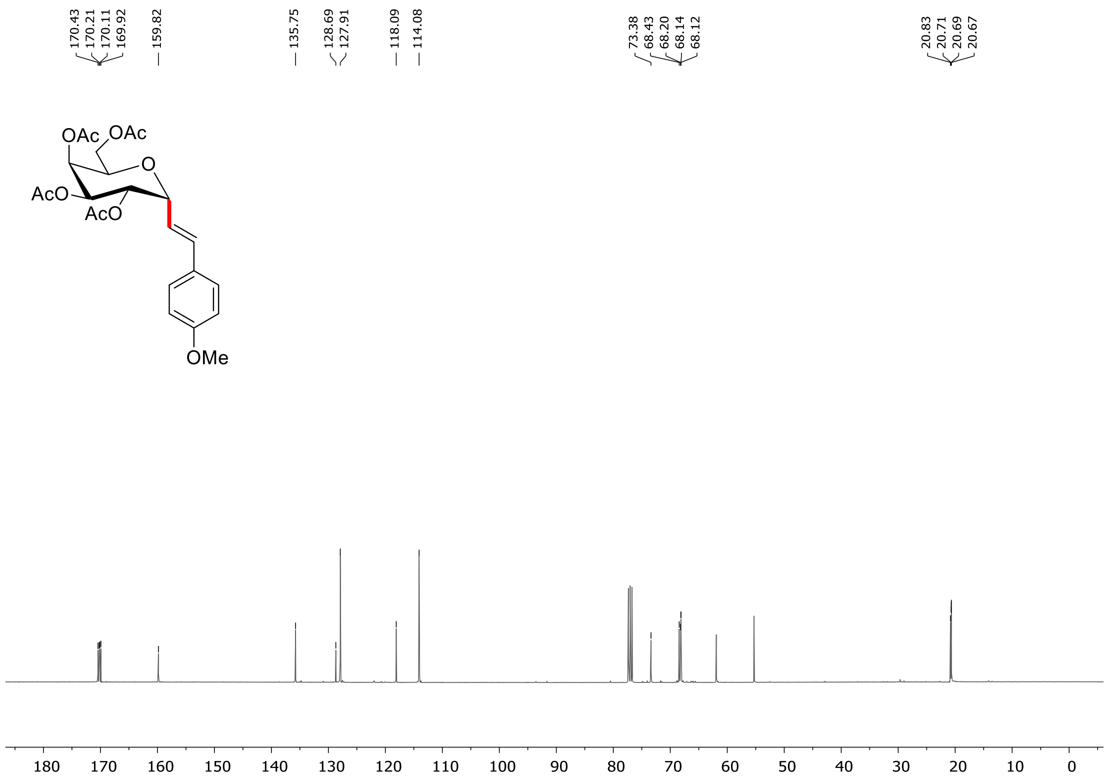

Compound **3a** ^{1}H-^{1}H-Cosy ($CDCl_3$)

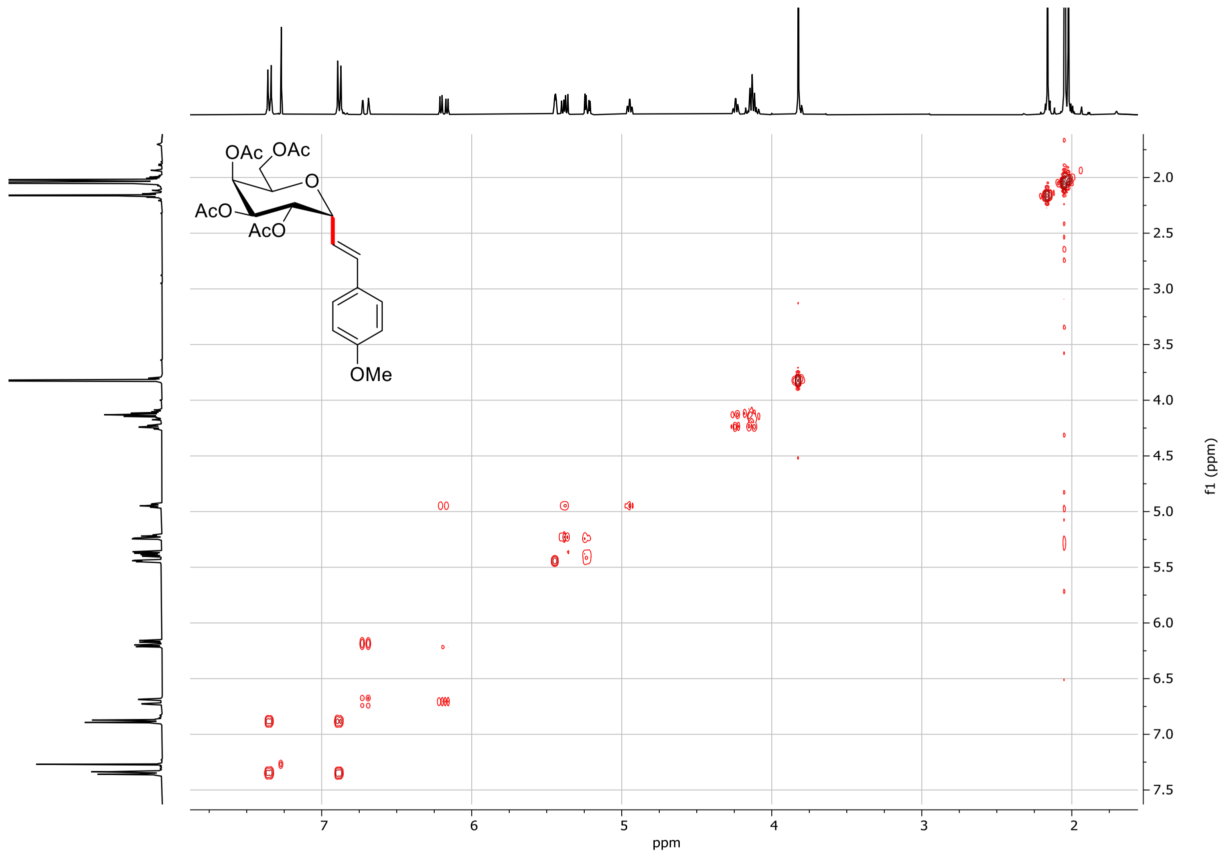

Compound **3a** HSQC ($CDCl_3$)

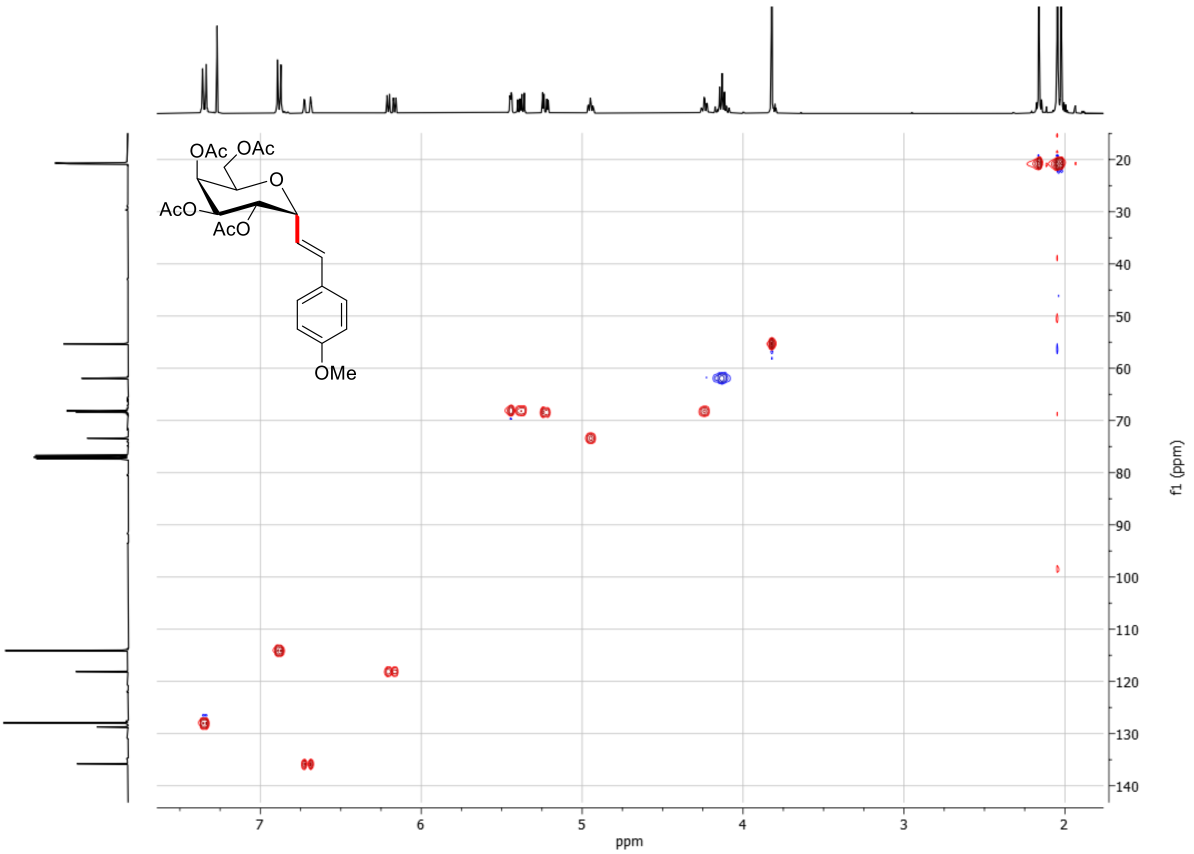

Compound **3a** HMBC ($CDCl_3$)

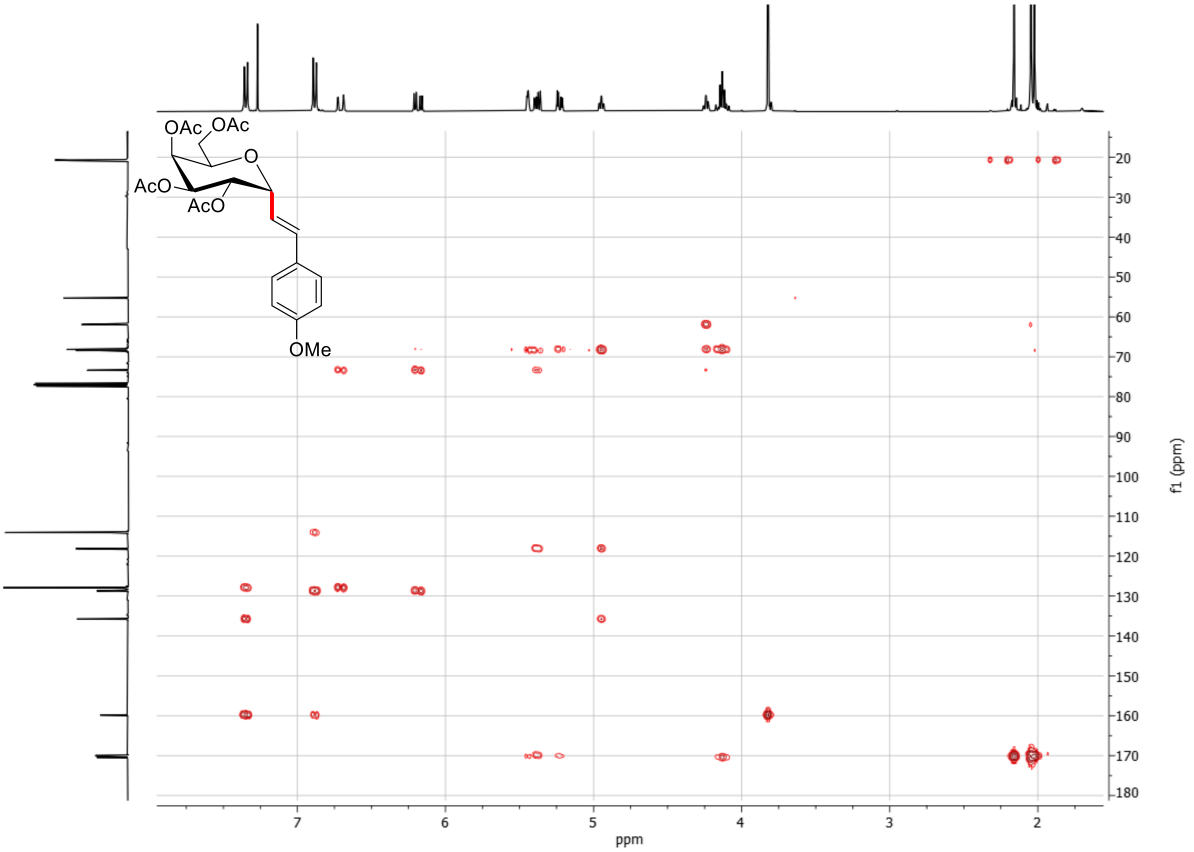

Compound **3a** HMBC (600 MHz, $CDCl_3$)

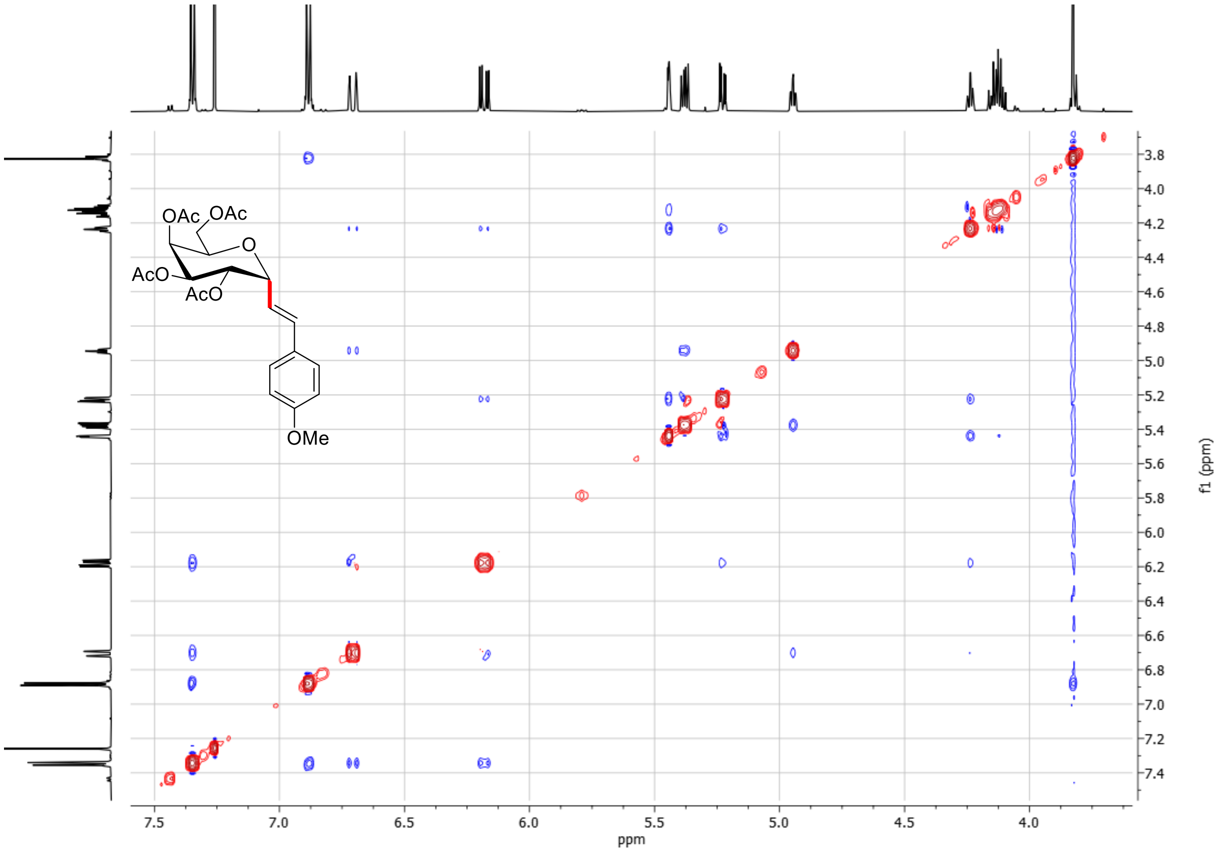

Compound **3b** ^{1}H-NMR (400 MHz, $CDCl_3$)

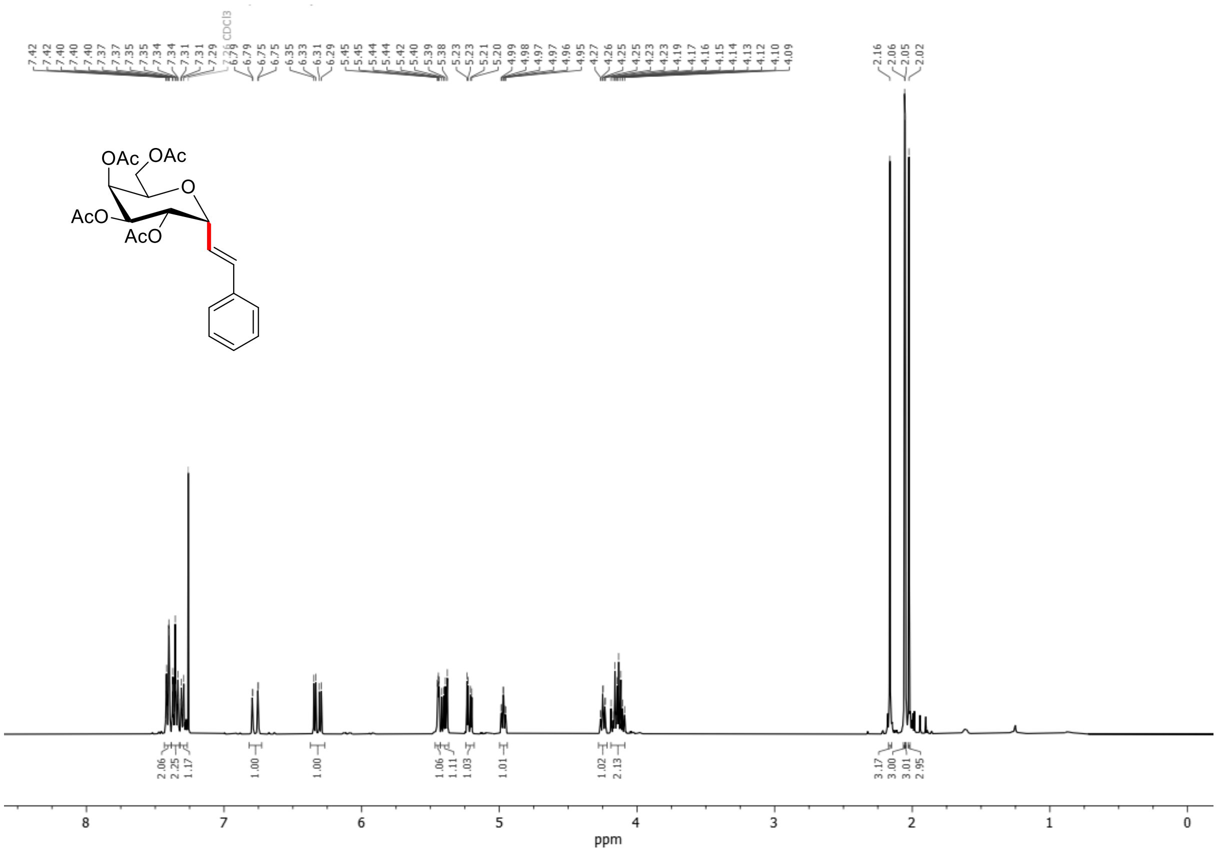

Compound **3b** $^{13}C\{^{1}H\}$-NMR (101 MHz, $CDCl_3$)

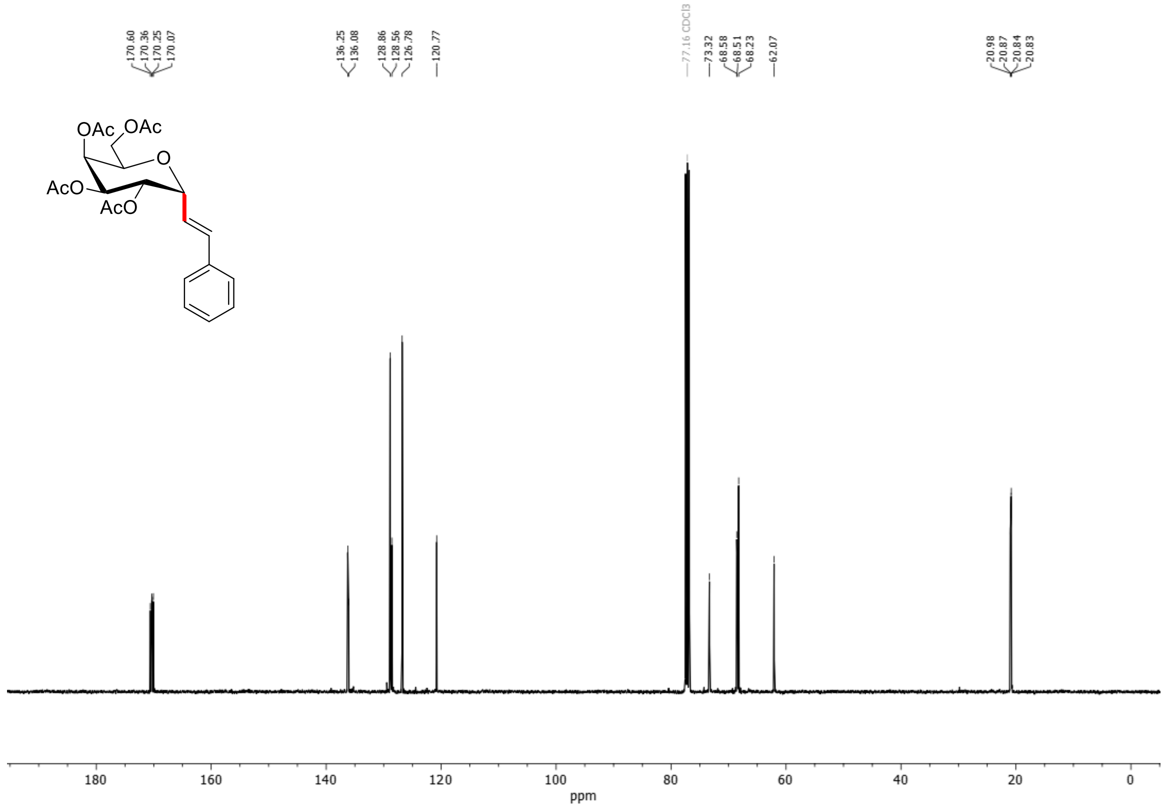

Compound **3c** ^{1}H-NMR (400 MHz, $CDCl_3$)

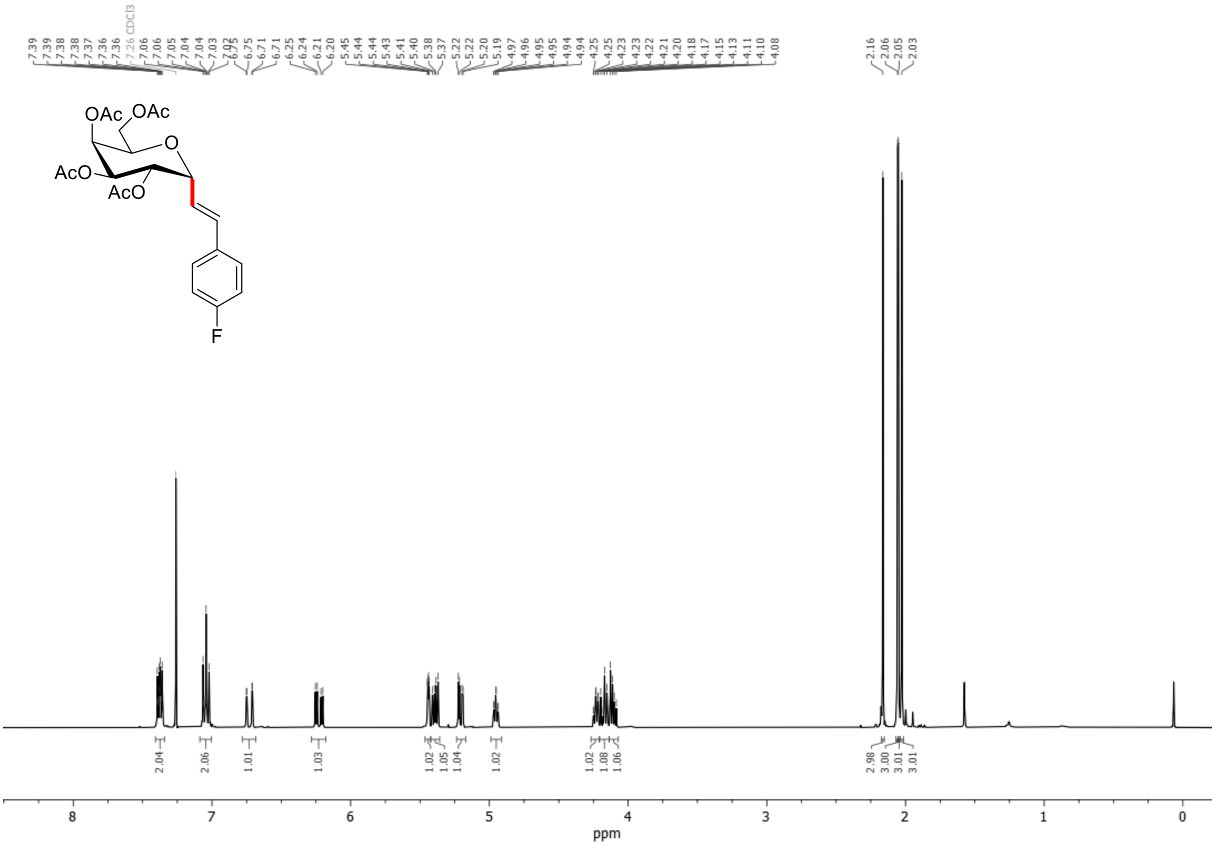

Compound **3c** $^{13}C\{^{1}H\}$-NMR (101 MHz, $CDCl_3$)

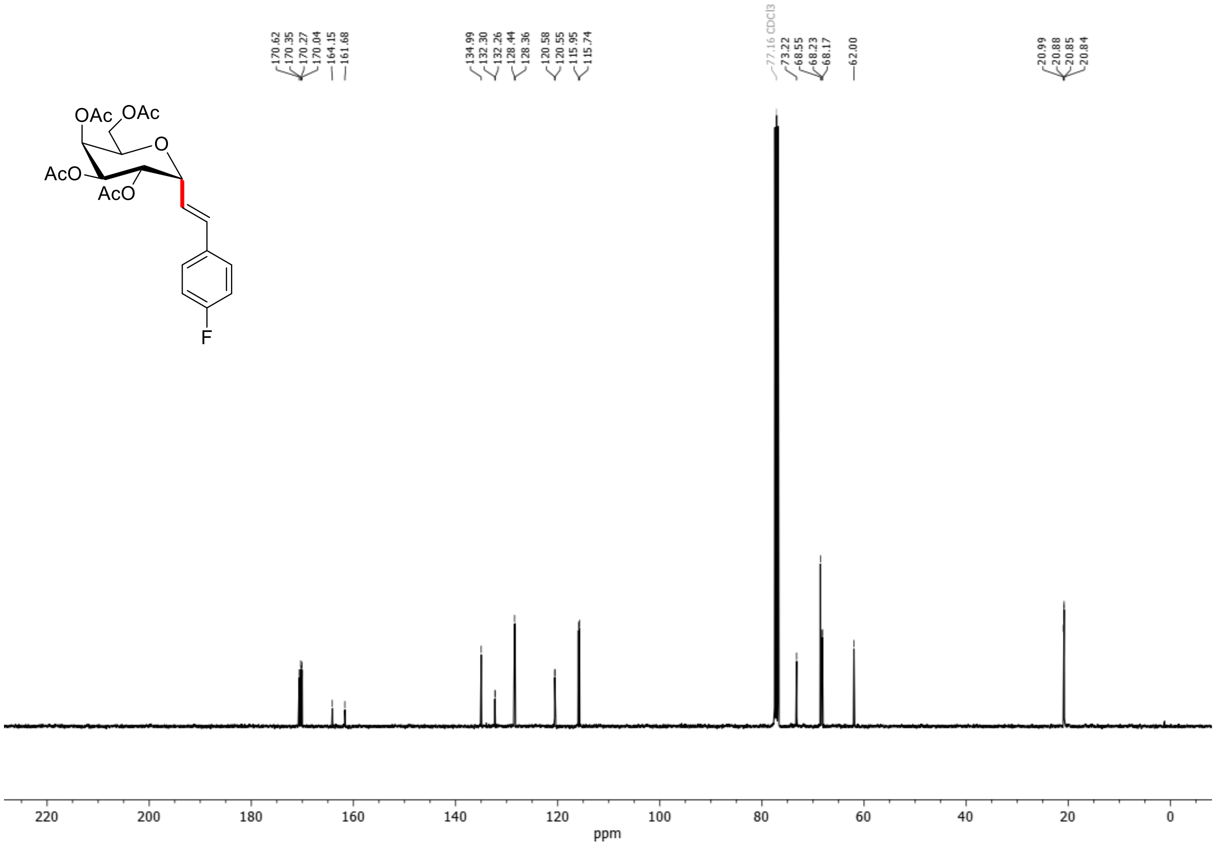

Compound **3c** ^{19}F-NMR (377 MHz, $CDCl_3$)

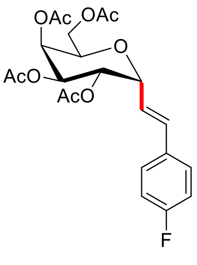

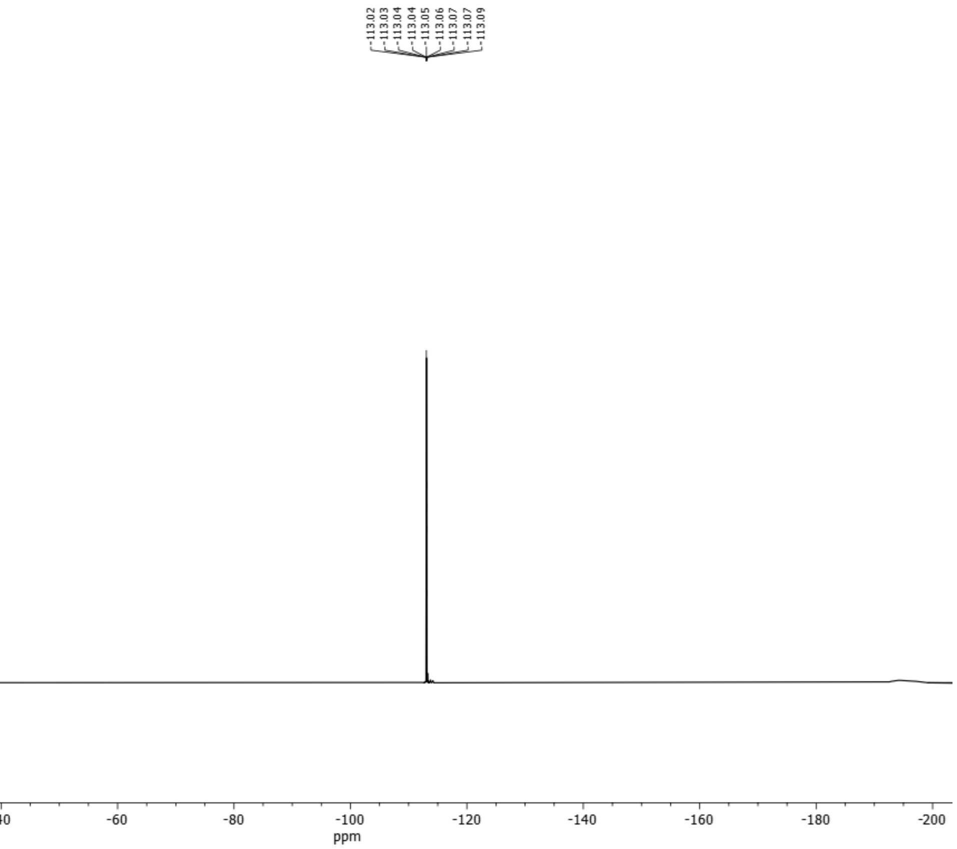

Compound **3d** ^{1}H-NMR (400 MHz, $CDCl_3$)

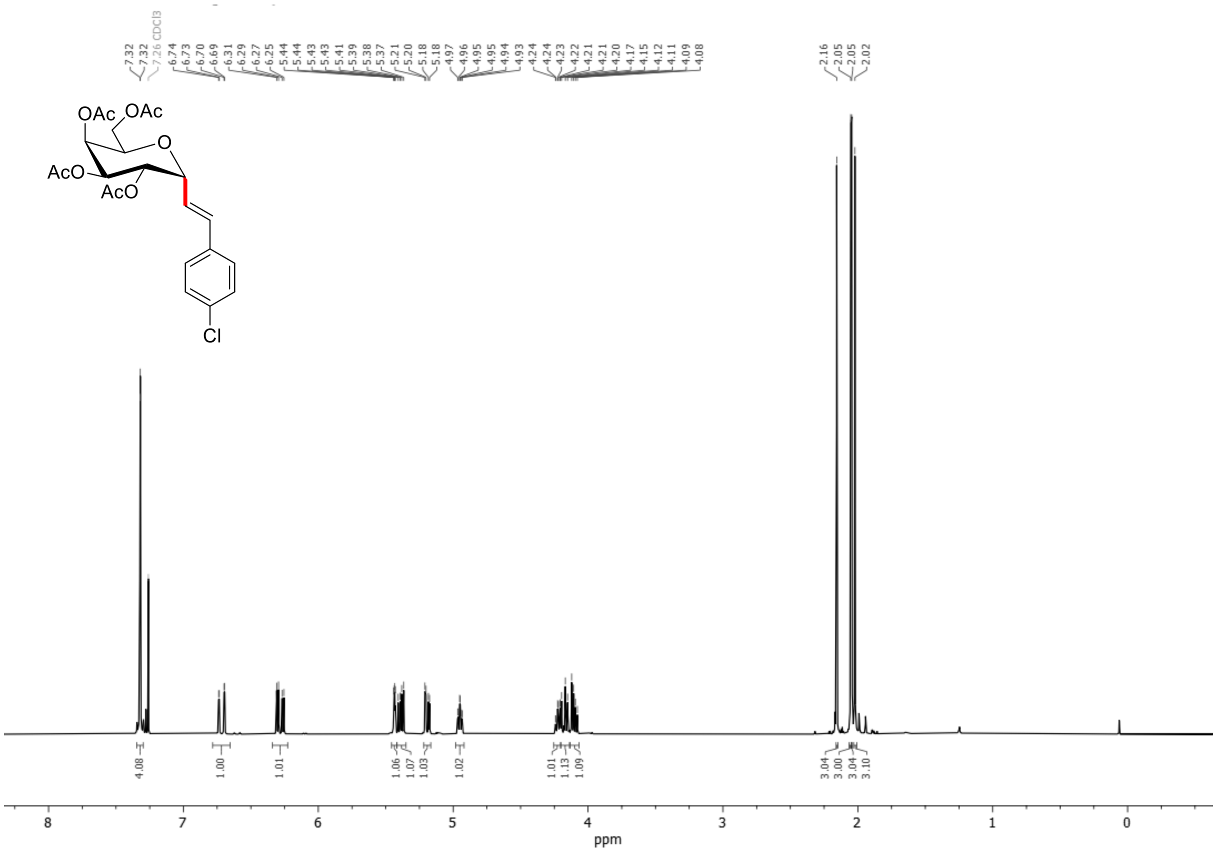

Compound **3d** $^{13}C\{^{1}H\}$-NMR (101 MHz, $CDCl_3$)

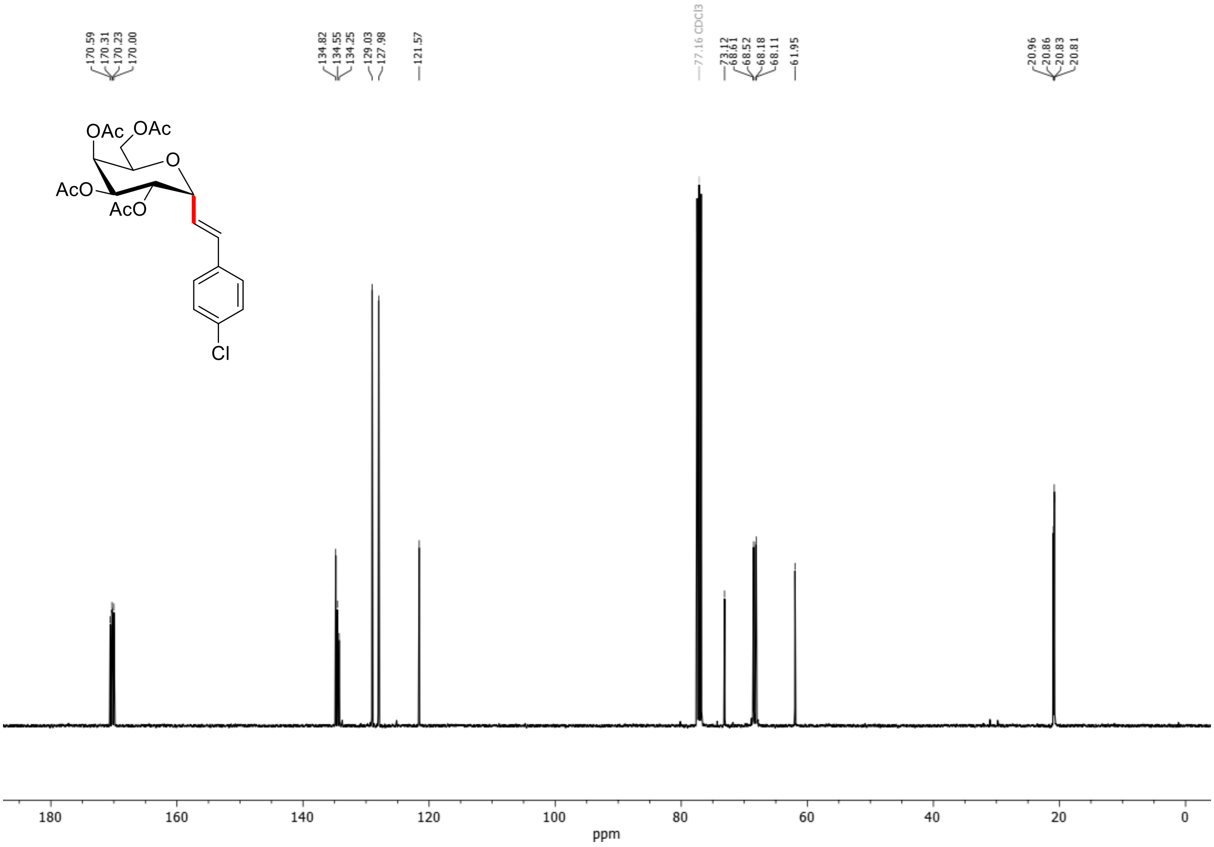

Compound **3e** ^{1}H-NMR (400 MHz, $CDCl_3$)

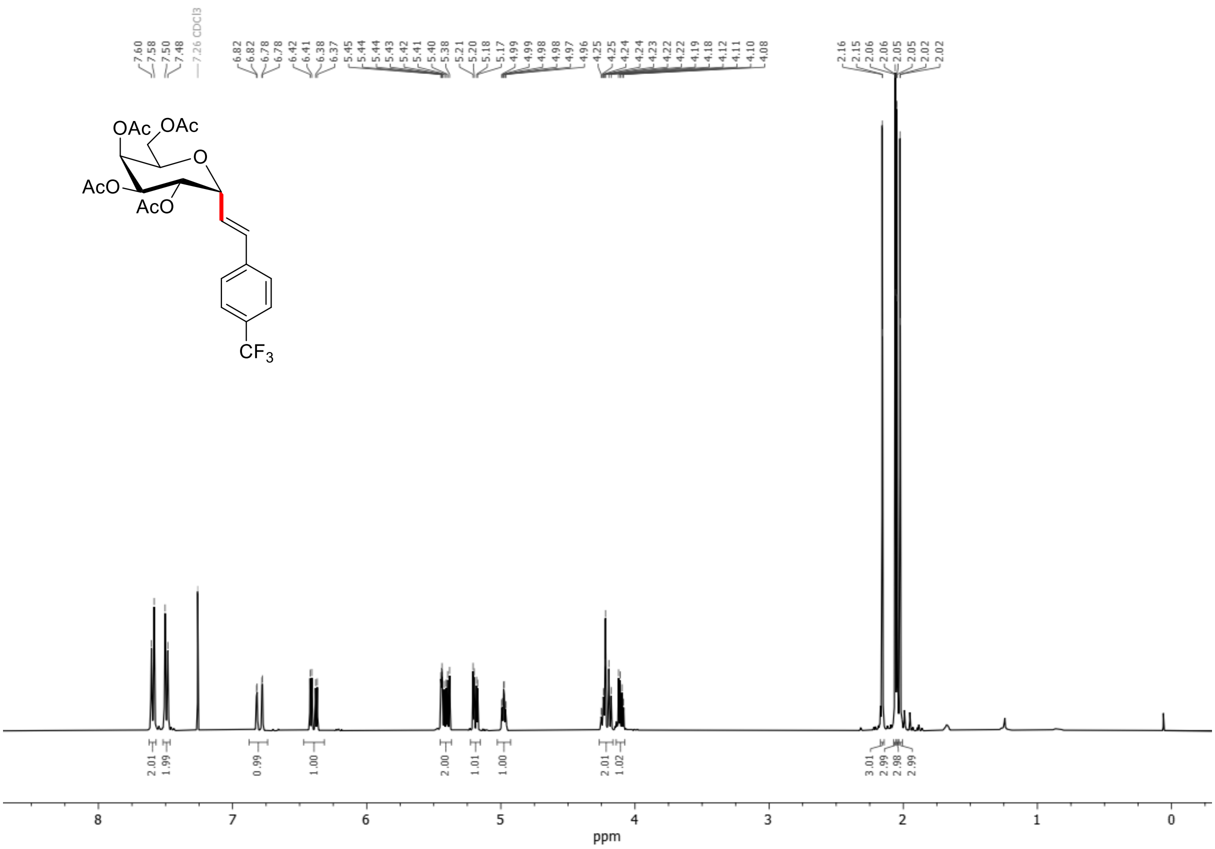

Compound **3e** ^{13}C{^{1}H}-NMR (101 MHz, $CDCl_3$)

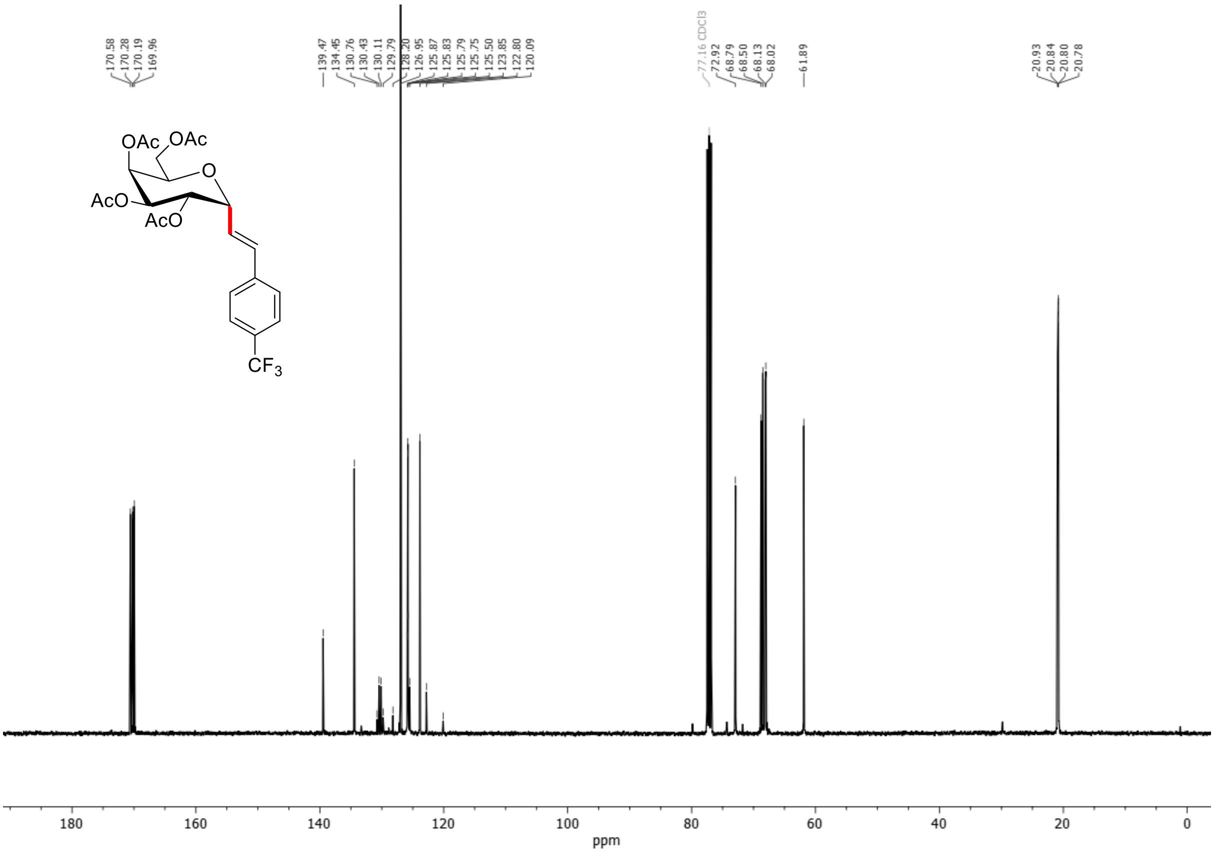

Compound **3e** ^{19}F-NMR (377 MHz, $CDCl_3$)

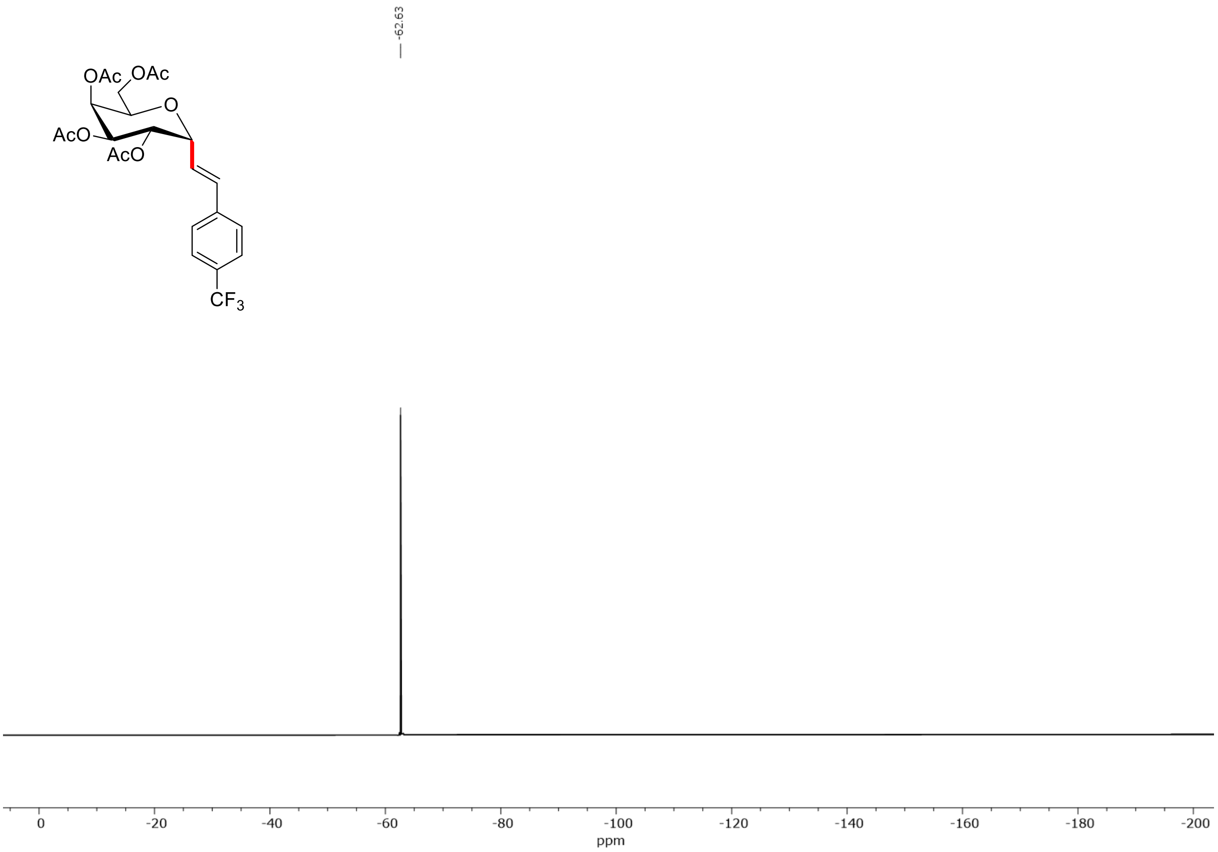

Compound **3f** ^{1}H-NMR (400 MHz, $CDCl_3$)

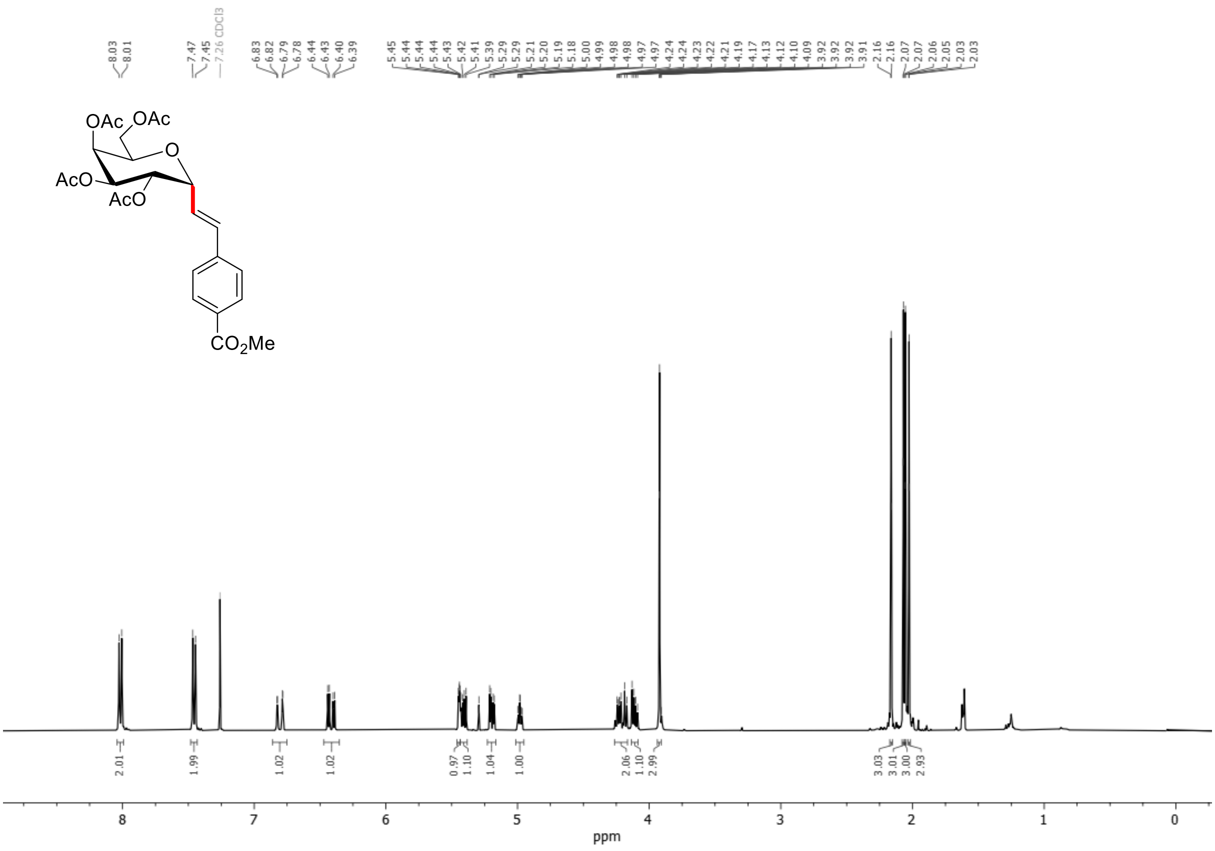

Compound **3f** $^{13}C\{^{1}H\}$-NMR (101 MHz, $CDCl_3$)

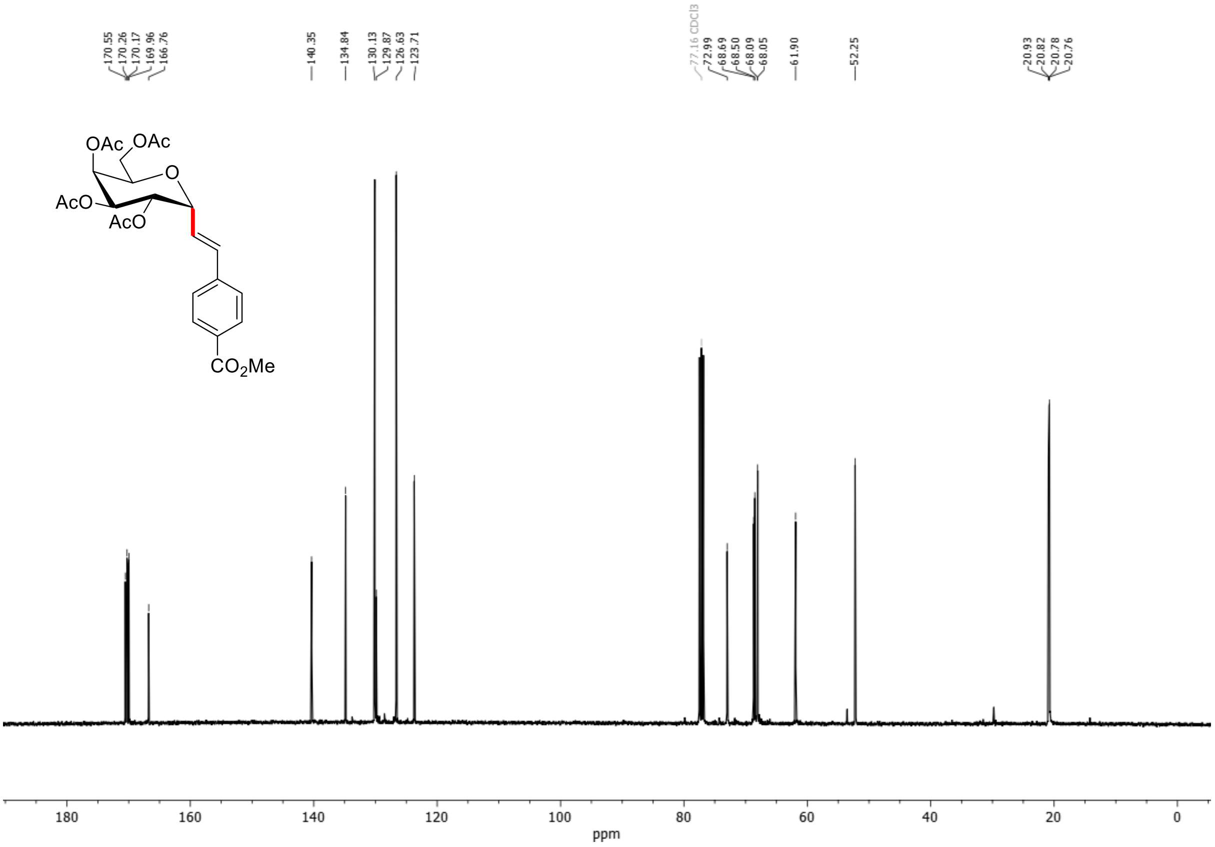

Compound **3g** ^{1}H-NMR (400 MHz, $CDCl_3$)

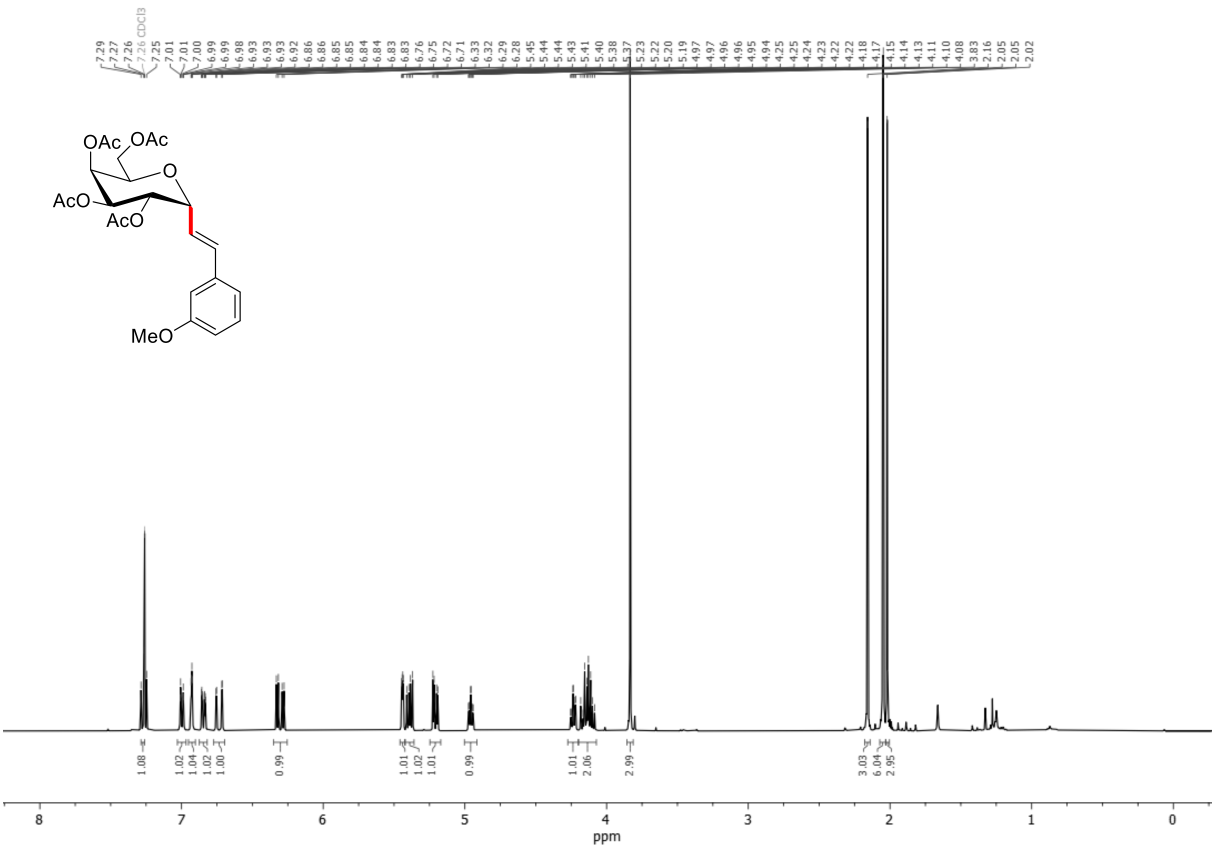

Compound **3g** ^{13}C{^{1}H}-NMR (101 MHz, $CDCl_3$)

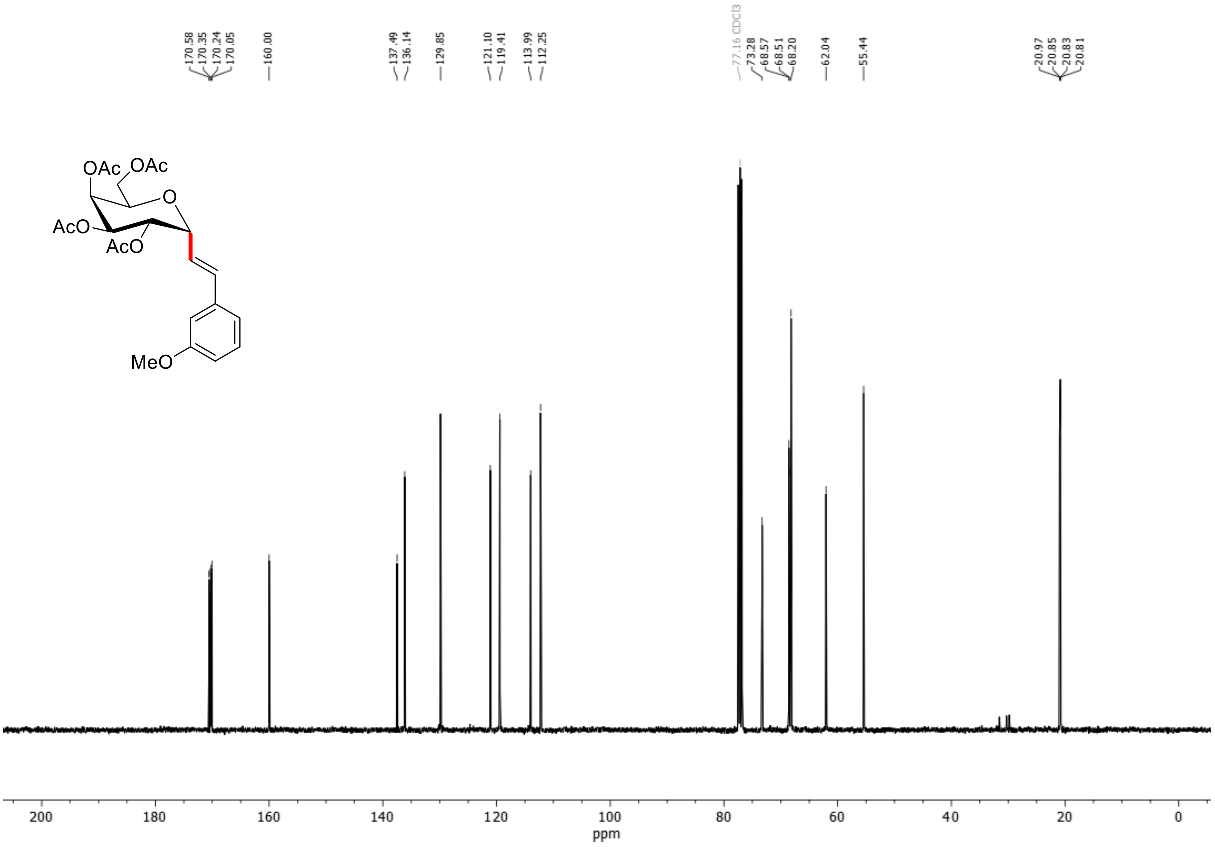

Compound **3h** ^{1}H-NMR (400 MHz, $CDCl_3$)

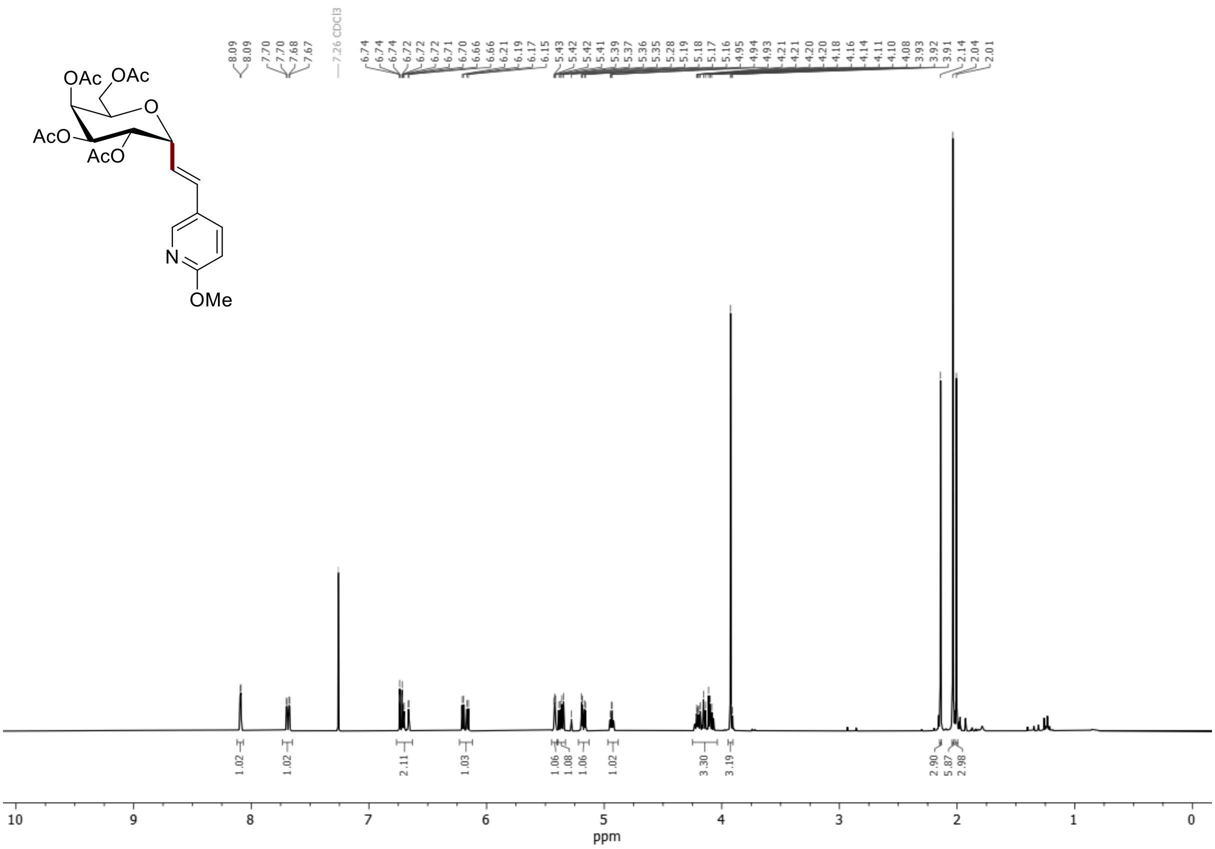

Compound **3h** ^{13}C{^{1}H}-NMR (101 MHz, $CDCl_3$)

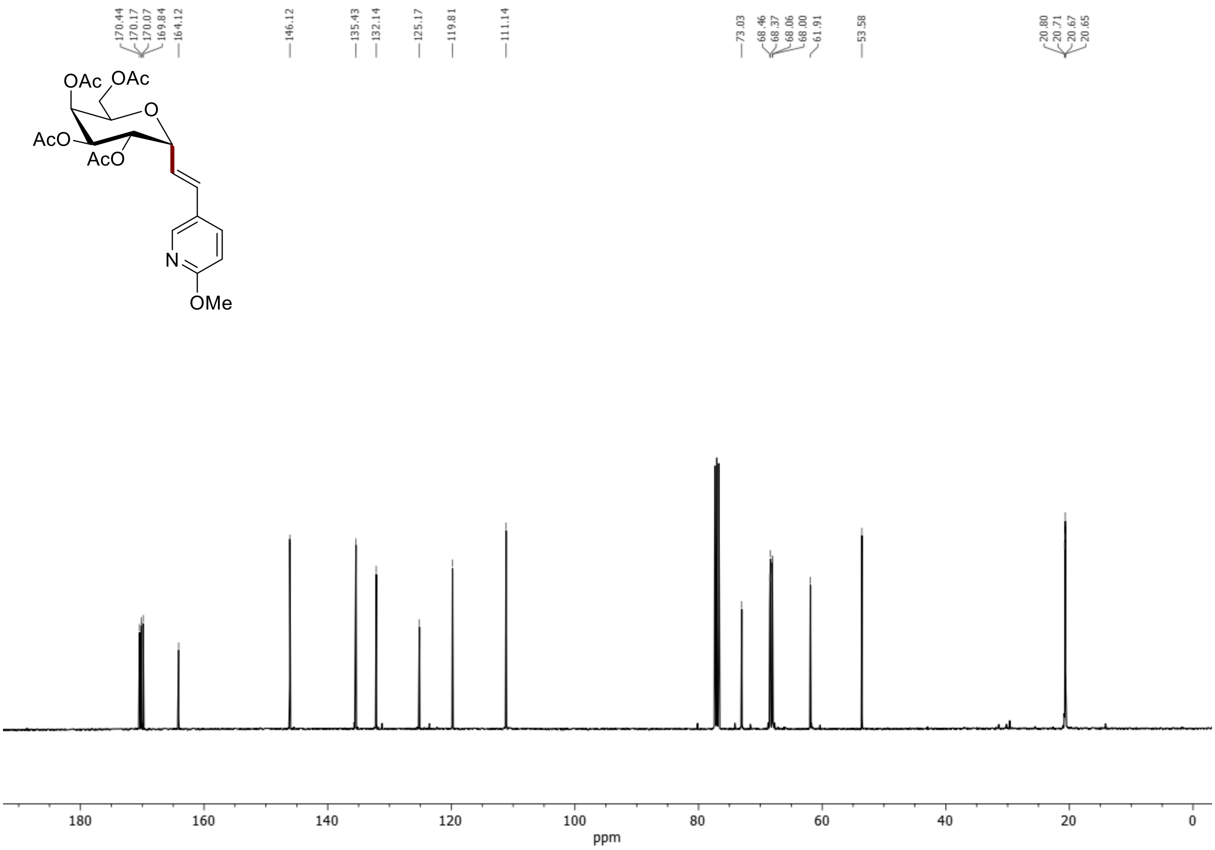

Compound **3i** ^{1}H-NMR (300 MHz, $CDCl_3$)

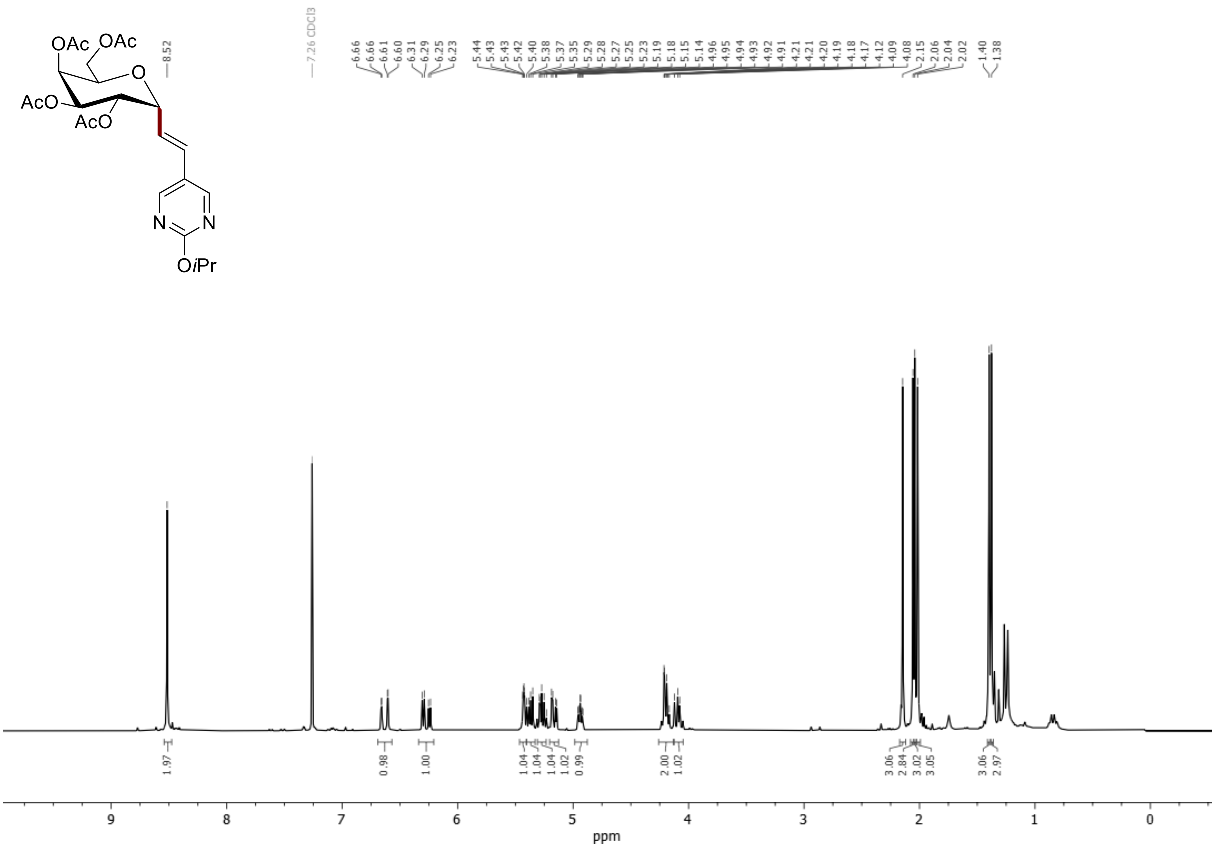

Compound **3i** $^{13}C\{^{1}H\}$-NMR (75 MHz, $CDCl_3$)

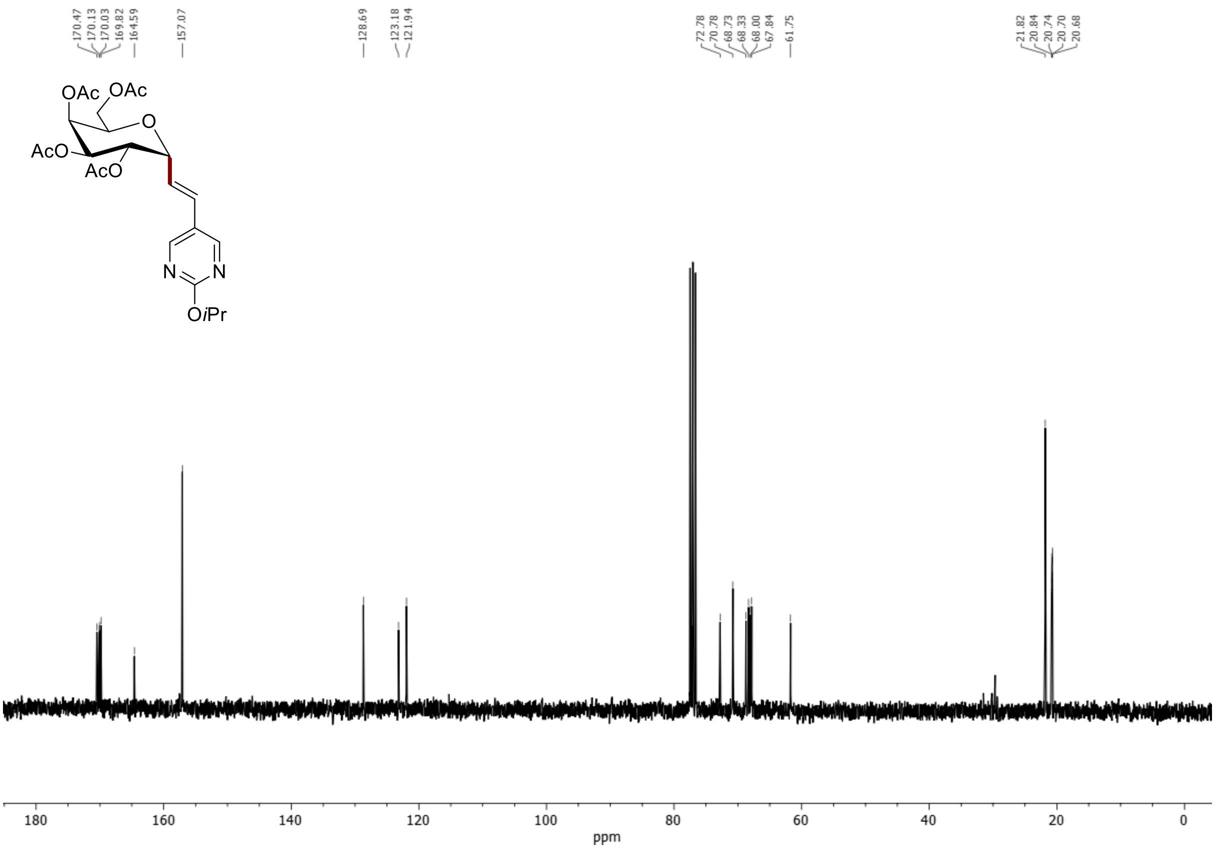

Compound **3j** ^{1}H-NMR (300 MHz, $CDCl_3$)

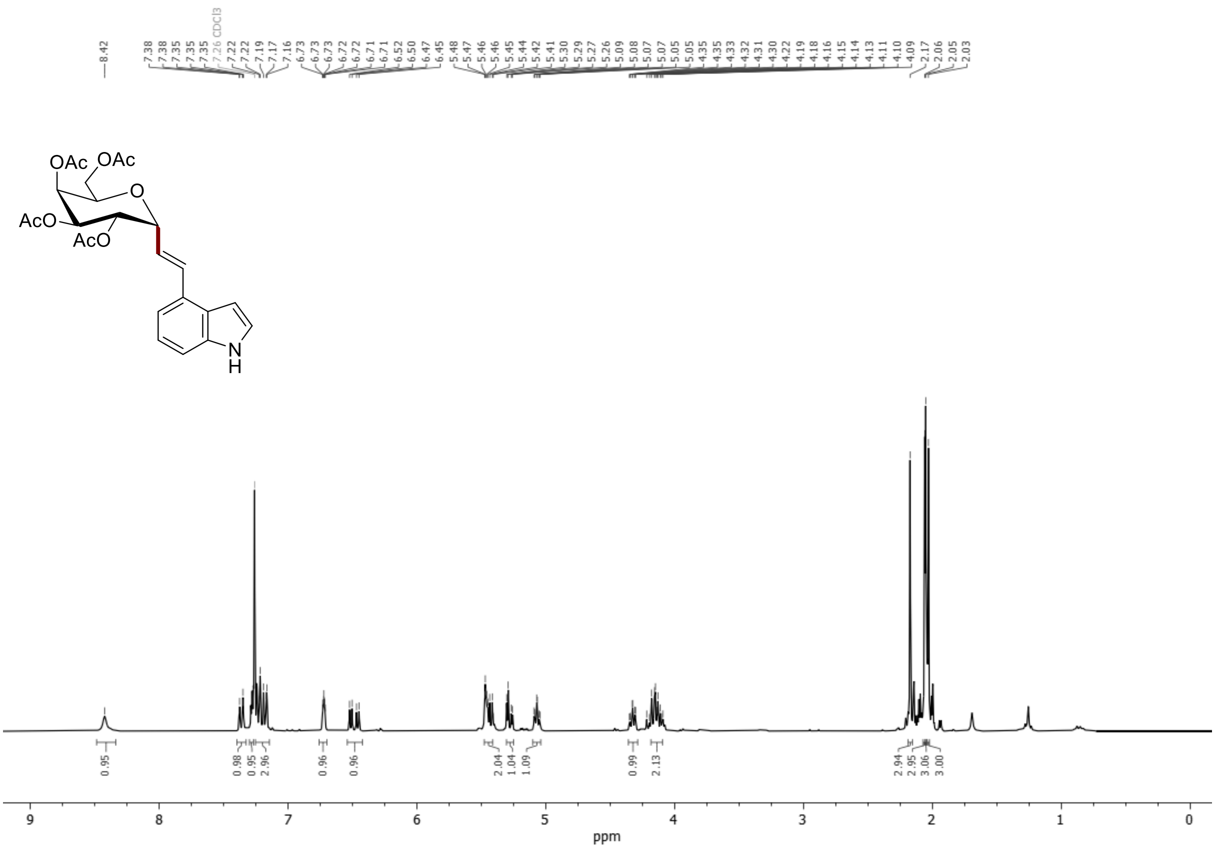

Compound **3j** ^{13}C{^{1}H}-NMR (75 MHz, $CDCl_3$)

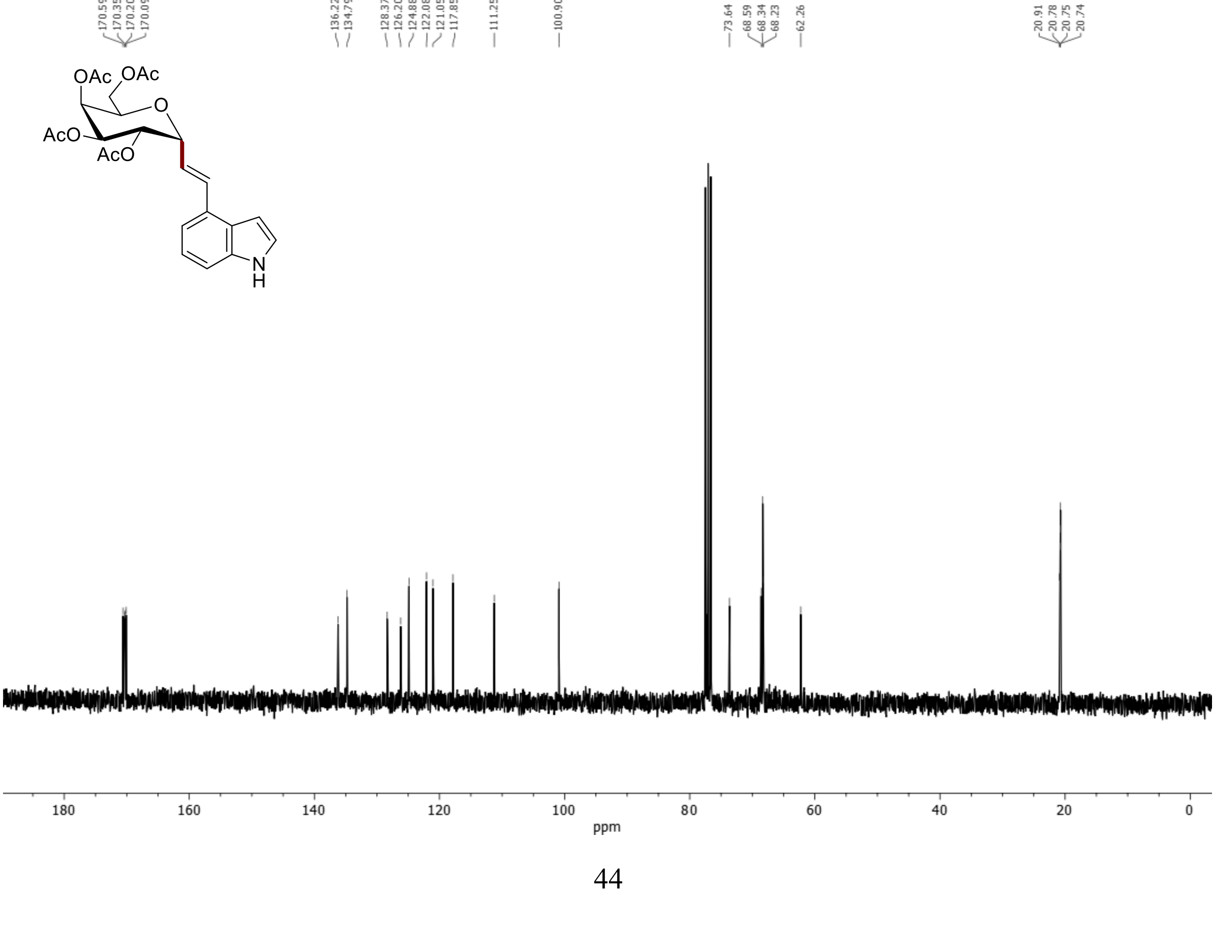

Compound **3k** ^{1}H-NMR (400 MHz, $CDCl_3$)

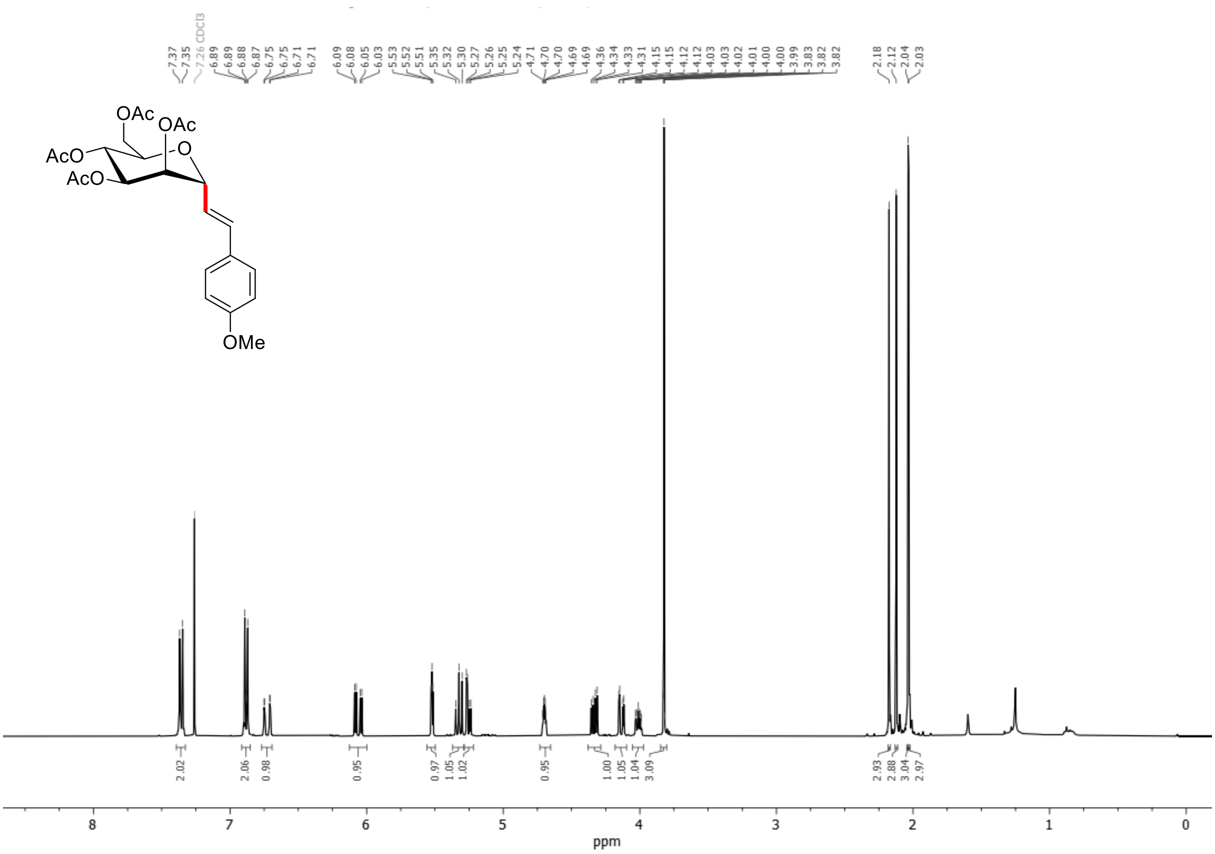

Compound **3k** $^{13}C\{^{1}H\}$-NMR (101 MHz, $CDCl_3$)

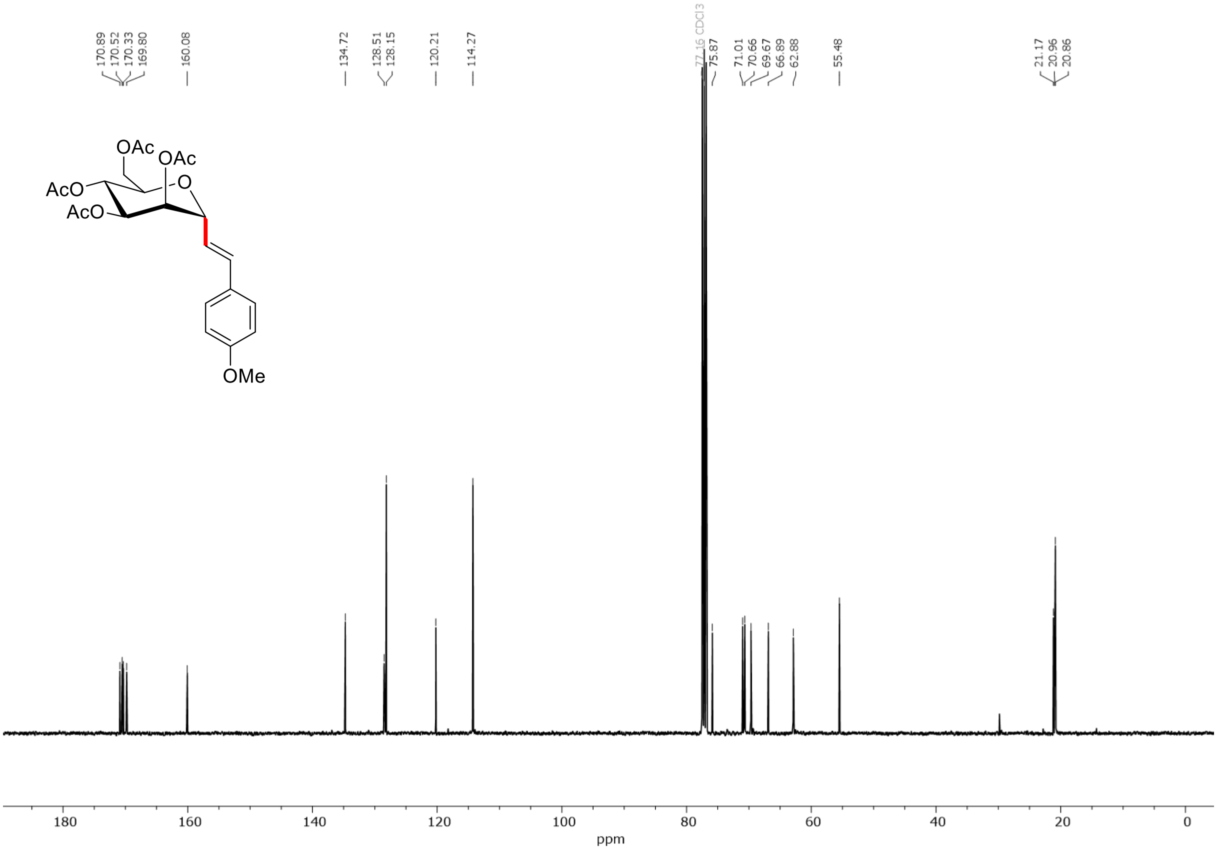

Compound **3l** ^{1}H-NMR (400 MHz, $CDCl_3$)

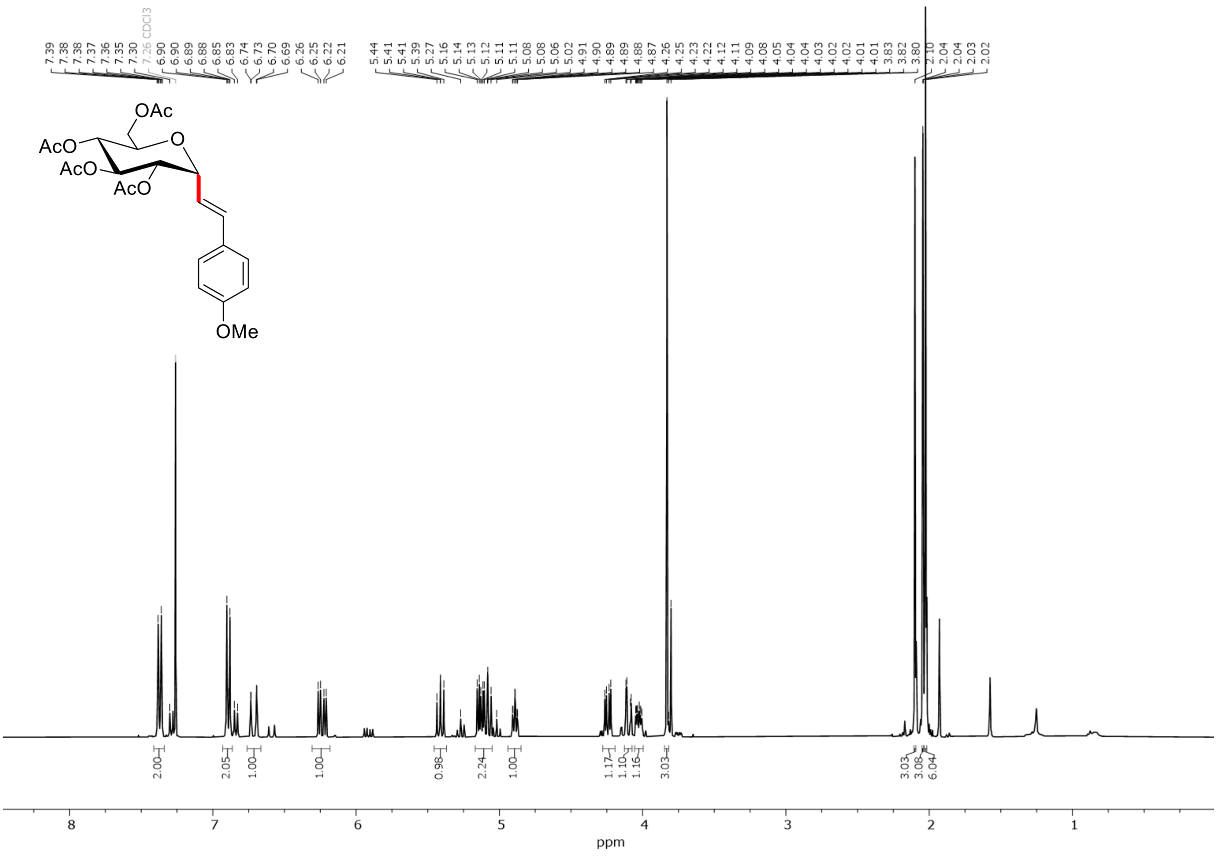

Compound **3l** ^{13}C{^{1}H}-NMR (101 MHz, $CDCl_3$)

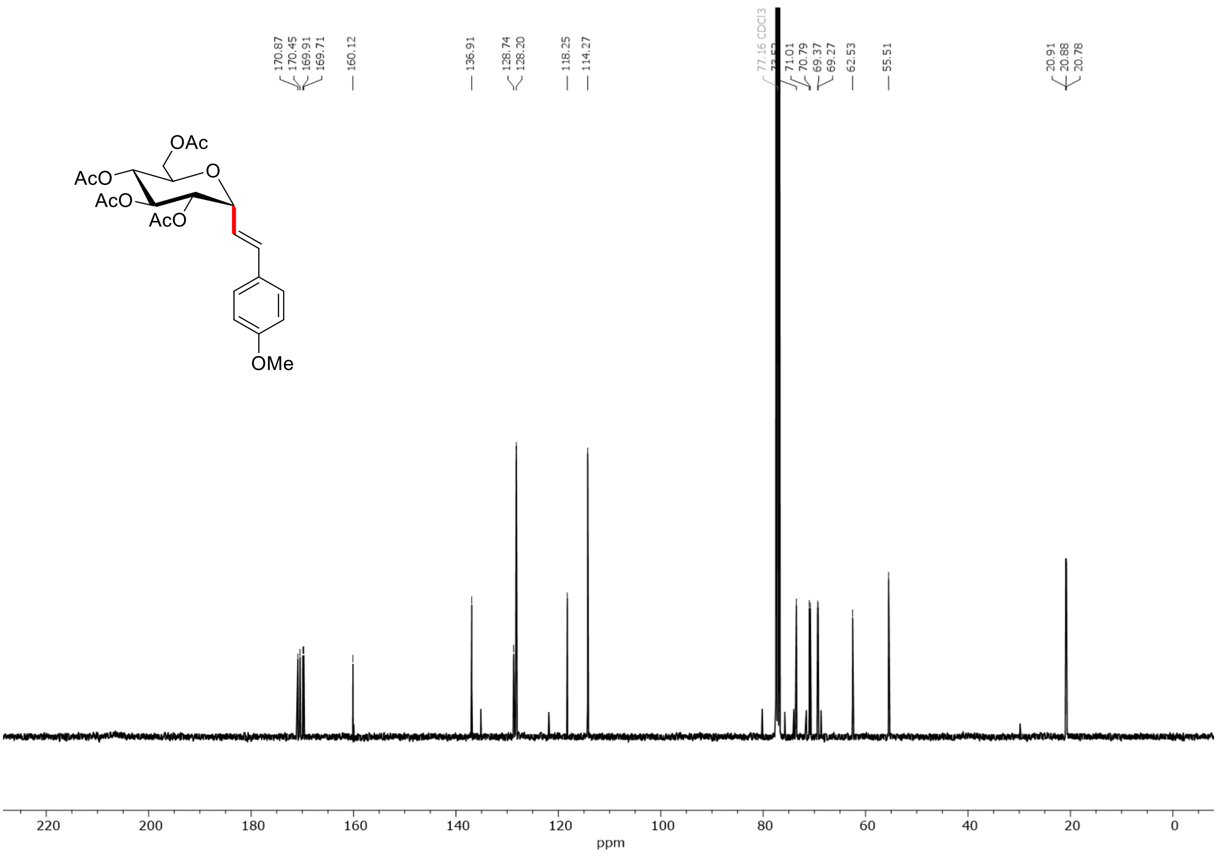

Compound **3m** ^{1}H-NMR (400 MHz, $CDCl_3$)

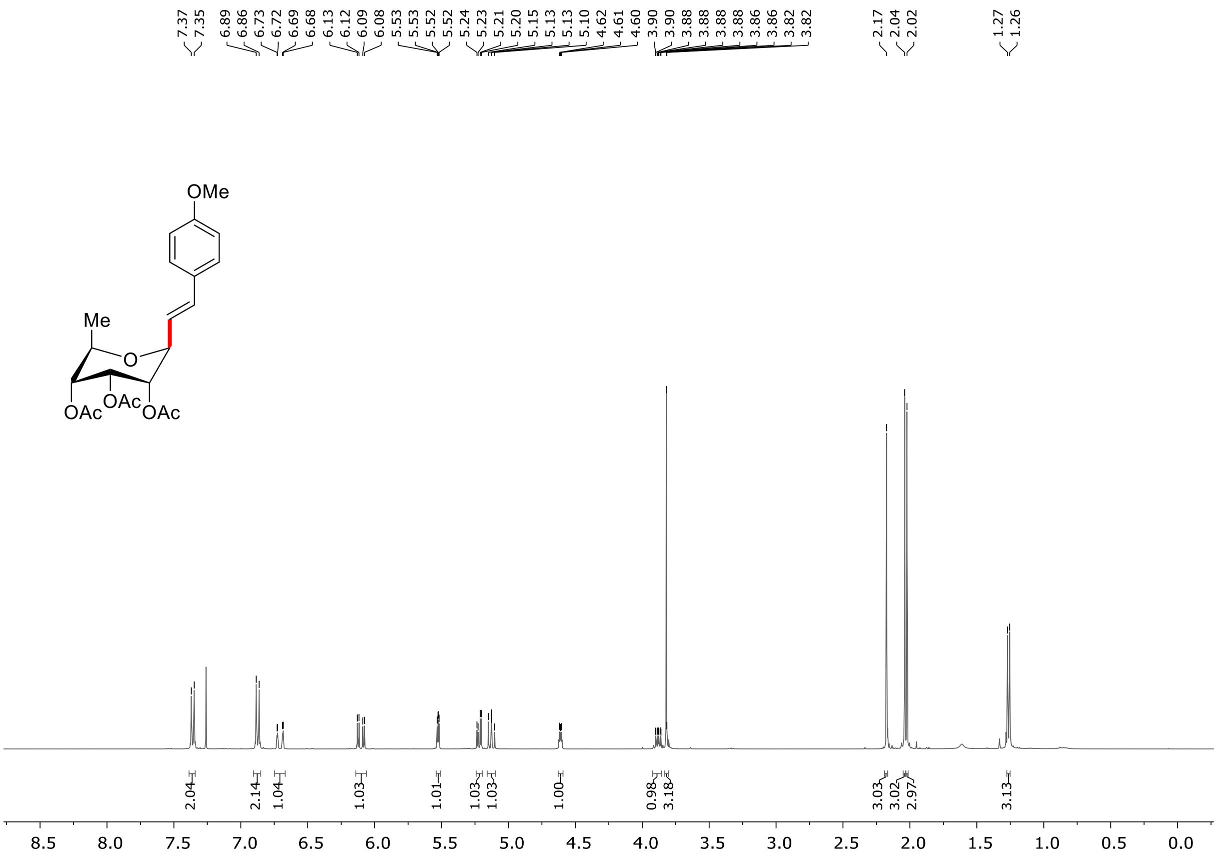

Compound **3m** $^{13}C\{^{1}H\}$-NMR (101 MHz, $CDCl_3$)

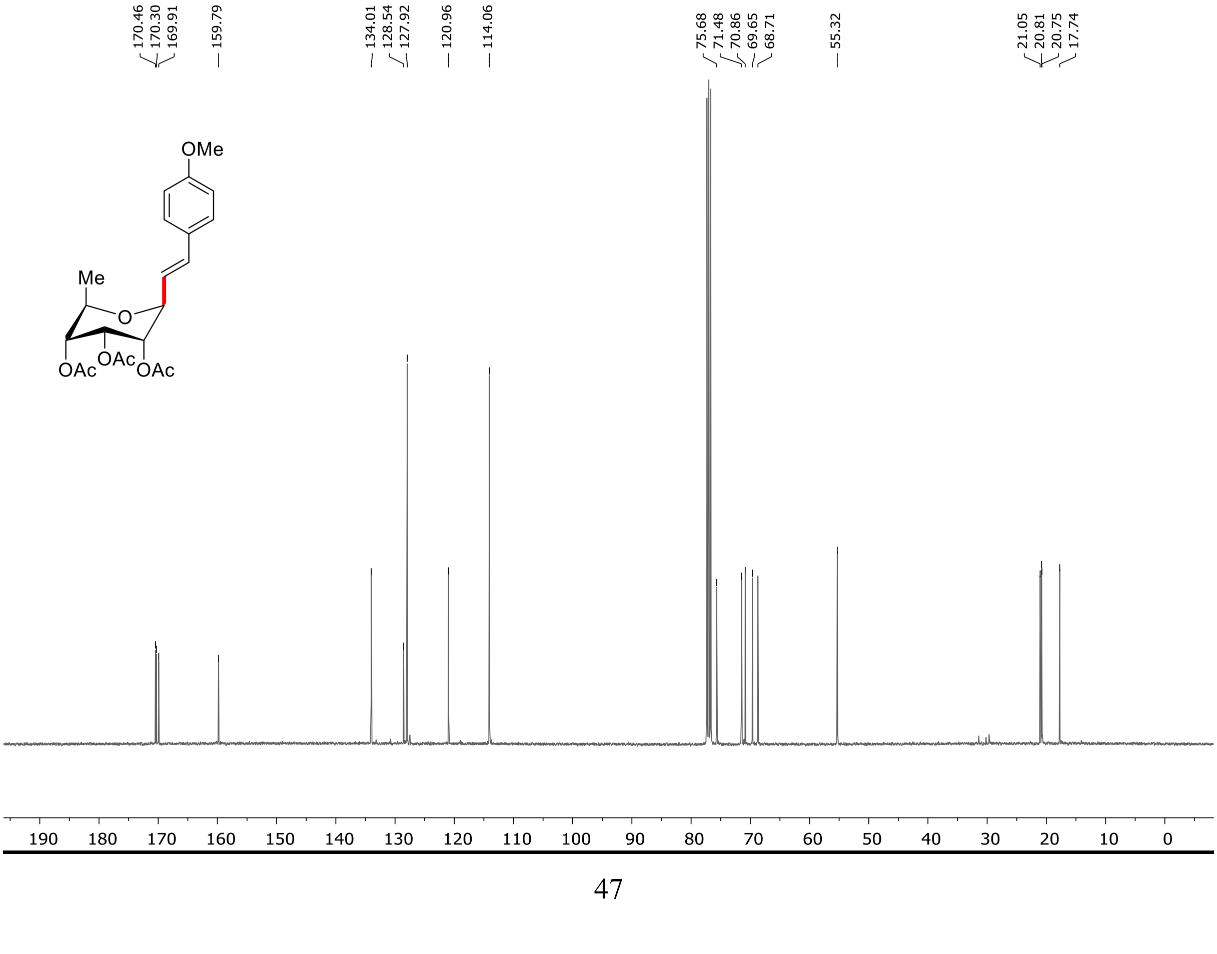

Compound **3n** ^{1}H-NMR (400 MHz, $CDCl_3$)

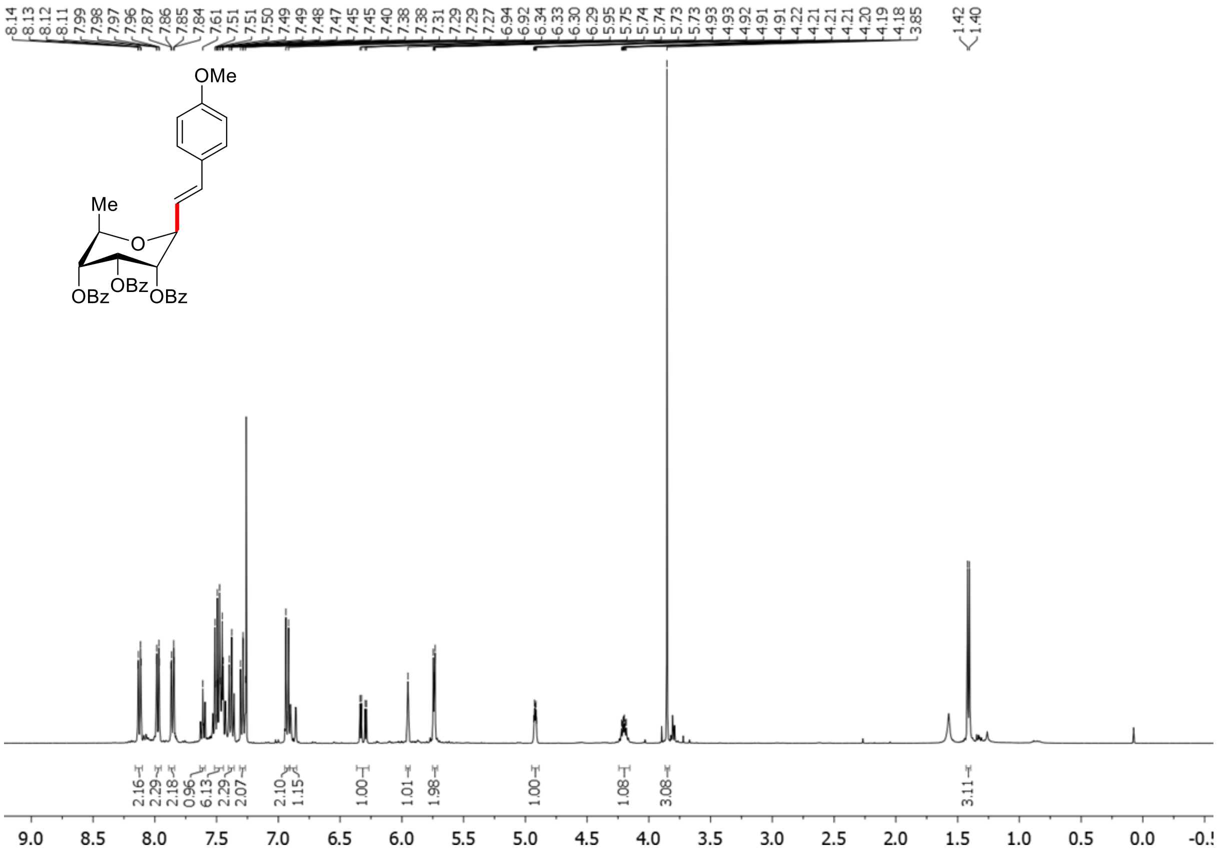

Compound **3n** ^{13}C{^{1}H}-NMR (101 MHz, $CDCl_3$)

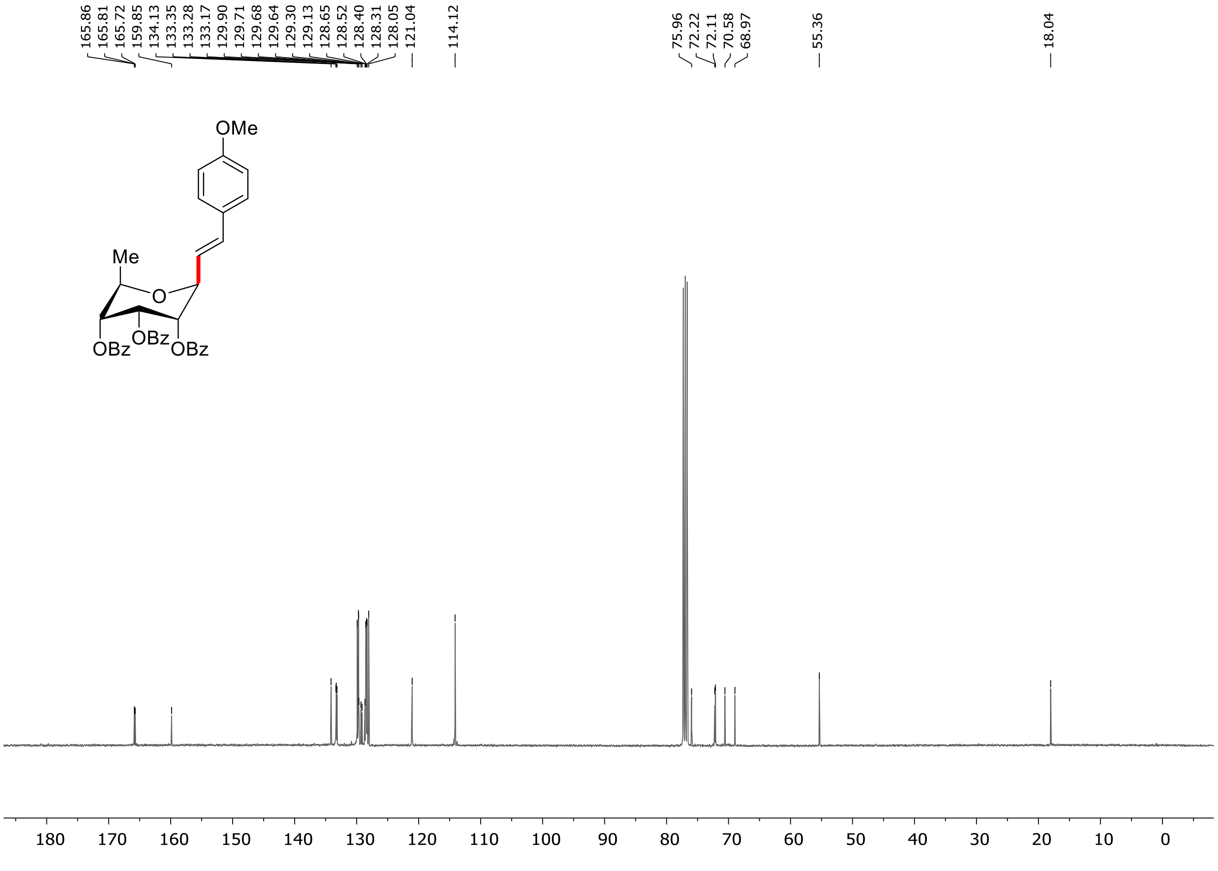

Compound **3o** ^{1}H-NMR (400 MHz, $CDCl_3$)

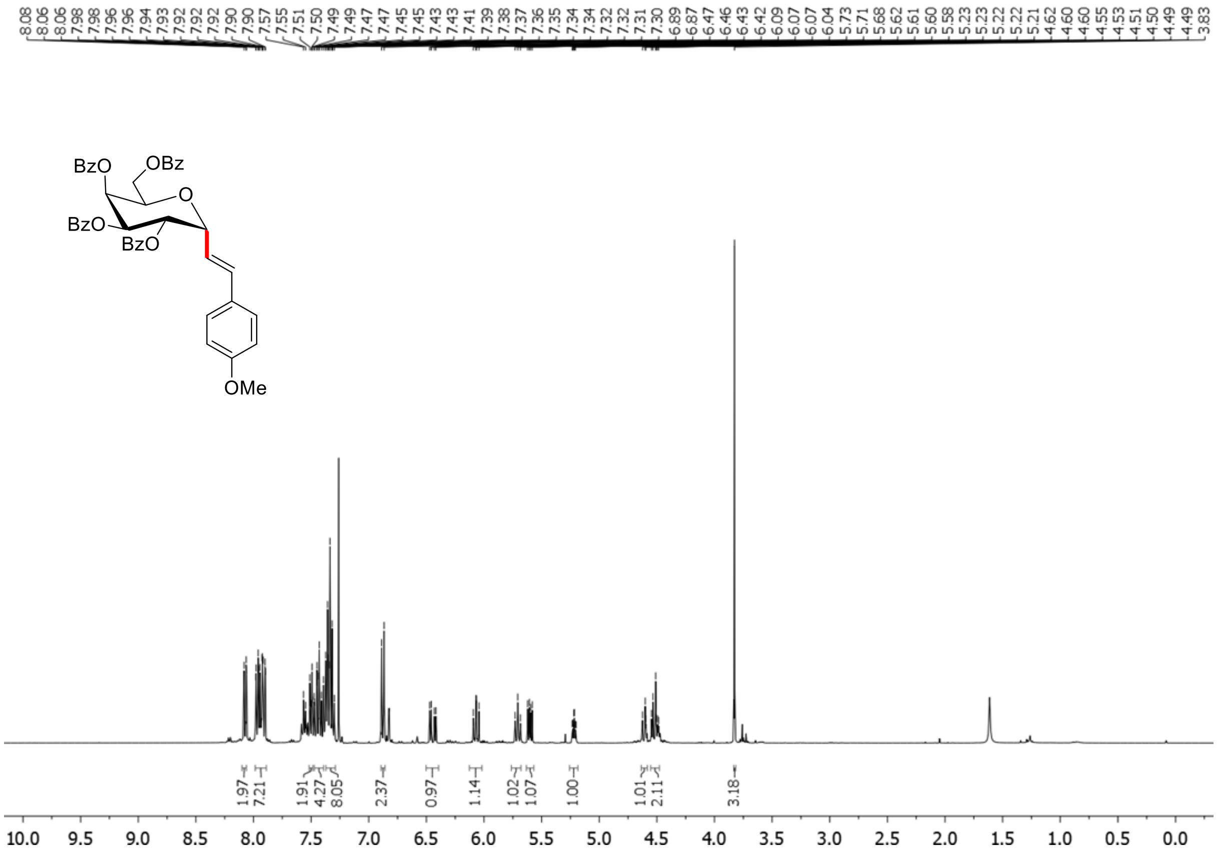

Compound **3o** $^{13}C\{^{1}H\}$-NMR (101 MHz, $CDCl_3$)

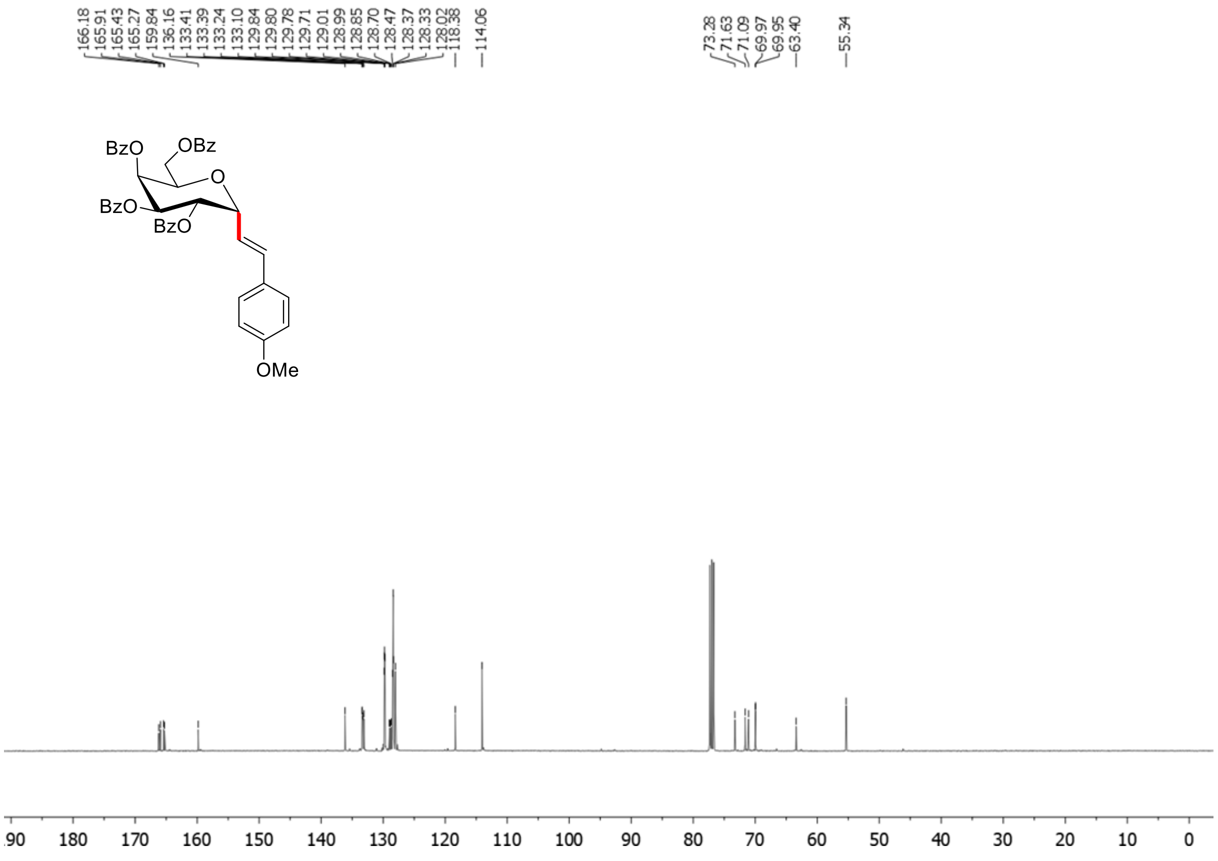

Compound **3p** ^{1}H-NMR (400 MHz, $CDCl_3$)

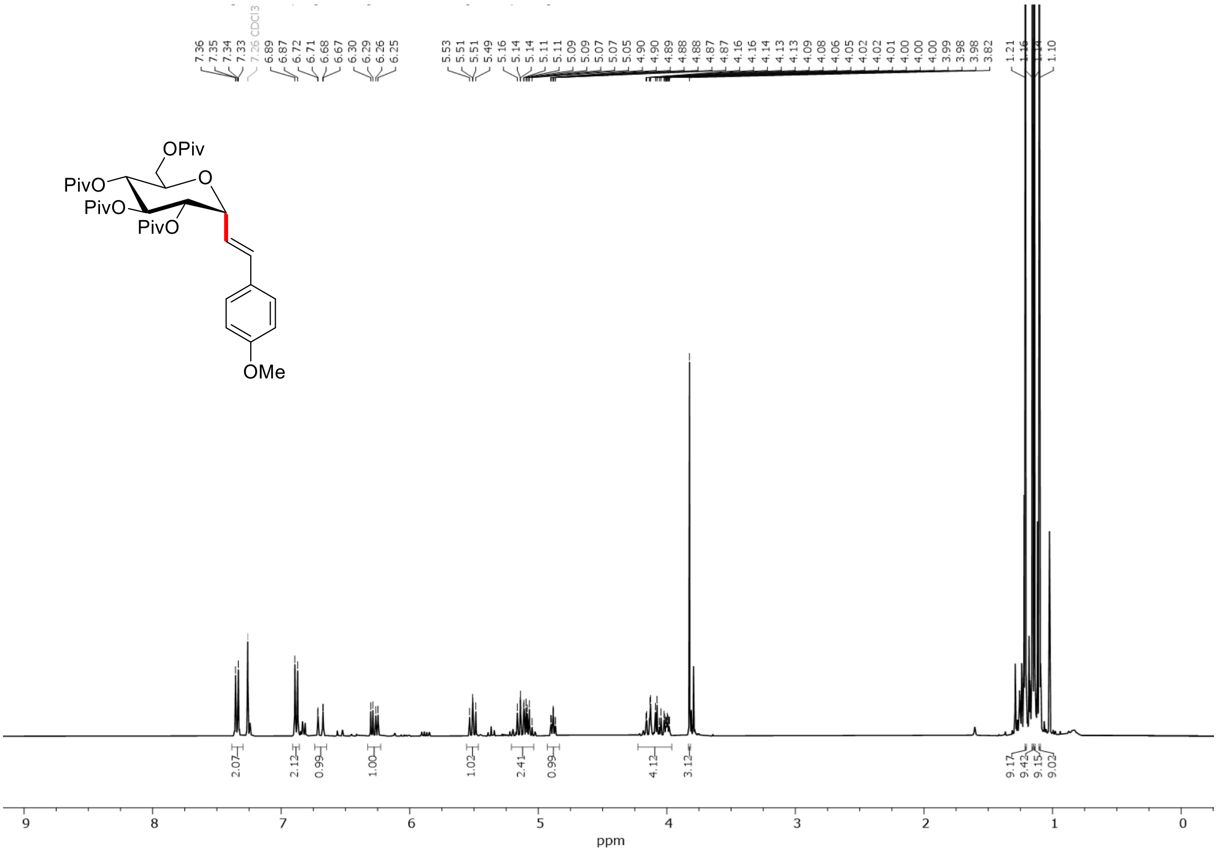

Compound **3p** $^{13}C\{^{1}H\}$-NMR (101 MHz, $CDCl_3$)

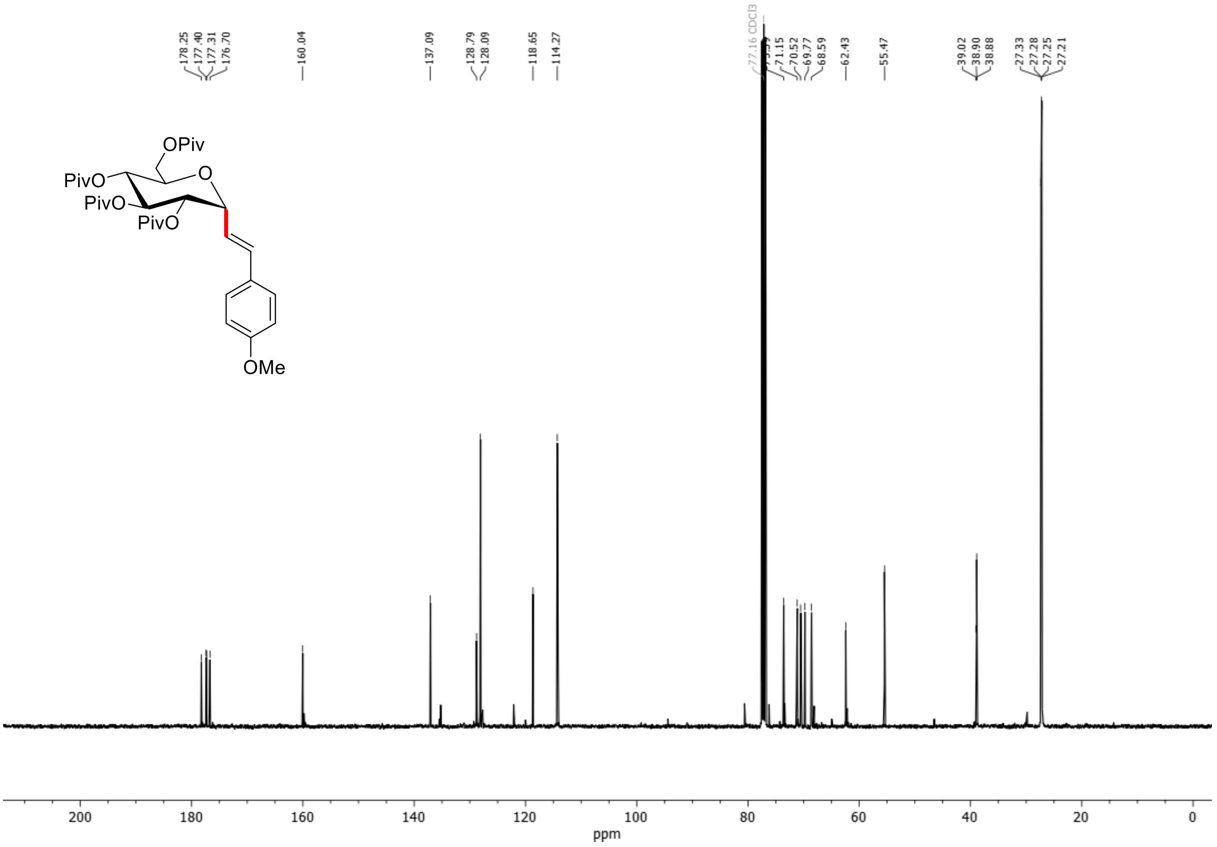

Compound **3q** ^{1}H-NMR (400 MHz, $CDCl_3$)

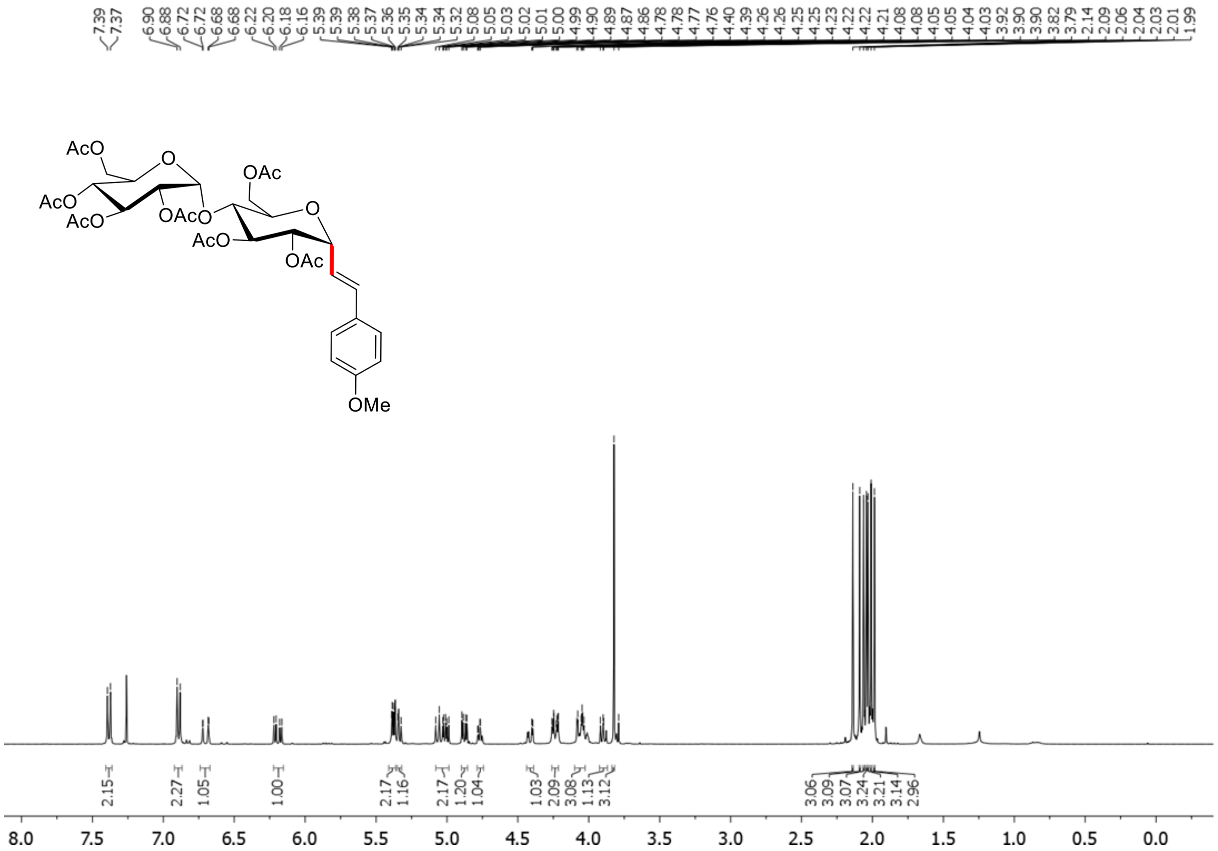

Compound **3q** ^{13}C{^{1}H}-NMR (101 MHz, $CDCl_3$)

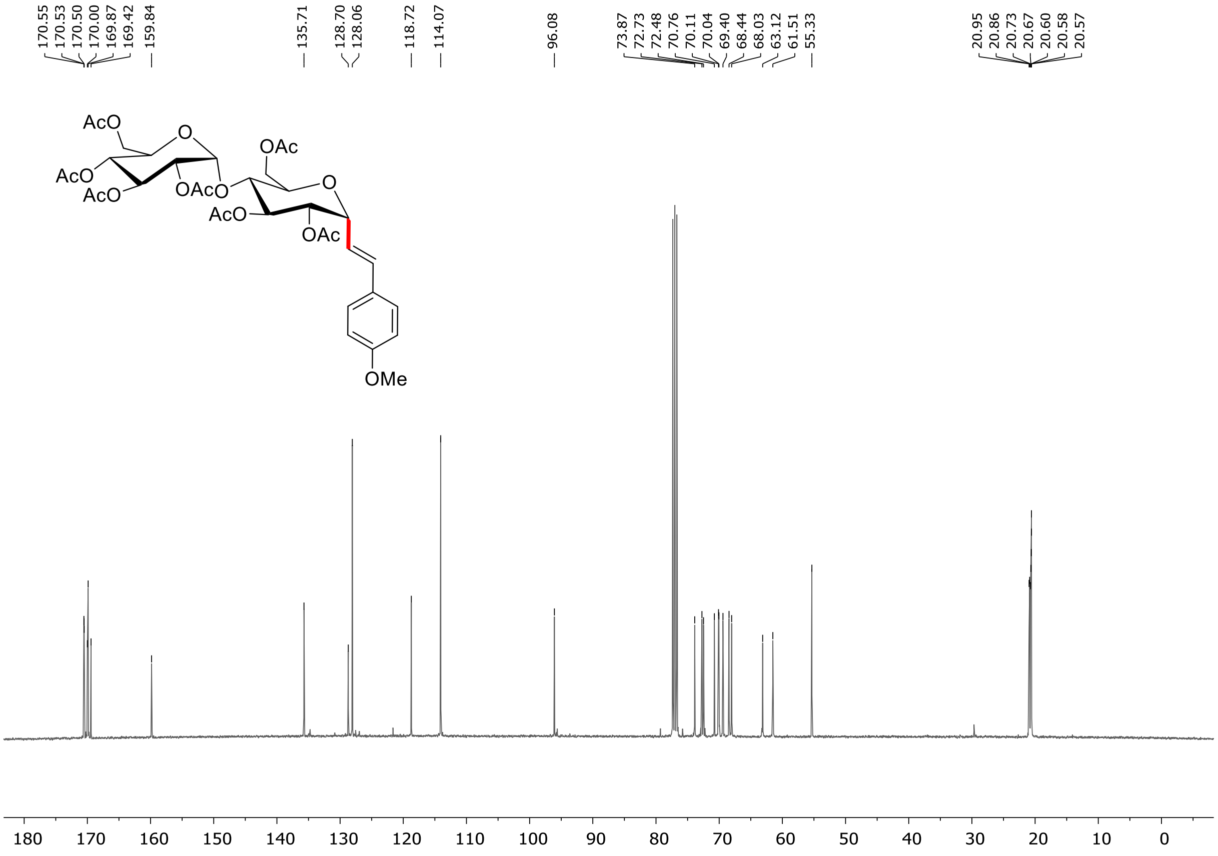

Compound **3r** ^{1}H-NMR (400 MHz, $CDCl_3$)

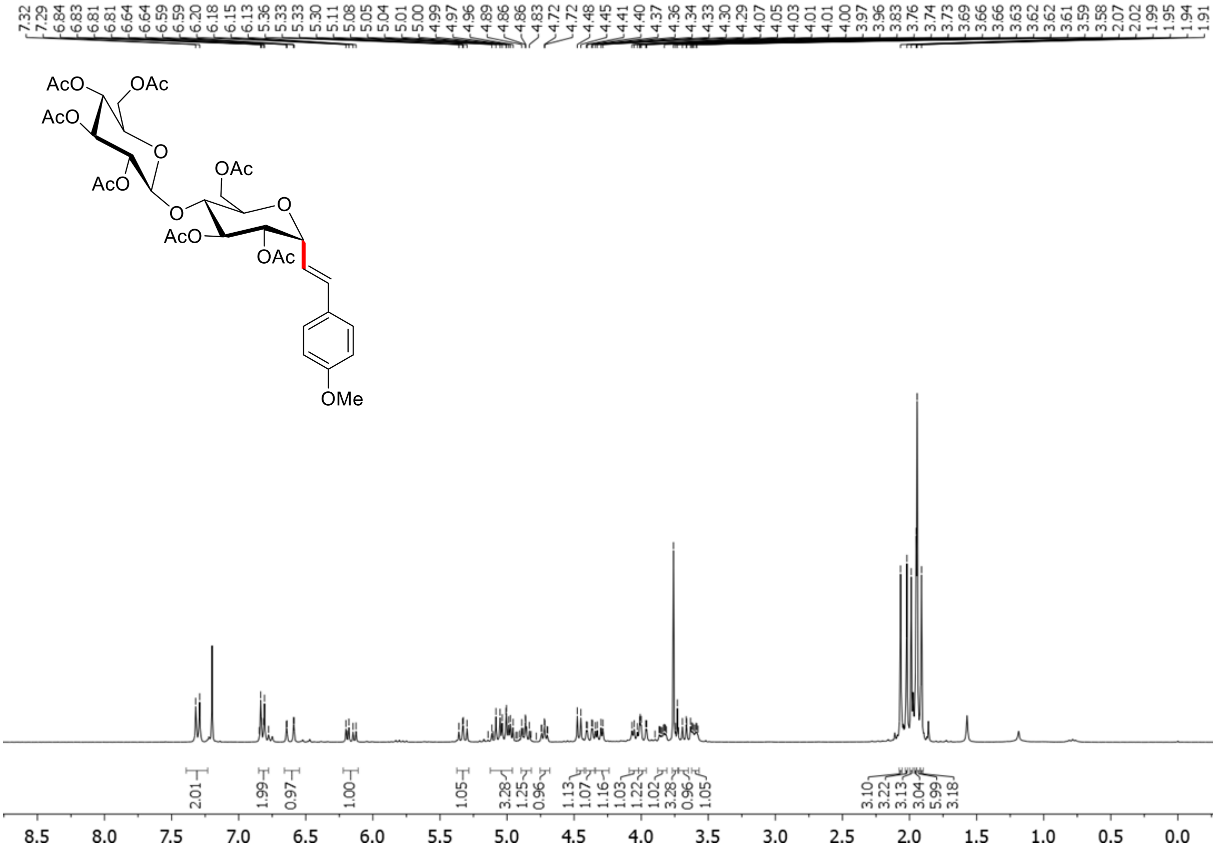

Compound **3r** $^{13}C\{^{1}H\}$-NMR (101 MHz, $CDCl_3$)

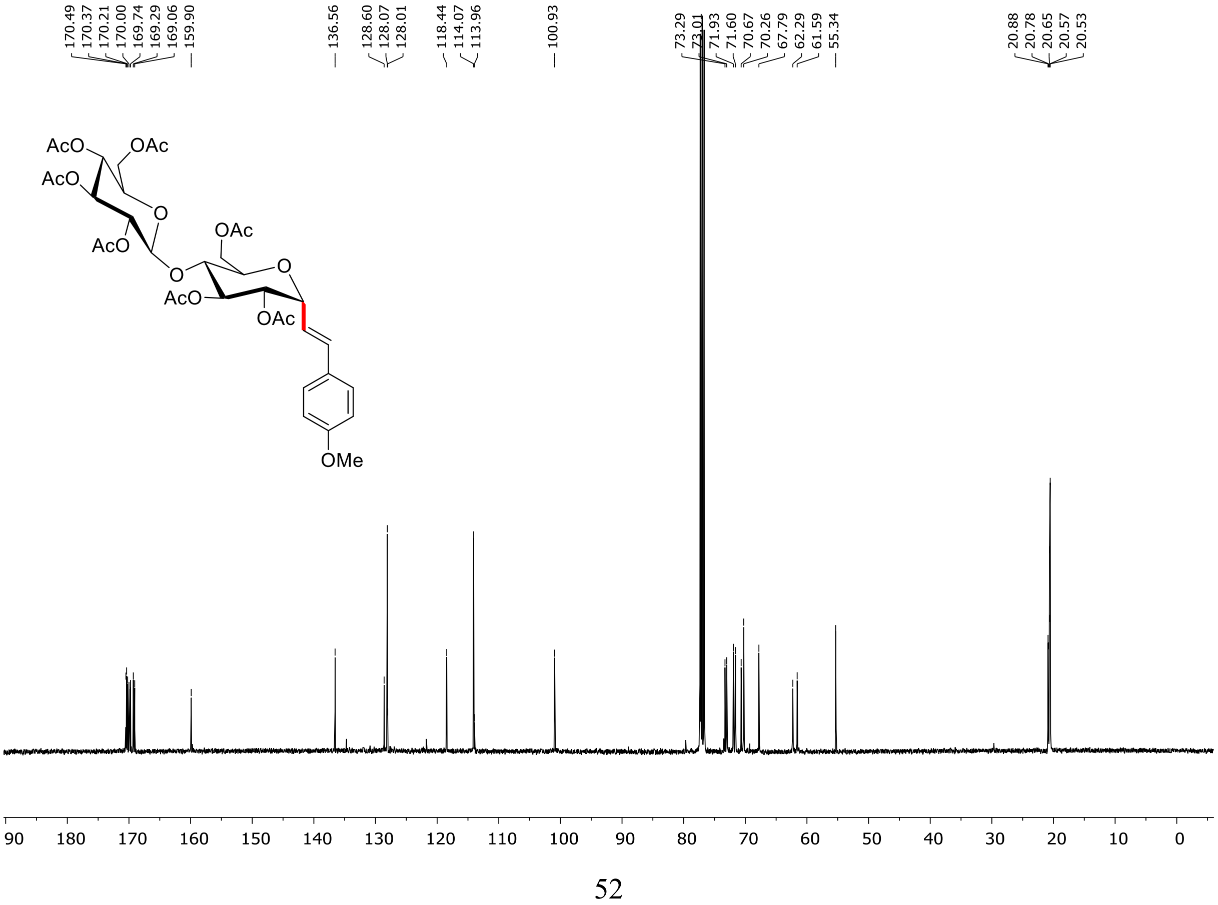

Compound **3s** ^{1}H-NMR (400 MHz, $CDCl_3$)

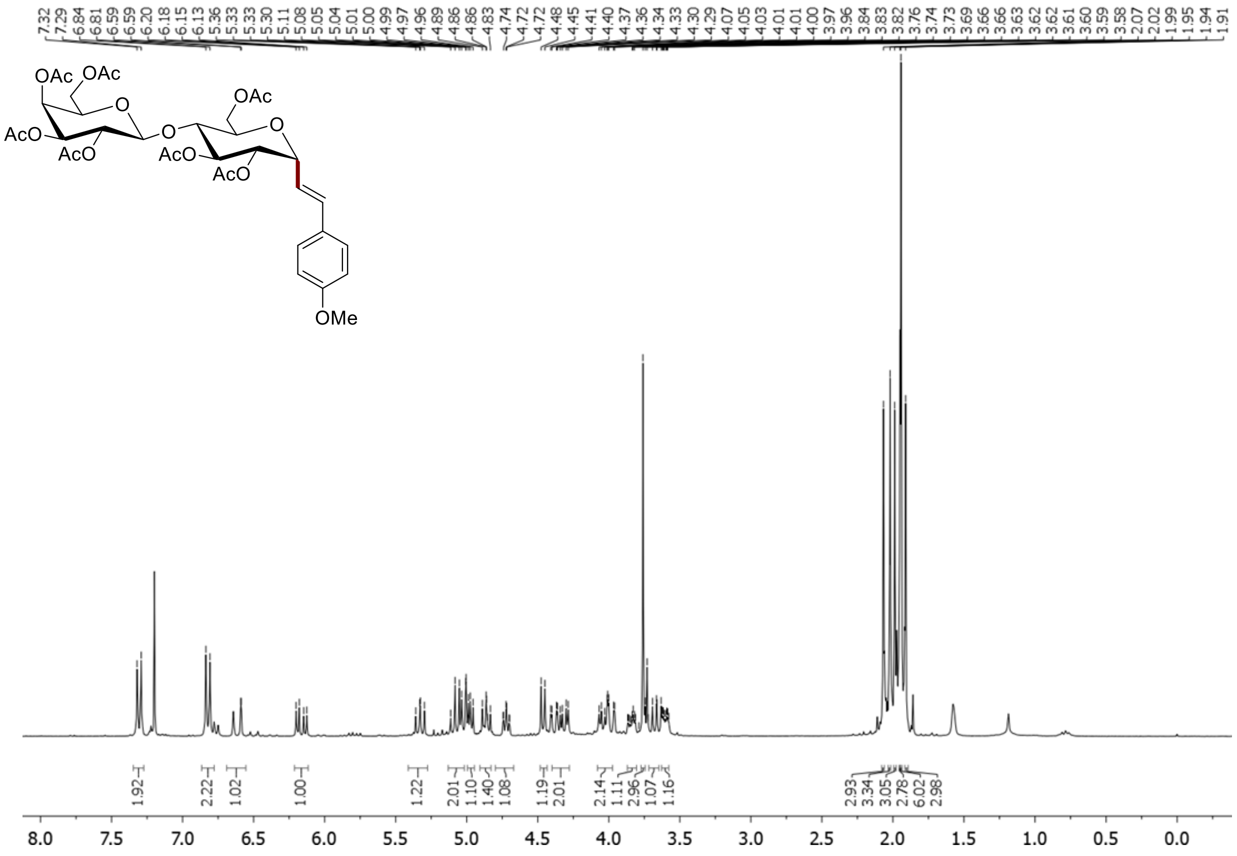

Compound **3s** $^{13}C\{^{1}H\}$-NMR (101 MHz, $CDCl_3$)

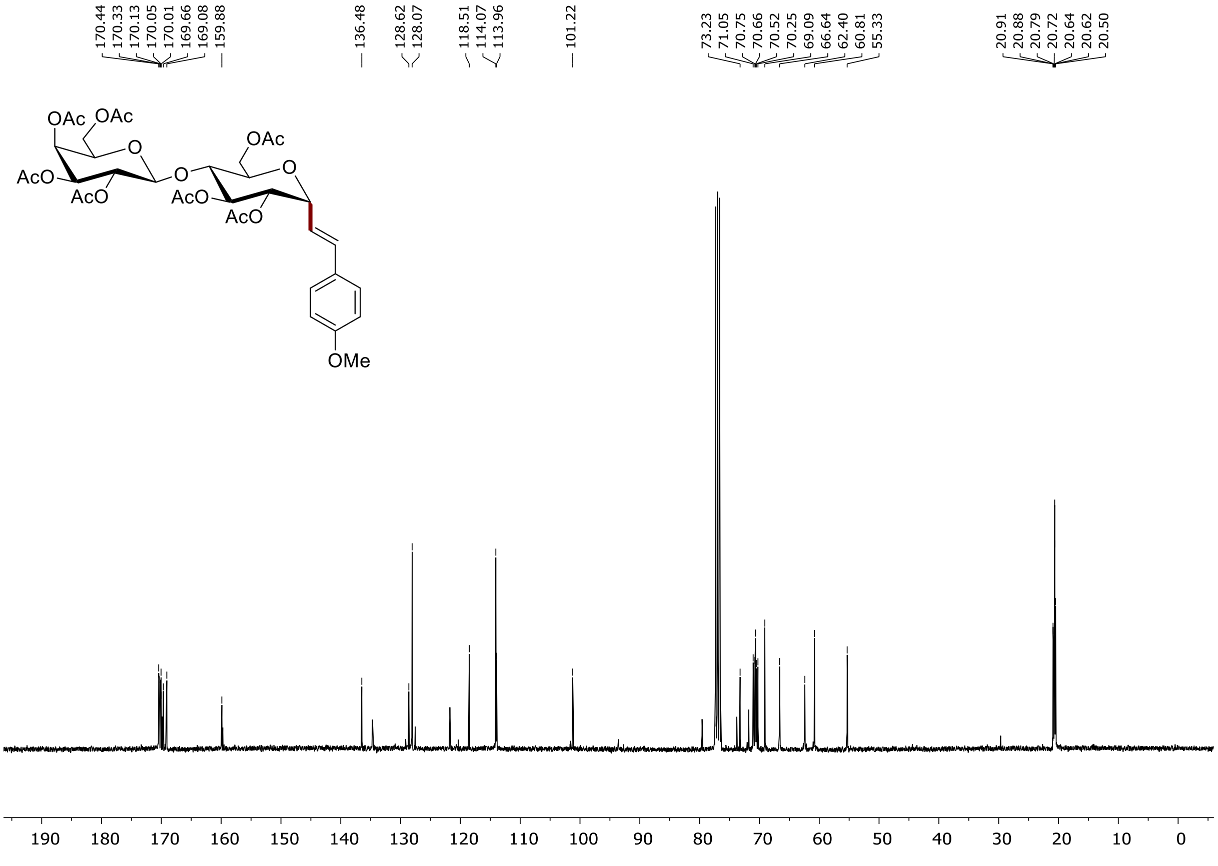

Compound **3t** ^{1}H-NMR (400 MHz, CDCl$_3$)

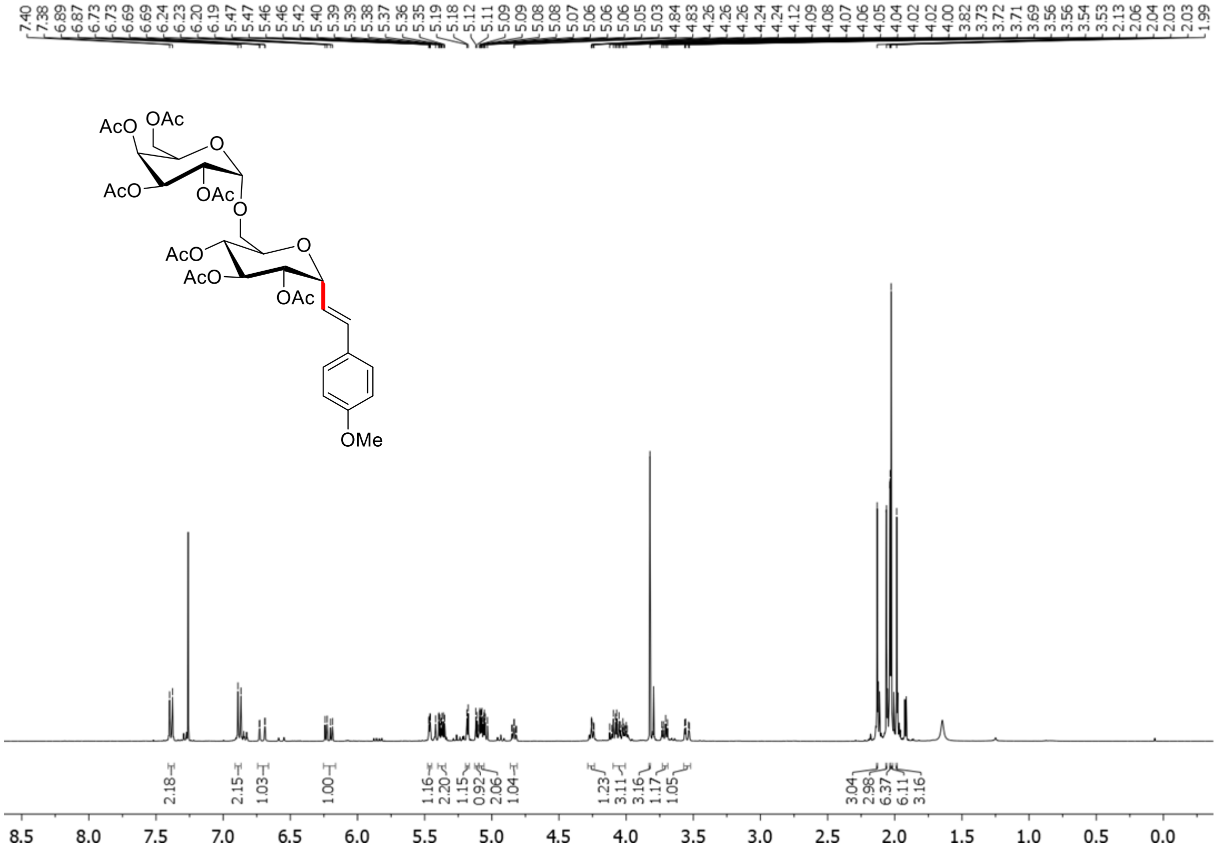

Compound **3t** ^{13}C{^{1}H}-NMR (101 MHz, CDCl$_3$)

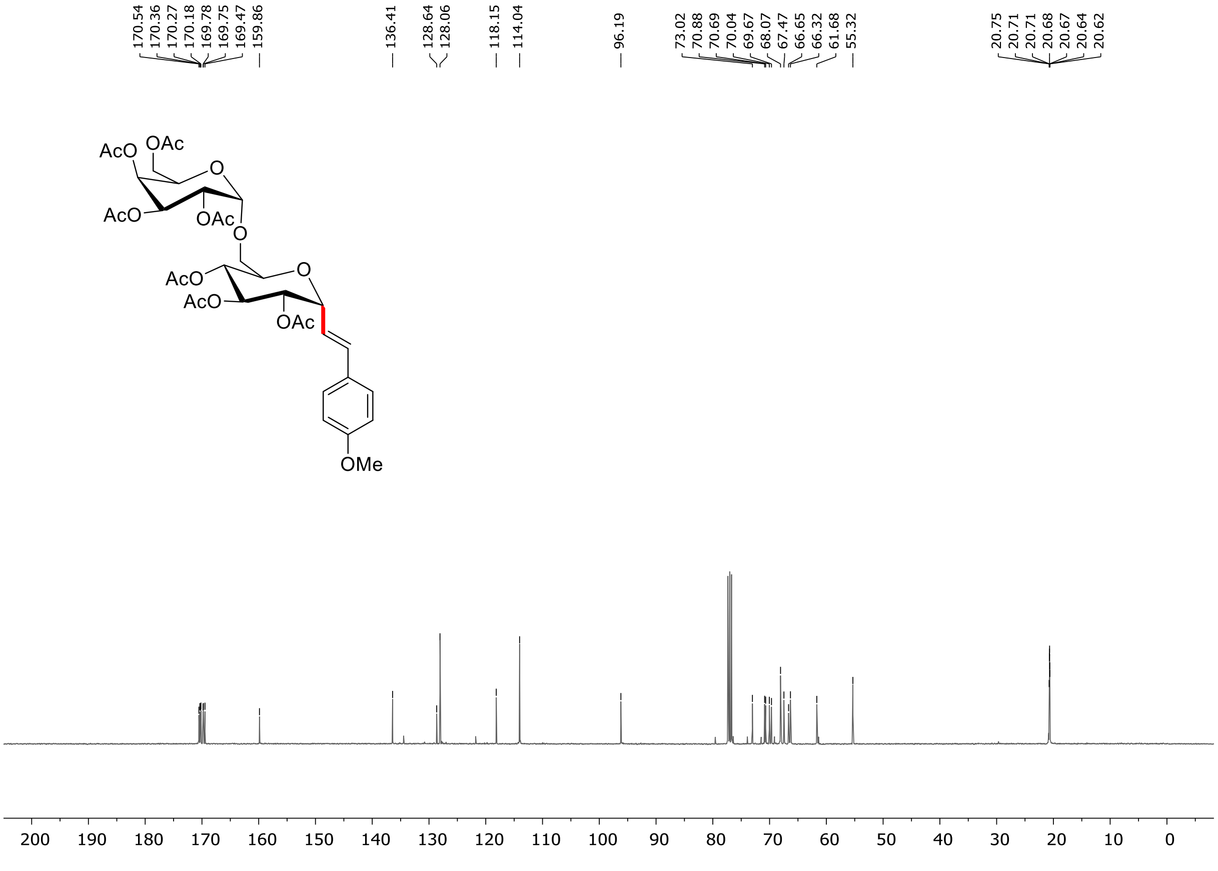

Compound **3u** ^{1}H-NMR (400 MHz, $CDCl_3$)

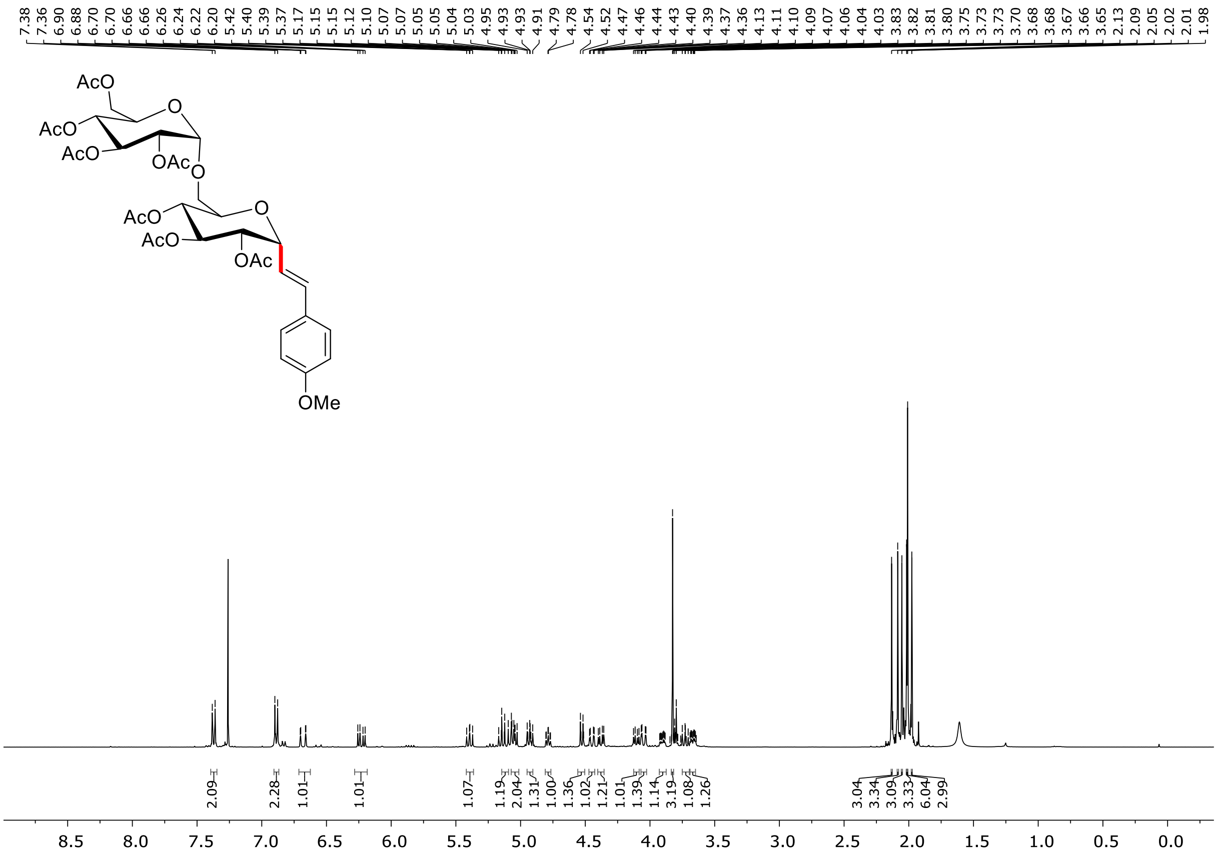

Compound **3u** ^{13}C{^{1}H}-NMR (101 MHz, $CDCl_3$)

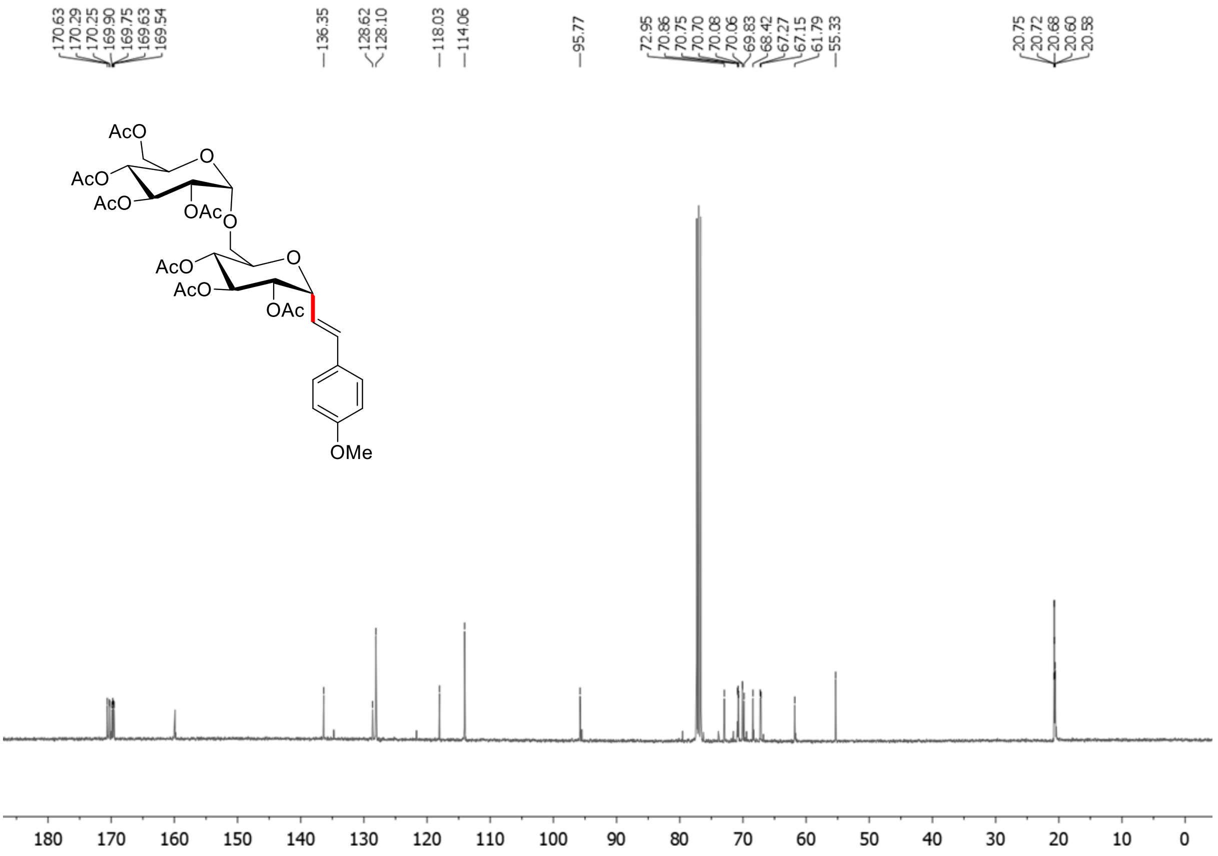

Compound **3v** ^{1}H-NMR (400 MHz, $CDCl_3$)

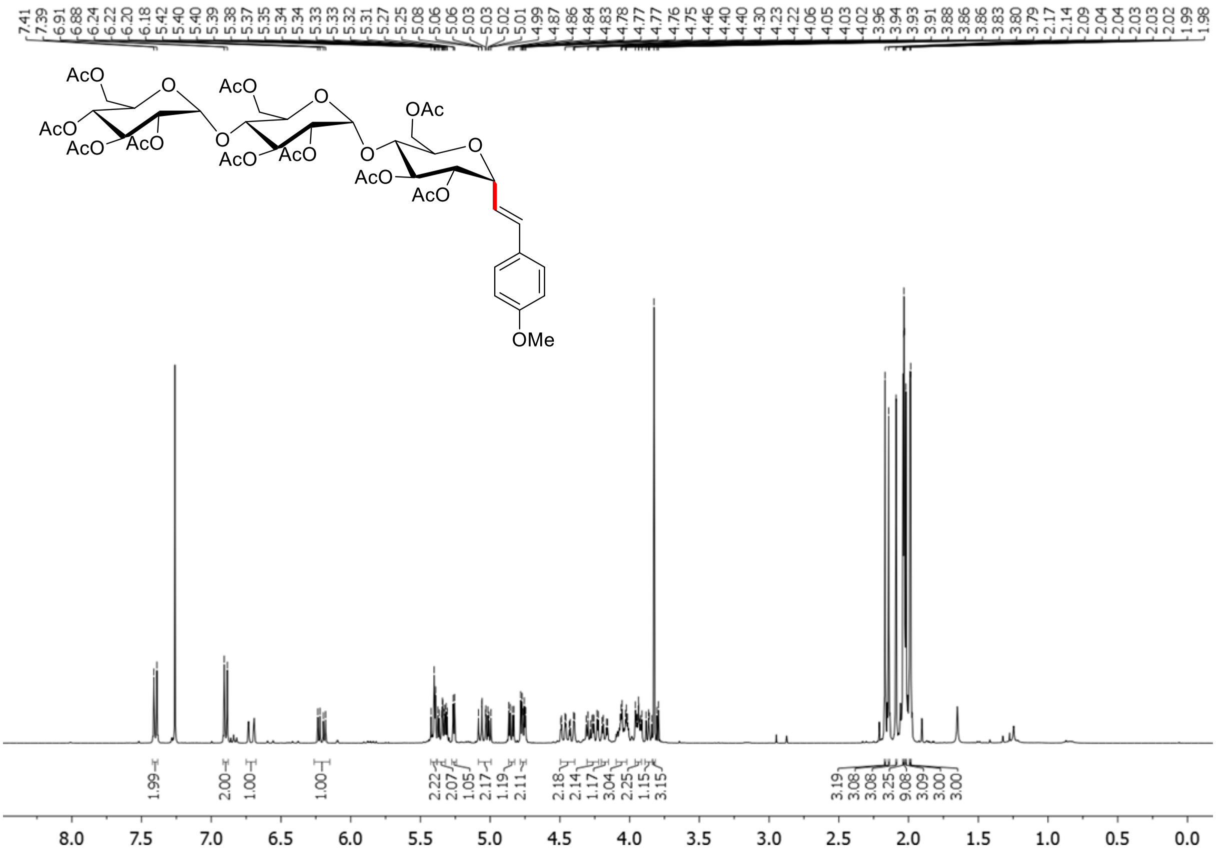

Compound **3v** ^{13}C{^{1}H}-NMR (101 MHz, $CDCl_3$)

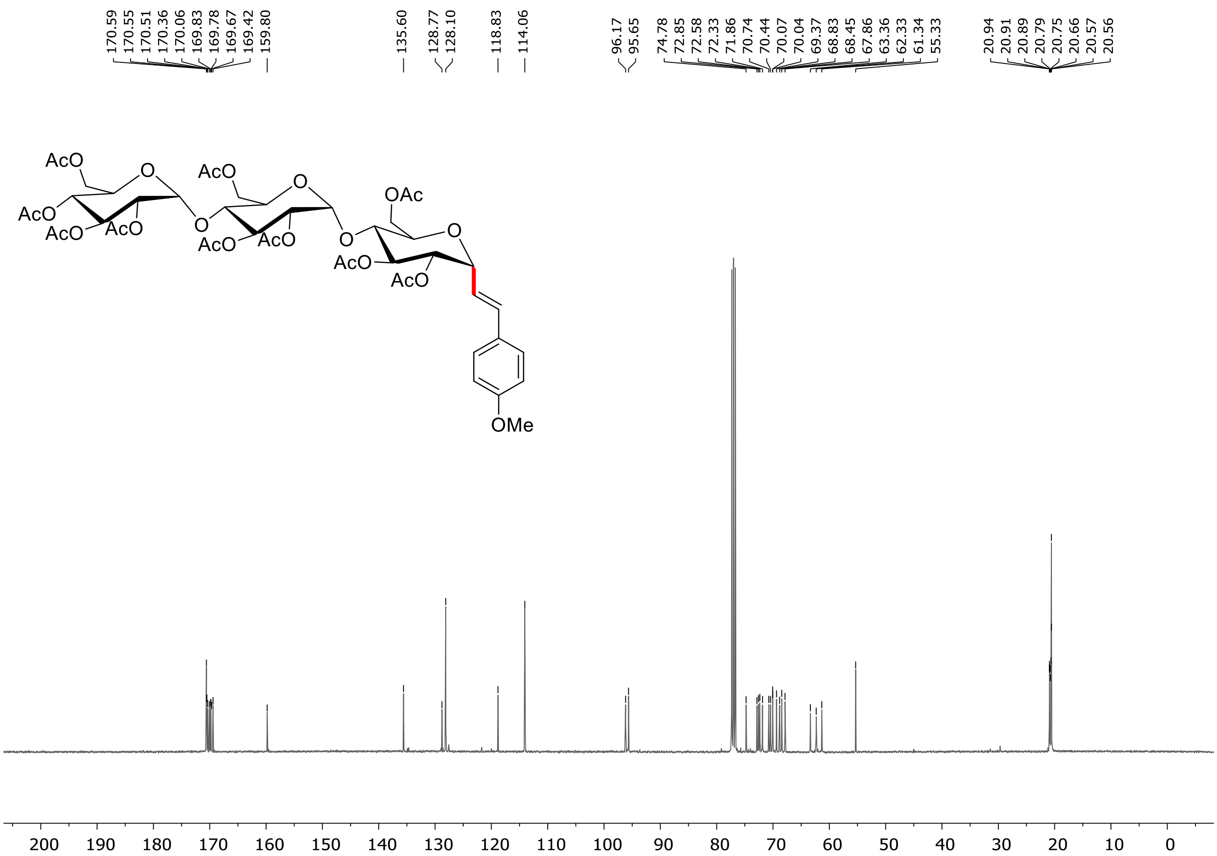

Compound **3w** ^{1}H-NMR (400 MHz, $CDCl_3$)

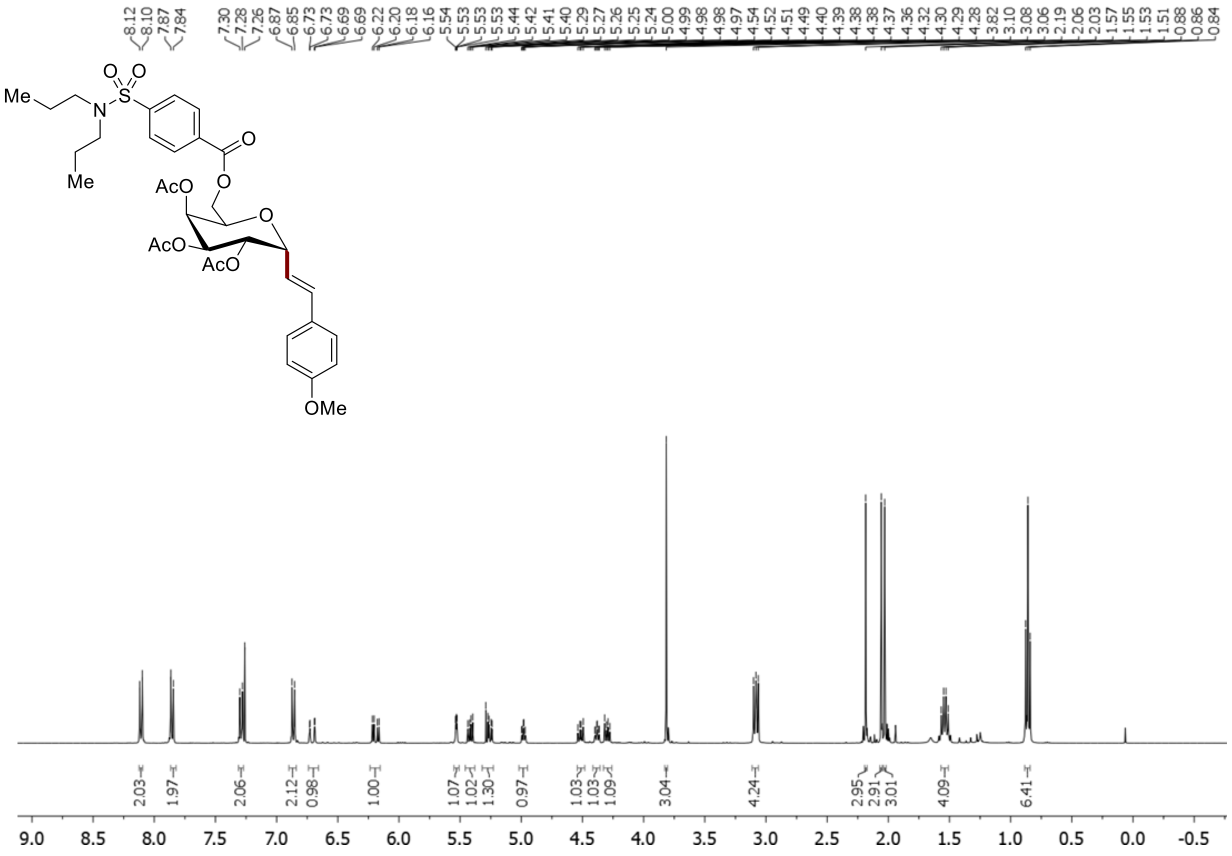

Compound **3w** $^{13}C\{^1H\}$-NMR (101 MHz, $CDCl_3$)

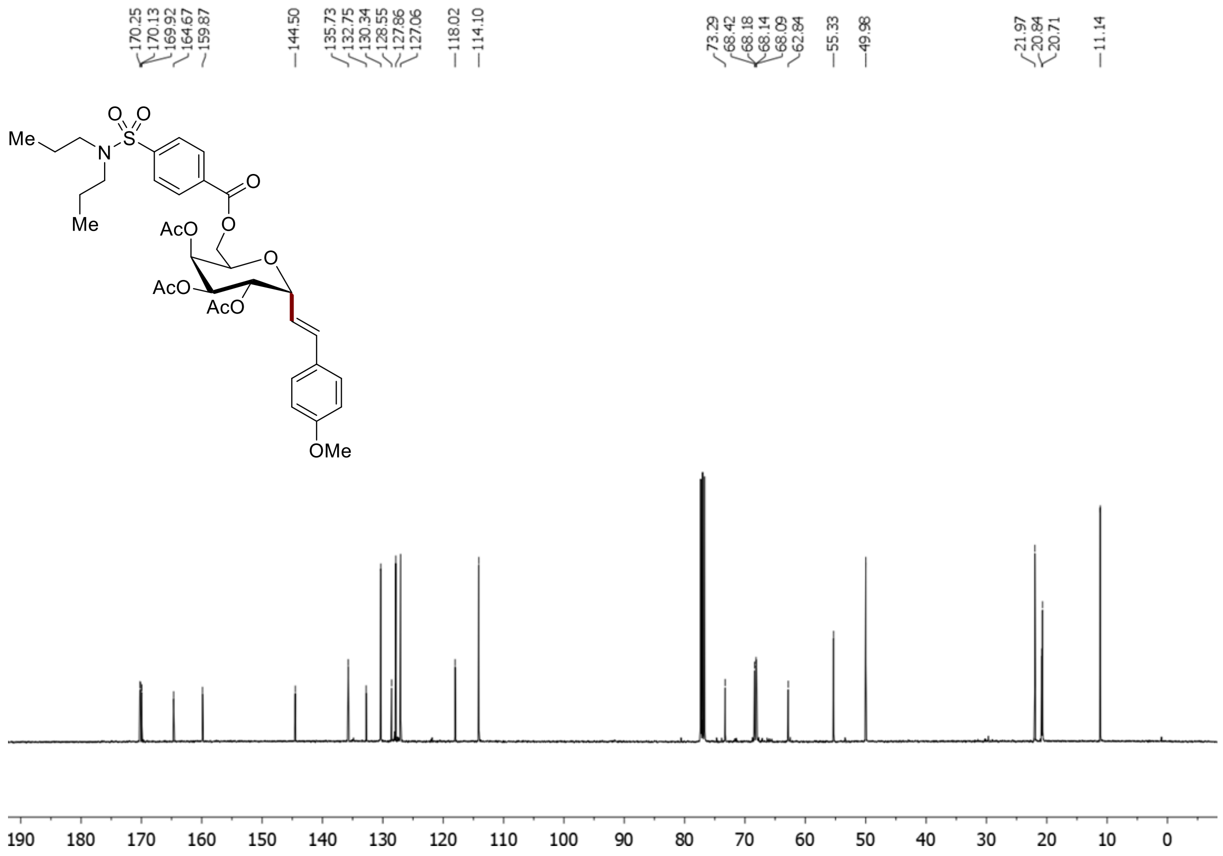

Compound **3x** ^{1}H-NMR (400 MHz, $CDCl_3$)

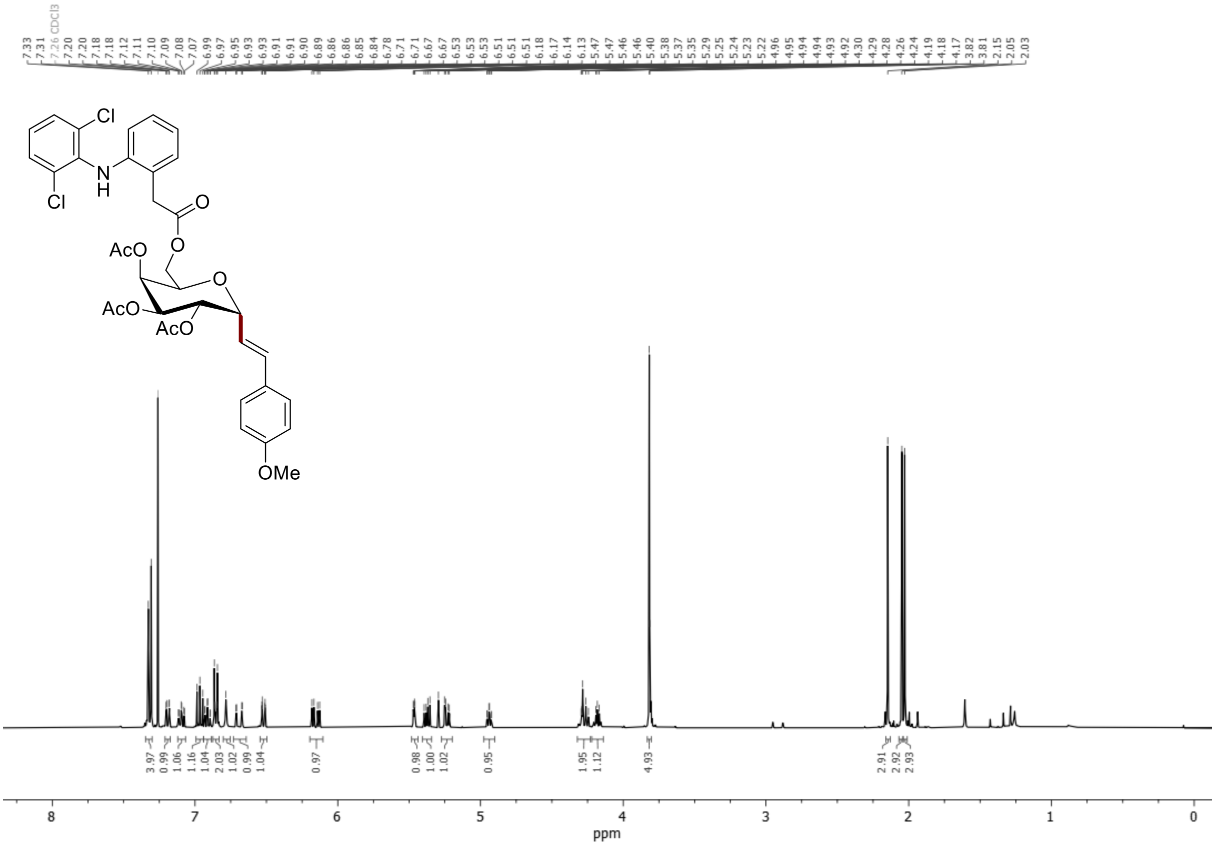

Compound **3x** $^{13}C\{^{1}H\}$-NMR (101 MHz, $CDCl_3$)

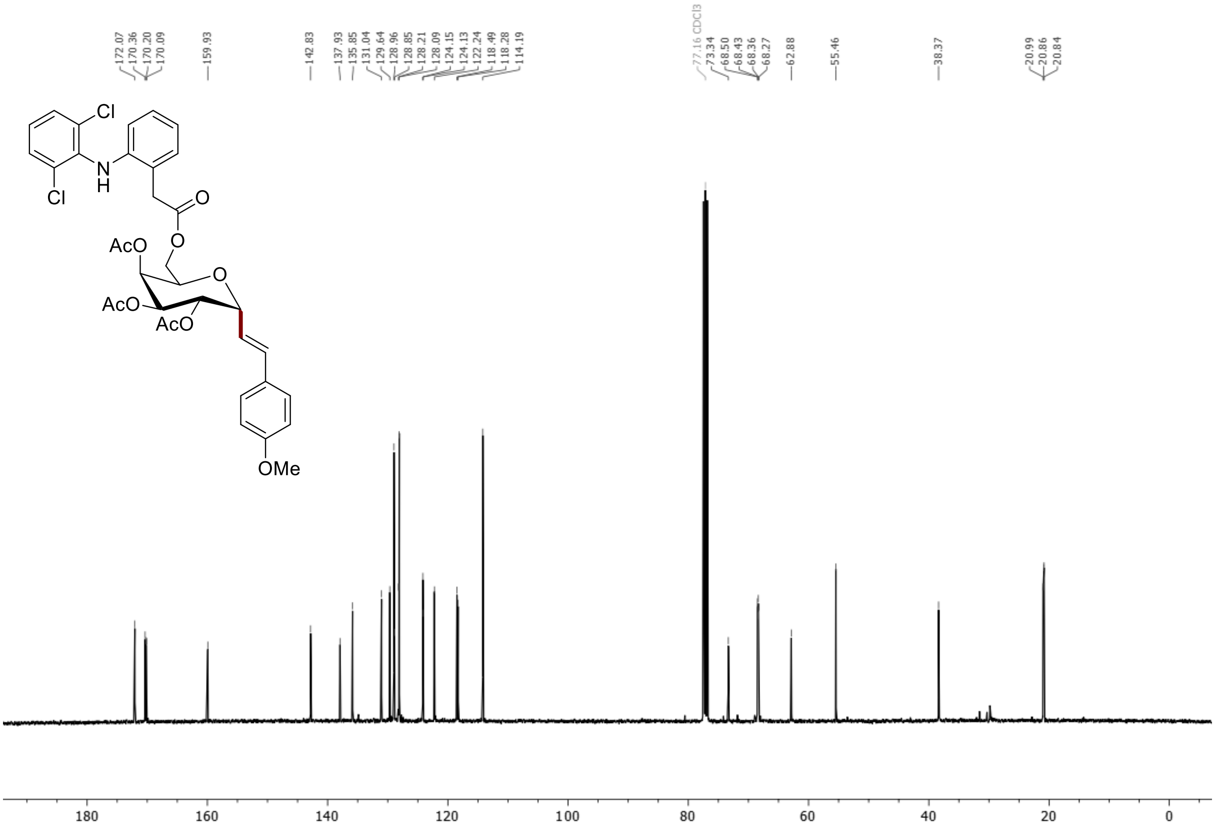

III. References

(1) (a) Doyle, L. M.; O'Sullivan, S.; Di Salvo, C.; McKinney, M.; McArdle, P.; Murphy, P. V. Stereoselective Epimerizations of Glycosyl Thiols. *Org. Lett.* **2017**, *19* (21), 5802–5805. (b) Liu, J.; Purushothaman, R.; Hinrichs, F.; Surke, M.; Warratz, S.; Ackermann, L. Synthesis of Diverse Glycosyl Bicyclo[1.1.1]pentanes Enabled by Electrochemical Functionalization of [1.1.1]Propellane. *J. Am. Chem. Soc.* **2025**, *147* (38), 34813-34822.

(2) Cai, G.; Zhou, Z.; Wu, W.; Yao, B.; Zhang, S.; Li, X. Pd-Catalyzed C(sp^3)–C(sp^2) cross-coupling of $Y(CH_2SiMe_3)_3(THF)_2$ with vinyl bromides and triflates. *Org. Biomol. Chem.* **2016**, *14* (37), 8702–8706.

(3) Kuang, C.; Senboku, H.; Tokuda, M. Facile and stereoselective synthesis of (*E*)-vinyl bromides by microwave-induced reaction of 1,1-dibromoalkenes using a diethyl phosphonate/EtONa/EtOH system. *Tetrahedron* **2002**, *58* (8), 1491–1496.

(4) Das, J. P.; Roy, S. Catalytic Hunsdiecker Reaction of *α,β*-Unsaturated Carboxylic Acids: How Efficient Is the Catalyst? *J. Org. Chem.* **2002**, *67* (22), 7861–7864.

(5) Desai, N. B.; McKelvie, N.; Ramirez, F. A new synthesis of 1, 1-dibromoölefins via phosphine-dibromomethylenes. The reaction of triphenylphosphine with carbon tetrabromide. *J. Am. Chem. Soc.* **1962**, *84* (9), 1745–1747.

(6) Abbas, S.; Hayes, C. J.; Worden, S. The 'Hirao reduction' revisited: a procedure for the synthesis of terminal vinyl bromides by the reduction of 1,1-dibromoalkenes. *Tetrahedron Lett.* **2000**, *41* (17), 3215–3219.

(7) Dolby, L. J.; Wilkins, C.; Frey, T. G. The Mechanism of the Prins Reaction. V. The Prins Reaction of Styrenes. *J. Org. Chem.* **1966**, *31* (4), 1110–1116.

(8) Chang, D.; Gu, Y.; Shen, Q. Pd-Catalyzed Difluoromethylation of Vinyl Bromides, Triflates, Tosylates, and Nonaflates. *Chem. Eur. J.* **2015**, *21* (16), 6074–6078.

(9) Pawluć, P.; Hreczycho, G.; Szudkowska, J.; Kubicki, M.; Marciniec, B. New One-Pot Synthesis of (*E*)-*β*-Aryl Vinyl Halides from Styrenes. *Org. Lett.* **2009**, *11* (15), 3390–3393.

(10) Kozikowski, A. P.; Okita, M.; Kobayashi, M.; Floss, H. G. Probing ergot alkaloid biosynthesis: synthesis and feeding of a proposed intermediate along the biosynthetic pathway. A new amidomalonate for tryptophan elaboration. *J. Org. Chem.* **1988**, *53* (4), 863-869.

(11) Liu, J.; Gong, H. Stereoselective Preparation of *α-C*-Vinyl/Aryl Glycosides *via* Nickel-Catalyzed Reductive Coupling of Glycosyl Halides with Vinyl and Aryl Halides. *Org. Lett.* **2018**, *20* (24), 7991–7995.
